# Functional DNA–Polymer Conjugates

**DOI:** 10.1021/acs.chemrev.0c01074

**Published:** 2021-03-19

**Authors:** Colette J. Whitfield, Meizhou Zhang, Pia Winterwerber, Yuzhou Wu, David Y. W. Ng, Tanja Weil

**Affiliations:** † Hubei Key Laboratory of Bioinorganic Chemistry and Materia Medica, School of Chemistry and Chemical Engineering, Huazhong University of Science and Technology, Luoyu Road 1037, Hongshan, Wuhan 430074, People’s Republic of China; ‡ Max Planck Institute for Polymer Research, Ackermannweg 10, 55128 Mainz, Germany

## Abstract

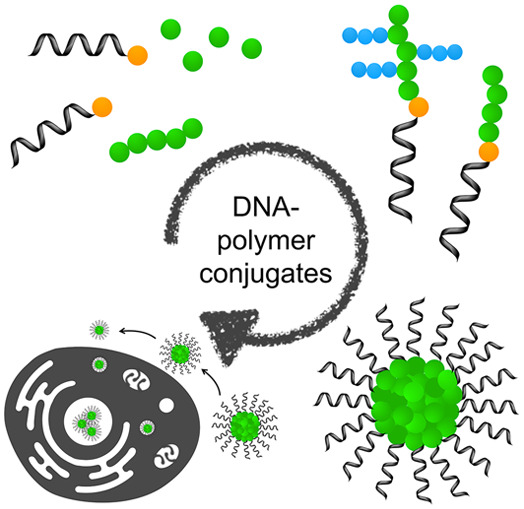

DNA nanotechnology has seen large developments over the last 30 years through the combination of solid phase synthesis and the discovery of DNA nanostructures. Solid phase synthesis has facilitated the availability of short DNA sequences and the expansion of the DNA toolbox to increase the chemical functionalities afforded on DNA, which in turn enabled the conception and synthesis of sophisticated and complex 2D and 3D nanostructures. In parallel, polymer science has developed several polymerization approaches to build di- and triblock copolymers bearing hydrophilic, hydrophobic, and amphiphilic properties. By bringing together these two emerging technologies, complementary properties of both materials have been explored; for example, the synthesis of amphiphilic DNA–polymer conjugates has enabled the production of several nanostructures, such as spherical and rod-like micelles. Through both the DNA and polymer parts, stimuli-responsiveness can be instilled. Nanostructures have consequently been developed with responsive structural changes to physical properties, such as pH and temperature, as well as short DNA through competitive complementary binding. These responsive changes have enabled the application of DNA–polymer conjugates in biomedical applications including drug delivery. This review discusses the progress of DNA–polymer conjugates, exploring the synthetic routes and state-of-the-art applications afforded through the combination of nucleic acids and synthetic polymers.

## 1 Introduction

The genetic code, one of the most prominent molecular monuments in nature, is a technological wonder from the perspective of both structural biology and macromolecular chemistry. Within this massive covalent structure twinned supramolecularly by its complementary sequence, the central dogma of biology operates with unrivalled precision that features nature’s evolutionary prowess. Chemically speaking, the genetic code is a set of colossal chains of DNA in which the diversity of life is governed through the sequence information stored within the DNA nucleobases (adenine, cytosine, guanine, and thymine).

Although its biological role and impact are clearly unambiguous, DNA has a different facade in the synthetic world—collectively known as DNA nanotechnology. Taking advantage of how the alignment of nucleotides can be woven differently with multiple intersecting chains not present in nature, nanoscale structures can be tailored with near limitless geometric possibilities. From straightforward shapes such as Y-shaped DNA-crossovers and multiarm Holliday junctions to complex folding technologies such as DNA origami, these platforms have made revolutionary advances in biophysics, photonics, nanomedicine, and materials science. This is primarily due to how DNA architectures grant the capability to position two or more (macro)molecules/nanoparticles of interest within a designated 3D space and orientation at nanometer resolution. The level of precision, coupled with the ease of DNA hybridization methods, has resulted in their widespread accessibility across all disciplines.

Nonetheless, while DNA-based technologies receive their deserved accolades within the scientific community, its relatively poor stability and restriction toward aqueous medium containing Ca^2+^/Mg^2+^ has been a glaring limitation to its potential. As such, significant attempts to stabilize DNA structures involving the conjugation of polymers, hydrophobic molecules, nanoparticles, or even higher ordered DNA weaving strategies have been achieved to protect the DNA phosphodiester bonds from hydrolysis. Interestingly, these approaches very often result in the creation of novel materials with unique characteristics and structures due to the differences between the physical properties of the DNA and its attached motif. Naturally, higher ordered architectures resulting from hydrophilic/hydrophobic interactions are among the most abundant, with morphologies including micelles, vesicles, and tubes. The dimensionality of structures from 1D to 3D can be customized by increasing the complexity of the DNA component, i.e. from single stranded DNA (ssDNA) to multiarm double stranded DNA (dsDNA) to space-filling DNA origami. By exploring the influences of synthetic (macro)molecules on a non-natural, yet geometrically precise object, exclusive lessons on self-assembly, patterning, and interactions across 3D space can be learnt.

In this respect, polymer chemistry plays a crucial role in conferring additional properties to the already broad repertoire of capabilities demonstrated by DNA. Here, the near limitless capacity for monomer design coupled with recent advances in radical polymerization methodologies under mild aqueous conditions offers a fertile avenue for the development of novel polymer–DNA conjugates in years to come. Hence, one can easily envision the overwhelming extent of possibilities fusing polymer-based technologies, i.e. block copolymers, sequence defined polymers, and immolative polymers with DNA engineering.

Furthermore, the influence of DNA technology on synthetic chemistry is not solely limited on the nanoscale. By mimicking how nature uses DNA as a template for the proliferation of life, synthetic molecules can be designed to assemble similarly along a chain of ssDNA thereby transferring the sequence information provided by the template DNA onto the newly formed synthetic polymer chain. Beyond the recruitment of small molecules or polymer precursors based on the recognition of the nucleobases, DNA can be used to template polymer synthesis by functioning as a reactive center either as an initiator or a catalyst. In general, each part of the DNA—the nucleobases, the negatively charged phosphate-deoxyribose backbone, the major/minor grooves of the double helix, as well as the 5′/3′ termini—is an attractive resource. Exploited differently, these parts of the DNA have expanded the breadth of polymer chemistry and provided alternative routes to fabricate nanoscale architectures.

## 2 Chemistries on DNA

**Figure 1:**
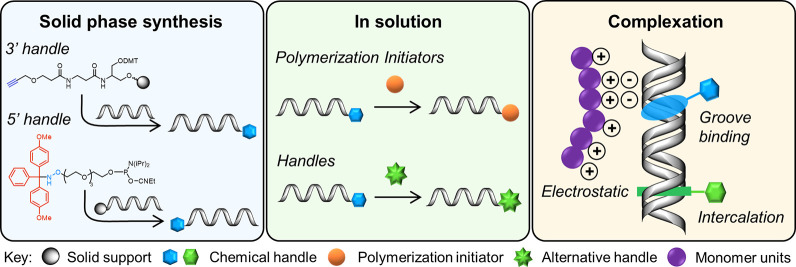
Approaches to synthesize DNA with functional handles applicable for polymer conjugation. Three approaches have been highlighted: solid phase synthesis through phosphoramidite chemistry, the subsequent in solution modifications of solid phase synthesized DNA for additional handles, and the complexation of small molecules and polymers through noncovalent interactions.

Native DNA is a rather chemically inert structure due to the lack of functional groups and the requirement to largely conserve the base-paring region to maintain function. Through the motivation of DNA nanotechnology, it can now be functionalized through the incorporation of reactive handles, typically included at the 3′/5′ termini as unnatural nucleotides or via unconventional means such as electrostatic complexation or intercalation. Consequently, the plethora of chemistries achievable on DNA has expanded and has been reviewed recently.^1^ In this section we will focus on the chemistries relevant to the synthesis of DNA applicable to DNA–polymer conjugation. Specifically, we will discuss the possible techniques to install reactive handles and the challenges to adapt each chemistry for DNA synthesis. These functional handles can be divided into different categories where the target motif can be introduced through covalent modifications or noncovalent interactions with the DNA structure (Figure 1).

### 2.1 Solid Phase Synthesis

**Figure 2:**
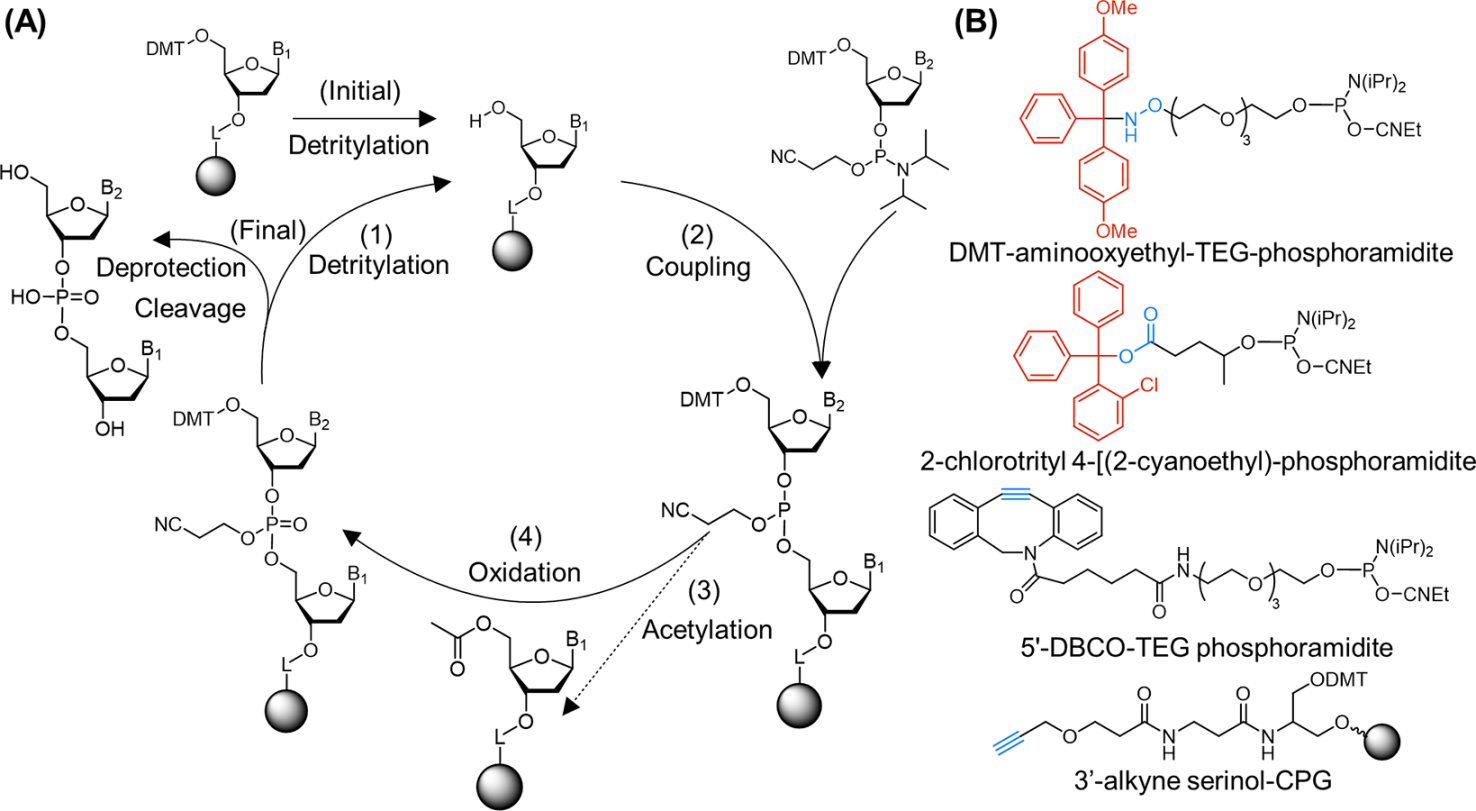
(A) Solid phase synthesis of ODNs through automated phosphoramidite chemistry on a CPG bead. (1) An initial detritylation step is required to activate the primary nucleoside for coupling. (2) Once activated the protected nucleobase phosphoramidate is added for coupling to the 5′-hydroxy of the solid bound nucleoside. (3) Some of the coupling reactions may be unsuccessful; therefore, a capping step is included. Step (4) involves the oxidation of the phosphite to phosphate and completes one cycle. The addition of nucleotides can be continued by repeating step (1) to step (4) until the ODN sequence is complete. Once complete, a final deprotection and cleavage step is performed. (B) Functionalized phosphoramidites bearing chemical handles for column modification or downstream conjugation. Two examples of protecting groups, DMT and 2-chlorotrityl, are shown in red, and each functional group (aminooxy, carboxylic acid, and alkyne groups) is highlighted in blue.

To incorporate covalent handles on DNA, depending on where the desired modification is situated, the attachment of the reactive group can be conducted during or at the end of DNA synthesis. For the synthesis of an oligodeoxynucleotide (ODN) a solid phase approach, employing phosphoramidite chemistry, is typically adopted. Phosphoramidite chemistry was first developed in the 1980s by Caruthers and co-workers and, through the optimization and employment of a solid support, resulted in the high yielding automated system used today.^2,3^ The solid support employed as the accepted standard is the controlled pore glass (CPG) bead. The CPG bead provides a high surface area to offer numerous attachment points in addition to a high stability to chemical environments.^4,5^ Polystyrene (PS) beads can also be adopted for the solid phase approach offering highly efficient synthesis at the nanomole scale.^6,7^ The solid phase synthesis method cycles through coupling, capping, oxidation, and deprotection steps for the addition of each nucleotide (Figure 2A). Once the cycles are complete, the furnished ODNs are deprotected and cleaved from the CPG using a solution of ammonia. In this way, phosphoramidite chemistry provides an approach to synthesize any sequence of DNA up to approximately 200 bases. For DNA–polymer conjugates, ODNs are often shorter than 30 bases; therefore, this method does not pose as a limitation to the length and sequences attainable.

Importantly, phosphoramidite chemistry is not limited to natural nucleotides. Internal modifications can be incorporated through modified phosphoramidites as well as modifications at the 5′-end. The chemical synthesis of ODNs is performed from 3′ to 5′; thus, 3′-end modifications are integrated through functionalized supports which the ODN chain can grow from. Modified phosphoramidites were developed alongside the described method producing varying nucleobase, sugar, and phosphate backbone moieties.^8^ Although modifications can be integrated at several positions on the nucleotide, functional handles at the 3′- and 5′-end are most relevant to DNA–polymer synthesis for the production of diblock copolymers. 5′-terminus-functionalized phosphoramidites include reactive handles such as amines,^9^ carboxylic acids,^10^ alkynes,^11^ and thiols.^11,12^ Each functional moiety must be compatible with phosphoramidite chemistry and may also require protection during the coupling process.

There are several protective groups, including dimethoxytrityl (DMT) for amines and 2-chlorotrityl for carboxylic acids (Figure 2B), which can be employed to incorporate these functional groups. Several moieties can be incorporated without protection and can therefore be readily modified “on column”—an advantageous attribute to grant access toward solid phase polymer coupling. Alkyne moieties, such as dibenzo-cyclooctyne (DBCO), a strained alkyne capable of copper free click chemistry, are incorporated at the 5′-end, and standard unstrained alkyne groups can be included at the 3′-terminus through bead modifications prior to the solid phase synthesis (Figure 2B). Hydrophobic and hydrophilic linkers are available in the form of alkyl chains and ethylene glycol units, respectively, to link the described functional handles to the phosphoramidite.

The incorporation of the functional groups described above into the DNA makeup provides an avenue to synthesize DNA for conjugation to preformed polymers. Where polymerization directly from DNA is desired, the polymerization initiators, agents or monomers, must be attached prior to polymerization. Atom transfer radical polymerization (ATRP) initiator phosphoramidites are not available commercially; however, several can be synthesized and have been incorporated through solid phase synthesis prior to deprotection and cleavage, demonstrating a feasible method to attach initiator moieties to ODNs.^13,14^ A two-step reaction can conjugate the initiator group to the phosphoramidite moiety, now available for solid phase attachment, followed by cleavage and deprotection in ammonia. This method provides an automated route to synthesize ODNs bearing ATRP initiators. However, the attachment of reversible addition–fragmentation chain transfer (RAFT) agents prior to deprotection and cleavage is not possible due to its instability in ammonia. Similarly, the norbornene-phosphoramidite is also not available commercially; however, its synthesis and consequent incorporation has been established.^15^ In this case, two modified nucleoside phosphoramidites as well as the 3′-functionalized column were synthesized demonstrating the versatility and ability to choose the position of the norbornene moiety. Modifications in the base pair region may not be optimal due to conformation dynamics,^16^ in addition to sterics and charge repulsion from the overall DNA structure. Thus, to ensure the functional group is positioned externally (i.e., protruding the major or minor groove) on the DNA structure, the 5-position on cytosine and the 4-*O*-position on thymidine were adopted for the modification. These developments achieved through phosphoramidite chemistry have enabled the initial vision and future realization of covalent DNA–polymer synthesis.

### 2.2 In Solution

**Figure 3:**
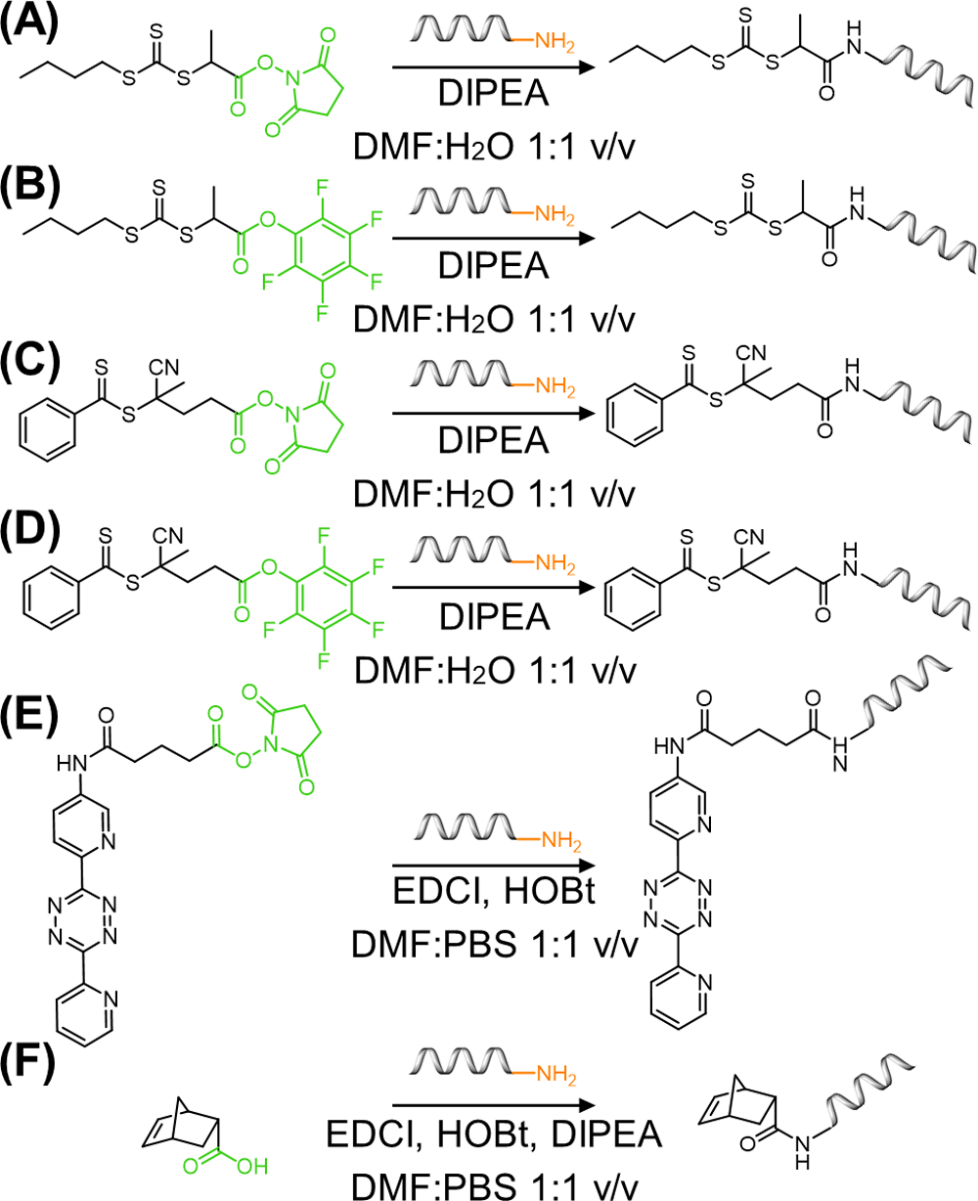
Solution-based modification of ODNs for functional handle attachment. (A)–(D) The attachment of RAFT agents, (((butylthio)carbonothioyl)thio)propanoic acid (BTPA) and 4-cyano-4-(phenylcarbonothioylthio)pentanoic acid (CPADB), to ODNs through amide coupling chemistry with NHS and pentafluorophenol (PFP) activated carboxylic acids. (E) NHS-activated coupling of tetrazine to amine DNA. (F) Amide coupling of norbornene-carboxylic acid with amine DNA. Coupling reagents include diisopropylethylamine (DIPEA), 1-ethyl-3-(3-(dimethylamino)propyl)carbodiimide (EDCl), and 1-hydroxybenzotriazole (HOBt), and solvents include dimethylformamide (DMF) and phosphate buffered saline (PBS).

For several functional groups, such as RAFT agents, the corresponding phosphoramidite is either not commercially available or is not compatible with the solid phase synthesis process. However, the chemical handles available through solid phase synthesis can be postmodified after column cleavage to position the unattainable groups. Although the chemistry itself is simpler than the synthesis of a phosphoramidite, unprotected DNA is a polyelectrolyte and requires an aqueous solvent system (e.g., a Tris buffer of pH 8), which can present a new challenge. However, if organic solvents are required for the coupling reaction, surfactants can be employed through complexation to mitigate DNA’s incompatibility with hydrophobic compounds.^17^ Many coupling reactions have now been demonstrated on functional handles, such as amines, thiols, and alkynes, which were previously incorporated during solid phase synthesis. As native DNA does not bear specific sites for chemoselective reactions, these compatible handles must be incorporated prior to column cleavage through the phosphoramidite chemistry described above. The conjugation of these functional ODNs with small molecules (for example, fluorophores) has enabled the establishment of common procedures and reagents for coupling in the presence of unprotected DNA.^1^ For a more efficient conjugation of DNA to polymers, several moieties are of interest that are not available as phosphoramidites for solid phase synthesis. For instance, norbornene–tetrazine chemistry was established as an efficient self-reporting method for DNA–polymer conjugation; therefore, the modification of a reactive ODN to bear these specialized functions was desired.^18^ Both functional groups are not available as a phosphoramidite commercially (although the synthesized ODNs are now available); however, the synthesis in solution has been demonstrated (Figure 3).^18^ The reactions were performed in a dimethylformamide (DMF)–phosphate buffered saline (PBS) 1:1 v/v solution to ensure solubility and stability of both the unprotected ODN bearing a carboxylic acid or *N*-hydroxysuccinimide (NHS) functional handle and the small molecules.^18^ In this case, the now adapted functional end-groups were available for direct conjugation with a presynthesized polymer.

The examples described so far document the secondary modification of native DNA to bear functional handles for covalent conjugation of DNA with presynthesized polymers. For polymerization to occur from DNA (*grafting from* approach), the polymerization initiator or agent must be anchored to the DNA structure. Although the synthesis of ODNs bearing ATRP initiators has been realized through phosphoramidite chemistry, in contrast, RAFT agents cannot be conjugated prior to the deprotection and cleavage steps. Postmodification cannot take place on the solid support and must be conducted in solution after cleavage. This synthesis was demonstrated through the postmodification of amine DNA with NHS or pentafluorophenol (PFP) activated-RAFT agents, i.e. (((butylthio)carbonothioyl)thio)propanoic acid (BTPA) and 4-cyano-4-(phenylcarbonothioylthio)pentanoic acid (CPADB) (Figure 3).^19^ Such reactions were each performed in a DMF–PBS 1:1 v/v solution and demonstrated efficient yields to position RAFT agents on ODNs. These methods demonstrated the ability to synthesize ODNs bearing a wide range of functional groups for either direct polymer conjugation or growth through RAFT polymerization, aiding the widespread development of DNA–polymer function and application. Nonetheless, the examples described here each adopt an amine-functionalized ODN and therefore do not explore the plethora of coupling chemistries available to position functional groups not available as phosphoramidites. Through the continuous expansion of click chemistry and bioconjugation, the possibilities for ODN functionalization with synthetic macromolecules can be perpetually expanded.

Additionally, in this section we have highlighted the approaches adopted for reported conjugations, which each require a functional handle from solid phase phosphoramidite synthesis. However, the functionalization of DNA is not limited to this method. Chemical handles can also be incorporated through DNA polymerase extension with modified deoxynucleotide triphosphates (dNTPs). The employment of modified dNTPs opens an alternative toolbox to incorporate non-native functional groups through enzymatic synthesis.^20^ Although this approach has not been employed for DNA–polymer synthesis, efficient incorporation and subsequence coupling has been established,^21^ demonstrating an opportunity for alternative conjugation methods with potentially improved yields and diversity.

### 2.3 Complexation

**Figure 4:**
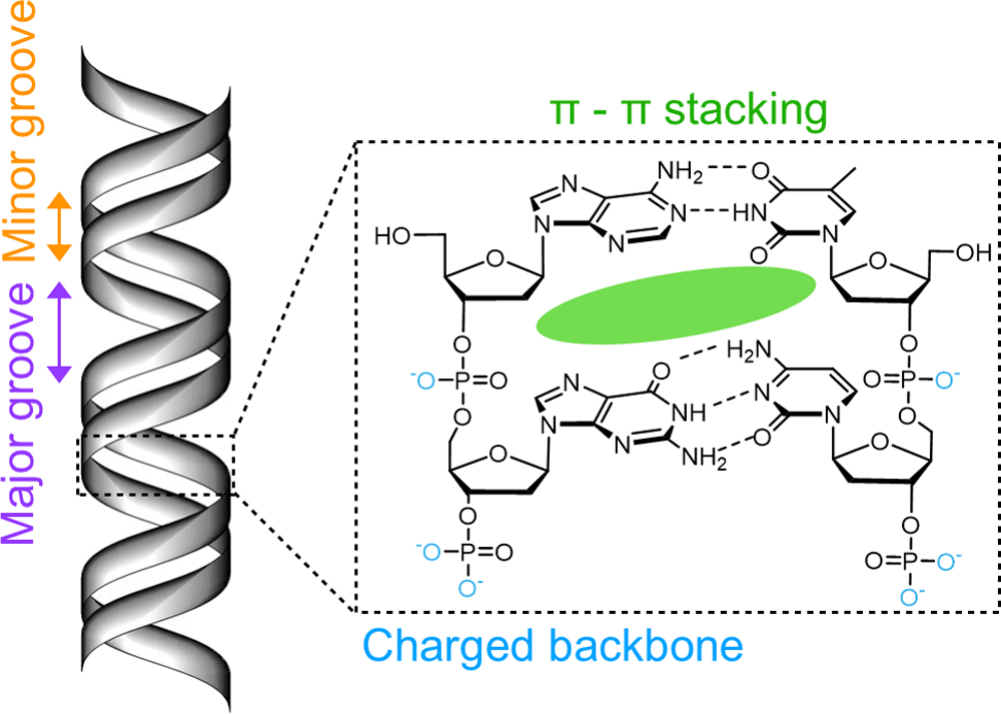
Noncovalent complexation sites of dsDNA. The minor and major groove, base stacking, and charged phosphate backbone have been highlighted.

In addition to the portfolio of covalent chemistries available to the reactive groups of DNA, noncovalent approaches exploiting the structural elements of DNA offer an alternative route for DNA functionalization. Native dsDNA is a highly charged molecule, formed through many noncovalent interactions which can be exploited for noncovalent complexation. ssDNA forms the duplex through hydrogen bonding and van der Waals forces, π–π stacking, and hydrophobic effects in addition to the entropically favorable disorder of water molecules. These interactions present opportunities for noncovalent dynamic binding of small molecules to the major and minor groove, between base pairs and to the phosphate backbone (Figure 4). Through these binding modes, there is the potential for noncovalent interactions to be used to anchor functional groups as well as to complex whole polymers. In contrast to the covalent conversions described above, noncovalent complexation is a highly dynamic assembly that does not require chemical modifications to the intrinsic DNA makeup.

The capability to employ electrostatic interactions with the charged backbone generates a simple method for cationic molecules to bind to the sterically available anionic groups on DNA. The charged backbone plays many important roles in nature, such as guiding proteins and ligands to designated positions,^22^ for example through the supramolecular assembly of DNA with the positively charged histone protein. These intrinsic interactions inspired the employment of the phosphate backbone for DNA–polymer conjugate synthesis. To afford this interaction, a reduction in ion–ion repulsion is required where stabilization with Group 1 and 2 counterions is commonly used. Thus, equally for the interaction with polymers, ion displacement must occur. A huge charge repulsion must be overcome in comparison to other biological molecules, such as proteins, which are commonly neutral or have low charge counts. This is evidenced by the thermal energy required to bring two DNA molecules into proximity where electronic repulsion is 100× increased without the presence of counterions.^23^ Due to the dynamic nature of DNA interactions, it is difficult to study the ion sphere to understand precise interactions;^23^ however, the Poisson–Boltzmann equation can be employed to describe the relationship between a charged molecule and the counterions in solution to provide information about the ion–ion interactions.^24,25^ Overall physical properties of the DNA–polymer product, such as zeta potential and morphology, can also be analyzed to predict the complexation interactions. The interaction of polycationic polymers with DNA has gained interest due to the increased ambition to deliver DNA to cells as potential therapeutics. The dissociation of DNA–polycations through the addition of counterions can probe the effect of ionic strength on the polymer interactions.^26^ Several counterions of varying anion and cation units were added to DNA–polycation complexes, revealing the Group 2 ions, Ca^2+^ followed by Mg^2+^, as the strongest dissociators in comparison to the Group 1 ions. Anion competitors were also studied showing the larger and less electronegative I^–^ caused the greatest effect on polymer dissociation followed by Br^–^, Cl^–^, and F^–^.^26^ Conversely, the study of polymer binding has also been performed exposing an important note—the binding of cationic polymers to DNA reduces the overall charge and thus alters the hydrophobicity.^27^ Therefore, balancing the concentration of cationic polymer units to DNA’s anion charges has crucial implications for solubility and aggregation. Full neutralization of charge leads to DNA condensation, which, depending on the application desired, can have implications, such as steric hindrance of reactive sites. Similarly, the pH has large consequences on binding strength and, accordingly, the ability to form complexes.^28^ A lower pH can yield a higher degree of binding as observed by the smaller and more tightly packed morphology in comparison to the larger structures observed at a higher pH—a lower pH yields a higher extent of ionization.^28^ In addition to the ion displacement, the shape of the polymer also has an effect on DNA complexation.^29^ The work of Tang and Szoka employed several polymers of similar molecular weight but varying degrees of branching to investigate complexation with DNA.^29^ Interestingly, the unordered branched polyethyleneiminie yielded average complex diameters of 90 nm, which is approximately 5% of the linear polylysine complex average diameter of 2000 nm. Thus, the shape can dictate both the polymer packing and the condensation of DNA. An understanding of the structure and charge effects of cationic polymer binding to DNA can aid the design and choice of the respective polymer to avoid undesired structure deformation and to ensure applicability for the desired function.

Groove binders have become a major target for small molecule and protein binding for therapeutic action.^30^ Many natural products have been discovered that offer native antibacterial or anticancer properties through groove binding and grant insight into structural qualities appropriate for association.^30^ Through the desire to understand the interactive pockets, the precise interactions have been revealed and can therefore be utilized for future therapeutic designs. Groove binders can target either the major or minor groove (Figure 4) through several noncovalent interactions, consisting of hydrogen bonding and van der Waals and electrostatic interactions. Each base pair provides a different environment through the varying electrostatic effects, groove width, and depths. Therefore, selective binding can be employed; for example, small molecule binding tends to prefer AT rich regions due to the increase in van der Waals forces provided through the deeper pocket.^30^ Additionally, the minor groove offers a tighter pocket, attracting small molecules or polymer chains bearing small monomer units, such as poly(pyrrole) and polyamides,^31^ that are either cationic or neutral.^32^ Due to the many interactions possible, binding is afforded through several mechanisms. Specific interactions include H-bonding with the sugar C1, purine N3, and pyrimidine N1 as well as the base pairing moieties.^32^ Additionally, shape selective binding due to molecular curvature is also apparent, where molecules match that of the native DNA structure.^30^ All these parameters brought together lead to a high degree of target specificity. Larger molecules, such as proteins and carbohydrates, recognize and bind in the major groove. Although there are more donor and acceptor sites in the major groove providing the platform for stronger overall enthalpic interactions, fewer natural examples of major groove binders are described.^33^ Aminoglycosides are nonaromatic molecules which preferentially bind to the major groove of B-DNA due to the dimensions and hydrogen bonding opportunities.^31^ Although initial interactions may be with the phosphate backbone, studies employing a triplex DNA structure demonstrated the competitive release of the third strand on the addition of an aminoglycoside dimer, implying major groove binding of the aminoglycoside structure.^34,35^ A fundamental interaction is the protein–DNA dynamic binding with the major groove. In this case, the noncovalent H-bonds and salt bridges allow a reversible binding and release for processes, such as transcription and gene regulation. The functional groups on the bases and ribose sugar provide several H-bond donor and acceptor sites. A detailed analysis of structure relationships has been reviewed previously by Thornton and co-workers.^36^ Although current approaches to DNA–polymer conjugation do not directly employ groove binding, understanding the interactions will guide future designs to improve polymer interactions through structure optimization as well as positioning groups for functional anchors along the backbone. Proteins and aminoglycosides both offer many H-bonding sites in addition to positively charged residues to overcome repulsive forces. Through this knowledge, polymer design can be molded to encompass these attributes. However, it is important to also consider the structural distortions groove binding can have on the B-DNA structure. Groove binders that possess a strong overall binding enthalpy that outweighs the conformational changes can induce a fit.^37^ Depending on the specific application of the DNA, these structural changes may hinder downstream interactions.

While the backbone and grooves offer external interactions with DNA, the structure also offers the conformational flexibility to exploit the base pair stacking to complex small molecules within. π–π stacking interactions between planar aromatic purine and pyrimidine rings and aromatic molecules are possible and have been discovered in many natural products.^42^ Natural product functions have consisted of several inhibitory roles which may act through allosteric interference of protein binding,^43^ influencing the development of anticancer drugs.^44^ Similar to groove binding, intercalators can cause conformational changes, such as extension. This extension is useful to determine binding through length changes; however, it may also alter recognition and function of DNA as a genetic material.^45^ Intercalators, forming a mono- or bis-intercalation between one or over two base pairs, respectively,^46^ have been developed either for anticancer agents or as fluorescent dyes to visualize or quantify DNA.^44,47^ Several key features aid the association, such as a positive charge as present on ethidium bromide (Table 1) and three or four conjugated rings. As well as the stacking interactions, complementary dipoles can also increase association strength. The aromatic nature provides a plethora of reaction conditions to perform substitution reactions to anchor reactive handles on the intercalator backbone.^48^ These substitution reactions can yield reactive handles for polymer coupling prior to intercalation allowing the possibility of direct noncovalent conjugation of preformed polymers throughout the DNA duplex.^40^ Prior to polymerization, a two-step synthetic approach was demonstrated employing 9-chloroacridine as the starting material to yield the polymerization-agent bearing acridine intercalator (an example acridine compound is shown in Table 1). Polymerization from the functionalized acridine could then be performed followed by DNA intercalation. Intercalation was noted with each polymer–acridine conjugate; however, there was an effect on the association constant depending on the polymer employed (Table 1). The authors attribute this effect to the molecular weight and structure of the polymer where varying hydrophobicity and side-chain makeup have been explored.^40^ An alternative intercalator is psoralen, a 3-ringed furanocoumarin monointercalator (Table 1), commonly adopted to cause mutagenesis under ultraviolet (UV) light.^49^ Psoralen intercalation occurs preferentially through thymine interactions, although the presence of substituents can shift the precise positioning.^50^ Similarly to acridine, functional handles can be positioned to provide anchors for conjugation of polymers. Specifically, a trimethylpsoralen was functionalized with a terminal amine to afford amide conjugation with an NHS polymer.^38,39^ Once conjugated, the psoralen can intercalate with the dsDNA, yielding a noncovalent DNA–polymer interaction. So far in this section, the two examples have demonstrated the direct assembly of polymers with DNA through covalent polymer conjugation with an intercalator. Although binding was noted in each case, a reduction in association strength was also exhibited.^40^ To ensure efficient binding, an alternative approach where intercalators bearing functional handles are assembled with DNA prior to polymer conjugation can maintain binding strengths. This was demonstrated with proflavin, an acridine derivative, which can undergo modification to produce a diazide, positioning the functional handles in the major groove.^41^ The addition of these functional groups reduced the binding by 10-fold (Table 1). However, by a further modification to produce methyl proflavindiazide, the binding strength is returned to the same magnitude as the unmodified proflavin.^41^ Once intercalated, the click reaction is then feasible with alkyne-bearing molecules, such as the 5-pentynyl-thienyl-pyrrol monomer.^51^ By positioning the polymerizable monomer in the major groove, templated polymerization along the DNA backbone can now be envisaged.

**Table 1:**
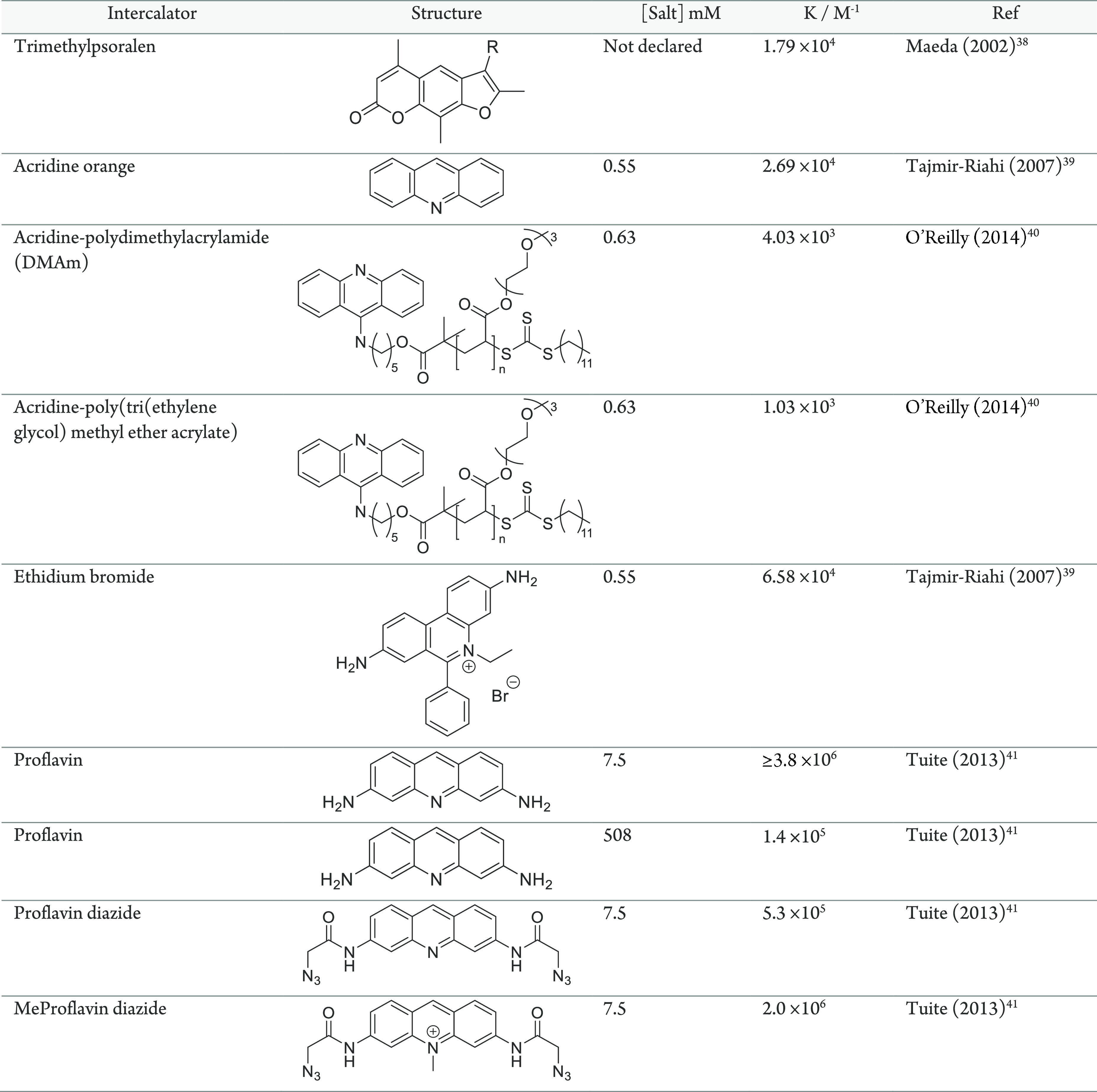
List of Intercalators and Their Binding Strength

In the complexation interactions described above, each mechanism is explored individually; however, for several DNA binders, multiple interactions are involved. A commonly adopted example is the combination of intercalation and groove binding of antibiotics bearing peptide groups which reside in the minor groove.^52^ Triple interactions have also been noted; for example, the conjugate neomycin-Hoechst 33258 pyrene exhibits a neomycin major groove interaction, a Hoechst 33258 minor groove interaction, and a pyrene-intercalator.^53^ Importantly, the introduction of conjugate moieties increased the binding constant up to 10-fold in comparison to the individual small molecule (in this case, Hoechst 33258).^53^ Therefore, attention to the multifaceted noncovalent design of DNA–polymer conjugates would increase binding strength and thus has potential to prolong complex stability. The interactions noted for intercalator–conjugate assemblies lay the foundation for intercalator–polymer design to guide the synthesis of precise polymeric nanostructures.

## 3 DNA–Polymer Synthesis

**Figure 5:**
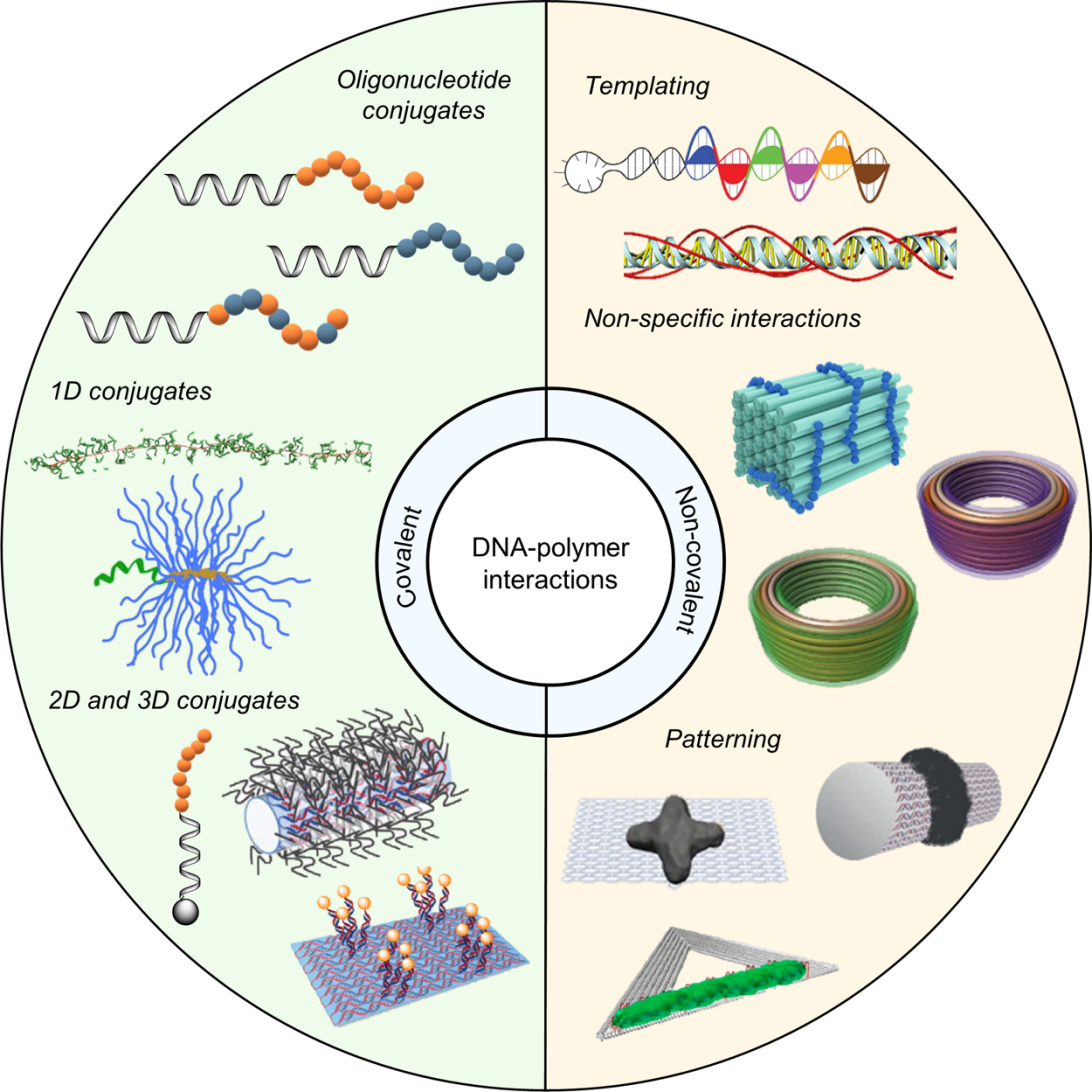
DNA–polymer conjugate synthesis summary. Conjugates are categorized as covalently or noncovalently bound. Covalently bound structures can be conjugated either in solution by combining an oligonucleotide with either a linear polymer or a polymer brush.^54,55^ Reproduced with permission from ref (54). Copyright 2016 American Chemical Society.^55^ Reproduced with permission from ref (55). Copyright 2015 American Chemical Society. Solid supports, such as beads and DNA nanostructures, can also be adopted to provide a platform for the conjugation.^56,57^ Reproduced with permission from ref (56). Copyright 2018 the Royal Society of Chemistry. Reproduced with permission from ref (57). Copyright 2016 John Wiley and Sons. Alternatively, the conjugates can form through noncovalent interactions, such as templating.^58,59^ Reproduced with permission from ref (58). Copyright 2011 the Royal Society of Chemistry. Reproduced with permission from ref (59). Copyright 2013 Springer Nature. Nonspecific interactions through complexation in addition to patterning of polymers on DNA are also possible.^60−64^ Reproduced with permission from ref (60). Copyright 2016 the Royal Society of Chemistry. Reproduced with permission from ref (61). Copyright 2017 Springer Nature. Reproduced with permission from ref (62). Copyright 2018 John Wiley and Sons. Reproduced with permission from ref (63). Copyright 2014 American Chemical Society. Reproduced with permission from ref (64). Copyright 2020 John Wiley and Sons.

**Figure 6:**
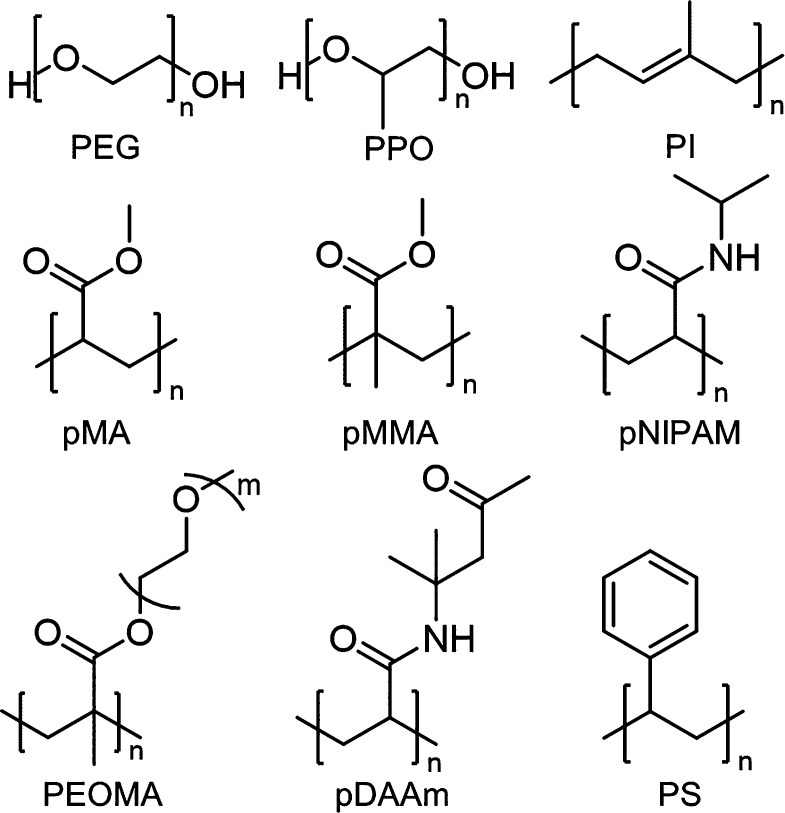
Examples of commonly used polymers for DNA–polymer conjugates: PEG, poly(ethylene glycol); PPO, poly(propylene oxide); PI, poly(isoprene); pMA, poly(methyl acrylate); pMMA, poly(methyl methacrylate); pNIPAM, poly(*N*-isopropylacrylamide); PEOMA, poly(ethylene oxide methyl ether methacrylate); pDAAm, poly(diacetoneacrylamide); PS, polystyrene.

Polymerization was first noted in the 1800s and has since developed to produce the synthetic polymers commonly used today, such as PS and Nylon (Figure 6). Due to the structural prospects, diblock copolymers have gained growing interest and can be designed to form many nanostructures, such as micelles and vesicles. Through the advancements of living polymerization techniques, polymer length dispersity is now reduced and has enabled the synthesis of copolymers for lithography and many controlled nanostructures. Combining DNA with synthetic polymers enriches functional properties through the combination of the hydrophobic/hydrophilic nature of the polymer and the ease of further functionalization through the complementary DNA sequence. DNA is a highly programmable entity with a plethora of structures, providing the platform to control the synthesis of polymers as well as their spatial organization. Here, we will discuss the recent advancements, the challenges, and possible solutions to synthesize DNA–polymer conjugates. DNA–polymer conjugates can be categorized through their interaction, either covalent or noncovalent, and through the DNA structure, from ODNs through to nanostructures, such as DNA origami (Figure 5).

### 3.1 Covalent DNA–Polymer Conjugates

There have been large developments in the synthesis of covalent DNA–polymer conjugates; however, several limitations have hindered progress. We will first introduce the polymerization methods employed for DNA–polymer conjugate synthesis and highlight the limitations of these methods in addition to the challenges of combining synthetic polymers with DNA. Through this discussion, we can build a greater understanding of the progress made in this field through solution- and platform-based conjugation methods which are described in this section.

#### 3.1.1 Polymerization Methods

**Figure 7:**
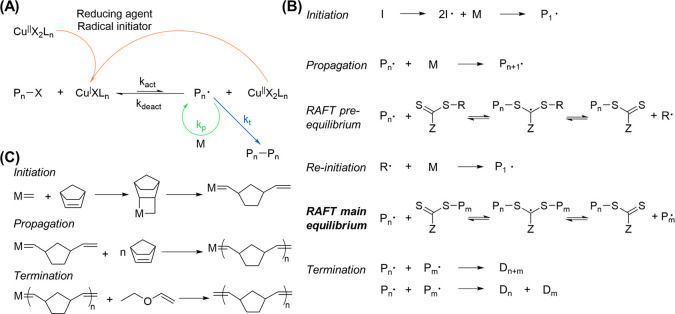
Living polymerization techniques appropriate for DNA–polymer synthesis. (A) Schematic of Cu-catalyzed ATRP. The transition metal catalyst, here Cu, is reduced to activate and initiate the radical. Polymer propagation (*K*_p_) occurs through radical polymerization of reactive monomers. Termination (*k*_t_) proceeds through the combination of reactive polymers. Catalysts are oxidized through the activation step and can deactivate either through the more prominent deactivation or by the reducing agent. The equilibrium between activated (*k*_act_) and deactivated (*K*_deact_) states is determined by the catalyst used. (B) RAFT polymerization mechanism where I = initiator, M = monomer, P = polymer, Z = radical stabilizing group, and D = dead polymer. (C) ROMP employing a metal catalyst for coordination to a strained alkene for olefin metathesis. Termination can be performed by the addition of ethyl vinyl ether to coordinate to and remove the metal catalyst.

There are several polymerization methods applicable to DNA–polymer conjugates, including anionic, cationic, ring-opening, and free radical polymerizations. Free radical polymerizations are most commonly adopted for linear polymer synthesis for DNA–polymer conjugates where the equilibrium required to accomplish reduced mass dispersity was first demonstrated through ATRP. ATRP was invented in 1995 and employs an alkyl halide as the initiator along with a redox-active catalyst (Figure 7A).^65,66^ Here, the equilibrium is determined by the rate of activation and deactivation of the propagation reaction, where deactivation must be greater than activation to maintain a low concentration of radical species. The first examples of ATRP required a metal catalyst, which initially led to developments involving reducing agents to reactivate the metal center to reduce the required metal concentration; however, it could not be removed entirely. Metal free ATRP was later developed and employs an organic redox-active catalyst, therefore reducing the biological toxicity of the reaction and increasing the compatibility of ATRP for DNA conjugation.^67^ RAFT polymerization was developed shortly after ATRP and is also performed metal free. RAFT proceeds by a radical polymerization mechanism in the presence of a chain transfer agent (CTA) to afford the necessary equilibrium for reduced mass distribution (Figure 7B). The added chain transfer step redistributes the radical to allow an equal probability for all chains to grow. Importantly, RAFT polymerization end-group chemistry is readily available through the liberation of the thiol group in the transfer agent. Although ATRP and RAFT are the most prominent, ring-opening polymerizations (ROPs), such as ring-opening metathesis polymerization (ROMP), have also been applied to polymer synthesis for the production of DNA–polymer brush structures. ROMP occurs through olefin metathesis of a strained alkene, which drives the reaction (Figure 7C). Here, a metal catalyst is employed to form an open coordination with the alkene followed by a [2 + 2] cycloaddition. The catalyst, again, provokes challenges for purification and side reactions.

**Figure 8:**
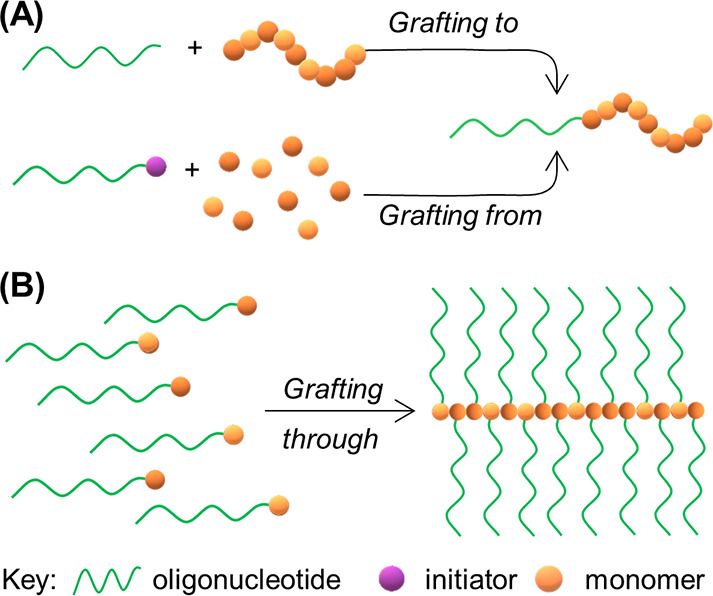
Common approaches to synthesize DNA–polymer conjugates. (A) *Grafting to*, i.e. polymerization in isolation from DNA, prior to covalent attachment and *grafting from*, i.e. polymerizing from an initiator covalently attached to the DNA. (B) *Grafting through*—polymerization of monomers either with the ODN already conjugated or with a functional group for postpolymerization conjugation.

The synthesis of covalently bound DNA–polymer conjugates has seen large developments, now enabling the controlled synthesis of diblock copolymers consisting of many combinations of polymers and DNA nanostructures. The synthesis of DNA–polymer conjugates can be categorized into three methods: *grafting from*, *grafting to*, and *grafting through* (Figure 8). *Grafting from* occurs when the polymerization initiator is covalently bound to the DNA followed by *in situ* polymerization, whereas for *grafting to*, the polymer and DNA parts are presynthesized prior to conjugation. *Grafting through* encompasses the polymerization of macromonomers bearing a polymerizable group to synthesize polymers with defined side chains. Each approach bears advantages—*grafting from* exhibits the greatest attachment chemistry and therefore largest density,^68^ whereas *grafting to* allows thorough polymer characterization prior to conjugation and polymer choice is broader (the polymerization occurs in the absence of DNA—the reaction can occur in larger scales, in many solvents, and using different monomers). *Grafting through* is employed less frequently; however, it can efficiently synthesize many brush or hyperbranched structures. Nonetheless, each approach has drawbacks to either the yield or breadth of polymer conjugates achievable. These drawbacks can be accounted for by both the use of DNA in this system and also the polymerization conditions.

#### 3.1.2 DNA–Polymer Conjugate Synthesis Limitations

Through the advancement of the polymerization methods described above, polymer synthesis, itself, is a highly established technique, which has been optimized for many monomer and polymer types. In parallel, the expansion of bioorthogonal chemistry has provided a plethora of conjugation reactions between modified DNA and a variety of molecules, providing ample resources for DNA to polymer conjugation reactions. However, for DNA–polymer conjugates, there are several limitations due to the combination of these two materials in one reaction pot because they can each provide contrasting properties. In the approaches discussed here, DNA is present either in the conjugation reaction (*grafting to*) or in the polymerization reaction (*grafting from*). DNA is a highly ionic molecule requiring an aqueous environment which is readily compatible with hydrophilic monomers and polymers; however, hydrophobic monomers and polymers require a solvent mixture to enable solubility. Organic solvents are commonly poor liquids for DNA, altering hydrogen bonding, polarity, and hydrophobicity.^69^ Specifically, solvents consisting of longer or alkyl-substituted chains cause the greatest disruption.^70^ Consequently, initial studies employing the *grafting to* approach reported low yields for the conjugation of hydrophobic polymers to DNA.^71^ However, as hydrophobic polymers also pose great interest, several groups have established improved methods such as DNA protection with counterions or sophisticated coupling chemistries.^17,18^ A thorough investigation into possible coupling reactions between DNA and poly(*N*-isopropylacrylamide) (pNIPAM) was performed by O’Reilly and Wilks.^18^ They found amine coupling and thiol–ene Michael addition reactions did not synthesize the correct product in organic solvents or were not reproducible. In each case, several solvents were trialed including DMF, dimethyl sulfoxide (DMSO), acetonitrile (ACN), and tetrahydrofuran (THF).

Similarly, the *grafting from* approach also favors hydrophilic monomers. An example employing DMSO as the solvent to polymerize methyl acrylate established a method for successful polymerization.^14^ Polymerization induced self-assembly (PISA) can also overcome this challenge by the polymerization of hydrophilic monomers to produce hydrophobic polymers.^72,73^ The use of PISA has been employed to successfully produce DNA–hydrophobic polymer conjugates through the *grafting from* approach.^74^

In addition to solvent compatibility, both blocks of the DNA–polymer conjugate are flexible polymers and can therefore shield the reactive moiety. Steric effects are observed when coupling to all forms of DNA—ss, ds, and nanostructures—although the effects are different for the solution-based (ss and ds) and solid support (nanostructures and DNA origami) forms. Additionally, the sequence of ssDNA requires a fine design to ensure the secondary structures do not hinder the reactive site. This also applies to dsDNA where the duplex may be in equilibrium with higher ordered structures. In both ss- and dsDNA, the sequence can be designed and modeled to ensure that inhibitory secondary structures are avoided. Conjugation to DNA origami presents the greatest hindrance for conjugation. The DNA origami not only burdens the reaction center with steric hindrance, it also, where multiple sites are present on one structure, reduces the distribution of reaction sites in solution and requires a higher local concentration on the origami. This causes drawbacks for both approaches; however, *grafting from* is deemed preferable to synthesize DNA origami–polymer conjugates as the steric hindrance is reduced.^57^ Steric effects are also a large consideration when coupling to a preformed polymer, i.e. *grafting to*. In this case, the larger polymers may shield the reactive handle and therefore reduce the reaction process.

Although the limitations described so far are mainly attained from the *grafting to* approach, the *grafting from* technique performs the polymerization in the presence of DNA, which produces additional challenges. When handling DNA, small volumes are typically employed due to limited resources (reactive group-bearing oligos are commonly produced in microgram quantities); thus, when *grafting from*, small volumes are also adopted for the polymerization process. This limitation is mainly apparent as both RAFT and ATRP techniques are oxygen sensitive and therefore require an anaerobic environment. The approximate length of polymers can be controlled by the monomer to transfer agent or initiator ratio; however, oxygen is a radical scavenger and can therefore quench the initiated or transferred radical, altering the ratio. Radical polymerization in the absence of DNA (i.e., polymerizations performed prior to conjugation and not employing the *grafting from* approach) can be performed in large volumes and is therefore not limited through the available techniques to remove oxygen. The most effective method to remove dissolved oxygen is through N_2_ purging.^75^ N_2_ purging is possible in large scale synthesis; however, *grafting from* DNA is commonly performed in less than 300 μL, preventing the efficient use of purging. Similarly, the freeze–pump–thaw technique, whereby the solution is frozen before a vacuum is applied to reduce the dissolved oxygen solubility, can take place in larger volumes, i.e. 1 mL. However, this technique is again problematic when performing the polymerization in small volumes, i.e. <300 μL, in the *grafting from* approach where the DNA concentration is limited. Volume loss may compromise reproducibility due to the effects residual oxygen will have on the polymer length and yield. Additionally, DNA degradation can occur when the sample is subjected to repeated freezing and thawing—tension forces are generated from ice crystals and may lead to strand breakage.^76^ An alternative method is enzyme degassing—a technique that enables oxygen sensitive polymerization in air. Glucose oxidase, an enzyme that converts oxygen to hydrogen peroxide, can perform successful enzyme degassing for RAFT polymerization in an open, low volume vessel *grafting from* ODNs.^77^ Enzyme degassing provides an avenue to explore a wider range of polymers synthesized through the *grafting from* approach in the presence of DNA and in small volumes. However, purification to remove the enzyme is required after the reaction if downstream processes are desired. There are also other challenges associated with the reduced concentrations available when working with DNA. Again, polymerization in isolation from DNA can be performed as optimized; however, when reactions with DNA for conjugation or polymerizations from DNA are required, optimal concentrations may not be possible with the limited amount of DNA (Table 2). This is more notable when *grafting from* DNA origami. DNA origami is commonly synthesized in low volumes (less than 100 μL) and in low concentrations (approximately 50 nM). Polymerizations are optimal at mM concentrations; thus, to overcome this, sacrificial initiators are required in solution to ensure the concentration limit is reached.^57^ Although this allows the reaction to proceed, polymerization also takes place in solution, adding competition to the DNA origami surface polymerization leading to downstream purification challenges.

**Table 2:**
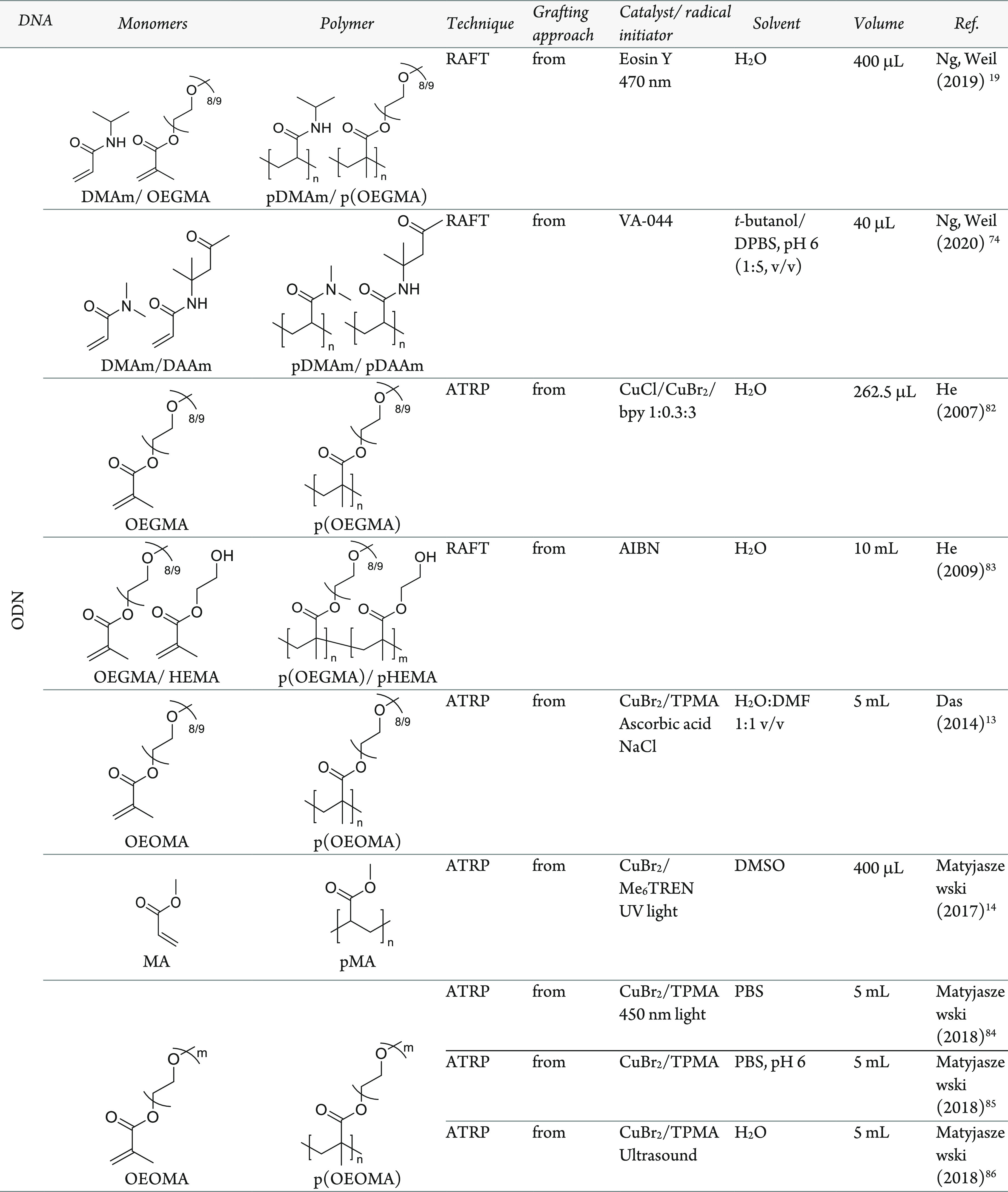

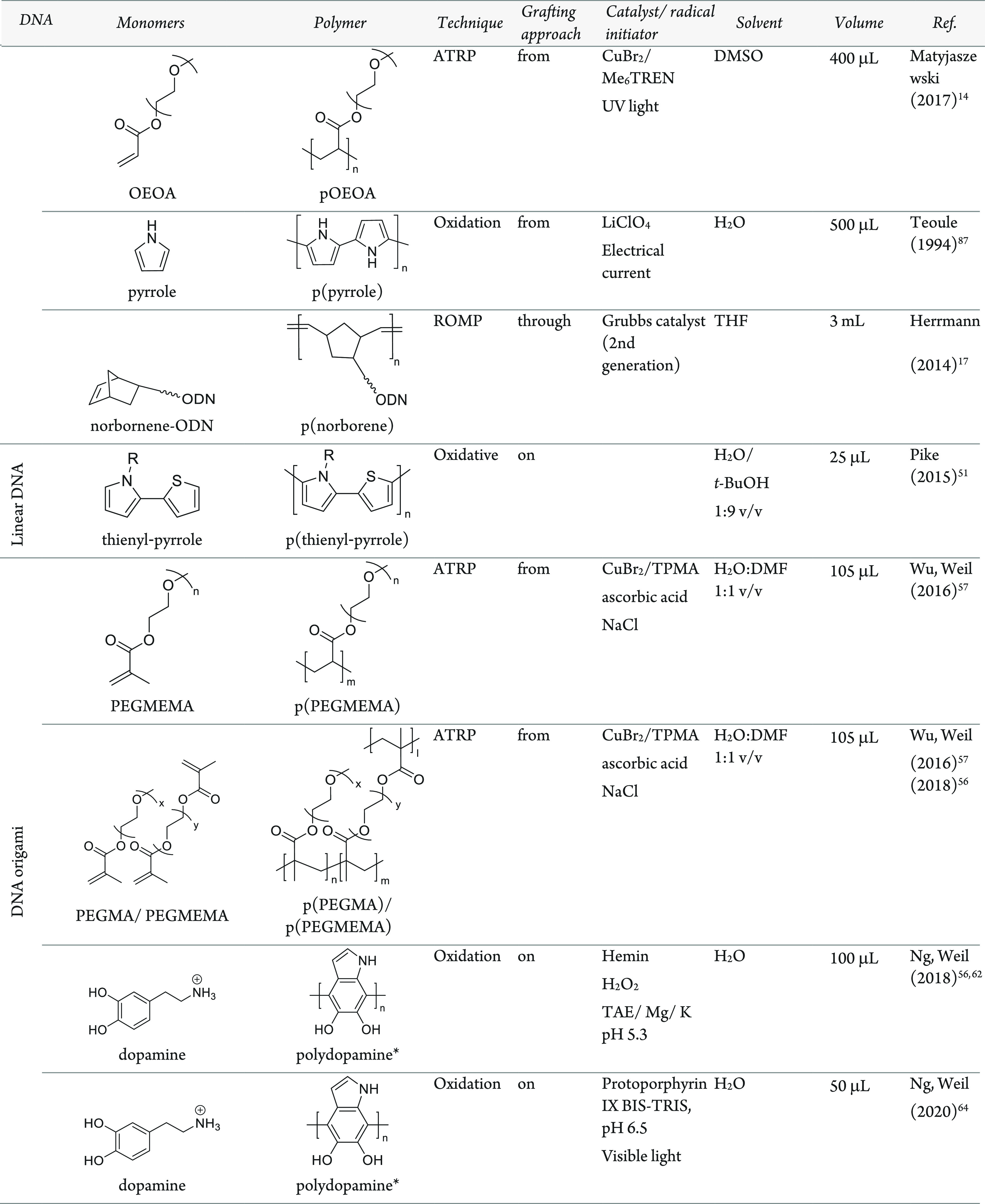
Polymerization Reactions and Conditions in the Presence of DNA^a^

a Polymerizations are performed either *from*, *on*, or *through* DNA regions. DMAm, dimethylacrylamide; OEGMA, oligoethylene glycol methacrylate; DAAm, diacetoneacrylamide; HEMA, hydroxyethyl methacrylate; OEOMA, oligoethylene oxide methacrylate; MA, methyl acrylate; OEOA, oligo ethylene oxide acrylate; PEGMEMA, polyethylene glycol methyl ether methacrylate; TPMA, Tris (2-pyridylmethyl) amine; *, example polydopamine structure.

In addition to the challenges described above, which originate from the physical and chemical environment, the absolute control of the polymerization method is still limited. Nature has the ability to demonstrate sequence defined polymerization exhibiting control and self-assembly in a precise and reproducible manner. These characteristics have inspired attempts to replicate controlled self-assembly with DNA–polymer conjugates. Several developments have been noted by the groups of Liu,^59,78^ Sleiman,^79,80^ and O’Reilly,^81^ establishing bespoke sequence polymerization. However, the intricacy and length of these polymers is still limited. Although there are several challenges when synthesizing DNA–polymer conjugates, several groups have still accomplished many novel and innovative advancements which will be discussed in the following sections.

#### 3.1.3 Solution-Based ODN–Polymer Synthesis

The development of conjugation chemistry has aided the increased variety of polymers conjugated to ODNs. In this section, we will describe conjugation reactions between free ODNs and polymers to synthesize a diblock product with a 1:1 ratio between each block, i.e. conjugations where the ODNs have been cleaved from the solid support prior to polymer conjugation. By performing the ODN cleavage prior to the conjugation reaction, a wider range of chemistries can be performed as deprotection and side reactions are no longer limiting.

One of the most direct methods of DNA–polymer conjugation employs amine-functionalized ODNs and NHS-activated polymers. Stayton and co-workers demonstrated successful coupling of pNIPAM monofunctionalized with an NHS group to a 7-carbon aliphatic amine-ODN.^88^ Due to the poor solubility of pNIPAM at high temperatures, the reaction was performed at 4 °C to avoid precipitation in aqueous environments. Here, the reaction was performed in 10% DMF with borate pH 9.5, although a successful reaction was also noted in 20% DMF with borate pH 8.2.^89^ In 2001, Park and co-workers employed similar chemistry to conjugate NHS-functionalized poly(d,l-lactic-*co*-glycolic acid) (PLGA) to amine-ODNs in solution.^90^ In water, PLGA degrades due to its ester linkage; however, by adopting NHS-PEG, reaction in an aqueous system becomes possible.^71,91,92^ The comparison between these approaches highlights the challenge when conjugating DNA with hydrophobic polymers which may require organic solvents to dissolve. Additionally, the hydrophobic nature of the polymer may cause phase-separation from the ODN. In another example, Park and co-workers conjugated PEG to an ODN by amide coupling. In this instance, an acid cleavable linker was incorporated through an ethylenediamine intermediate attached between the ODN and the tertiary amine group providing a route to DNA release in the acidic environments of cellular compartments.^93^ This demonstrates the potential for dynamic and changeable structures which will be discussed in section 4.2.3. Alternatively, to overcome DNA solubility restrictions for amphiphilic conjugation, Herrmann and co-workers employed a cationic surfactant to stabilize DNA.^17^ In the presence of the surfactant, DNA was soluble in DMF, DMSO, THF, and CHCl_3_ and provided the opportunity for higher yielding conjugation reactions toward hydrophobic polymers, such as PPO, PI, and PS. This approach therefore opens great potential for amphiphilic DNA–polymer conjugate synthesis in solution.

Michael addition reactions have also been explored for ODN–polymer conjugation. Kataoka and co-workers synthesized a conjugate through the thiol–ene Michael addition of thiol-ODN to acrylate-PEG in tris-buffer pH 8.0 (aqueous). In each case, either an acetal^94^ or a lactate^95^ group was present at the opposite end of the polymer to the acrylate group but both did not affect the reaction. A similar conjugation was performed by the same group; however, in this instance, the DNA was replaced with RNA and the thiol group was positioned at the 5′-end in contrast to the 3′ as in the two previous examples. Here, the reaction was carried out with triphenylphosphine in DMF, which was also compatible and produced the desired product.^96^ Through the reactions with acrylamide described here, the incorporation of the acid labile ester group, β-thiopropionate, is consequently situated between the ODN and polymer blocks to enable a pH-responsive complex for RNA release. Herrmann and co-workers chose to perform Michael addition coupling with a maleimide activated PS to thiol-ODN. The maleimide-PS was dissolved in THF and mixed with thiol-ODN to result in a low yield of 13%.^71^

In addition to amine and thiol anchors, azide- and propargyl-ODN can also be exploited through the copper(I)-catalyzed Huisgen [3 + 2] cycloaddition to conjugate free propargyl-DNA to azide-functionalized polymers in solution.^97^ Matyjaszewski and Das employed the polymer poly(oligo(ethylene oxide) methacrylate) (OEOMA), synthesized via ATRP of OEOMA to yield an average molecular weight of 14 700 Da, which was conjugated in high yields to the desired ODNs. Here, ACN was adopted to stabilize Cu(I) in the absence of a ligand while THF was added to dissolve the polymer. Conjugations to PEG have also been demonstrated with moderate yields.^98^ However, to expand the diversity of polymer conjugates, conditions for amphiphilic conjugates are likewise desired. Reaction conditions were investigated by O’Reilly and co-workers for pNIPAM in 100% DMF, with final yields between 70 and 90%.^99^ Hydrophobic polymer conjugation toward DNA was demonstrated using alkyne-modified poly(styrene) (*M*_n_ 4.4). In this case, the click reaction between PS and DNA produced high yields of 74% which had not previously been observed for similar approaches, providing an improved avenue for connecting DNA with hydrophobic polymers. Matyjaszewski and Das also demonstrated this click conjugation reaction with three polymers of similar molecular weight (PEG–methacrylate–pOEOMA_475_, pOEOMA_300_-*co*-MEO_2_MA, and pOEOMA_475_-*co*-DMAEMA) to RNA.^100^ Here, the solvent was reduced to 0.6% ACN/H_2_O and coupling was again successful. The versatility of DNA–polymer conjugate synthesis was demonstrated by the click reaction on both RNA and DNA ODNs with polymers of varying hydrophobicities, which opens opportunities for downstream applications. Additionally, in each example, high yields are reported which exhibit a robust approach for conjugation in solution compared to the thiol–ene Michael addition reaction.

To address the challenge of poor yields often noted for DNA to polymer conjugation reactions in organic solvents, O’Reilly and Wilks conducted a comprehensive investigation of DNA–polymer covalent binding, analyzing amide coupling, thiol–ene Michael addition reactions, and tetrazene-norbornene coupling efficiencies to pNIPAM.^18^ This work was highlighted in section 3.1.1 and will be expanded here to discuss the limitations and possible solutions. Amine coupling to carboxylic acids was attempted with common coupling agents, such as EDCI and DCC with HOBt as the coreagent in a variety of solvents; however, no product was observed. Coupling with hexafluorophosphate benzotriazole tetramethyl uronium (HBTU) and hexafluorophosphate azabenzotriazole tetramethyl uronium (HATU) agents was successful on the first attempt; however, a lack of reproducibility in both cases was noted. The activated esters, PFP esters, and NHS esters were similarly trialed; however, product formation was also not observed. Under the reported conditions, i.e. <10 μM of DNA in 10 μL, it can be concluded that free carboxylic acids as well as activated acid esters are not efficiently coupled to amines. A similar observation was noted in their studies using thiol–ene Michael addition. Methacrylamide, acrylamide, and maleimide functional groups were investigated for the conjugation with thiol groups which, in all cases, did not provide any conversion. Conversely, tetrazine to norbornene coupling appeared most promising with up to 50% yields. The coupling was demonstrated with both tetrazine– and norbornene–DNA to the target polymer, showing the versatility of this approach. The DNA–tetrazine to the pNIPMA–norbornene coupling was, however, the most efficient method, improving yields from 10 to 50% and demonstrating its versatility in organic solvents, i.e. DMF, dimethylacetamide (DMAc), and NMP. In this study, low concentrations and a low volume were adopted which highlighted the limit of these reactions for polymer conjugation to DNA. However, these reactions have been successful by other groups where higher volumes, such as 300 μL,^94^ and higher concentrations, such as 25 μM,^100^ have been adopted. Therefore, where resources are not limited, successful conjugation via conventional coupling methods can be envisaged.

As with each example so far, the polymers are presynthesized separately from the DNA, and therefore, the polymerization reaction itself is not subjected to the limitations of DNA. Additionally, this *grafting to* approach allows the characterization of both the polymer and DNA blocks to understand the composition and properties prior to conjugation. However, conjugation yields are often low due to either solvent incompatibility, repulsion of charged polymers, or also the steric strain as discussed in section 3.1.1. An alternative method using the *grafting from* approach can reduce the impact of steric strain due to consecutive single monomer attachments as well as increase the ability to access shorter polymers blocks due to the ease of purification of the final conjugate. Matyjaszewski and Das conducted the *grafting from* polymerization from DNA in solution and varied the reaction time, catalyst, monomer, and salt concentration.^13^ Here, they polymerized OEOMA and showed that at a high NaCl concentration of 300 mM, no polymer was produced and that without salt, the higher molecular weight polymer was synthesized. Additionally, the lower Cu% (% compared to the monomer) yielded the largest molecular weight along with a 120 min reaction time. Of note, Matyjaszewski and Das employed the activators generated by electron transfer technique (AGET) of ATRP, which has been optimized for aqueous and biologically relevant reaction conditions.^101,102^ This advancement has been demonstrated by several groups and provides new avenues for polymerization by *grafting from* ODNs (Table 3).

**Table 3:**
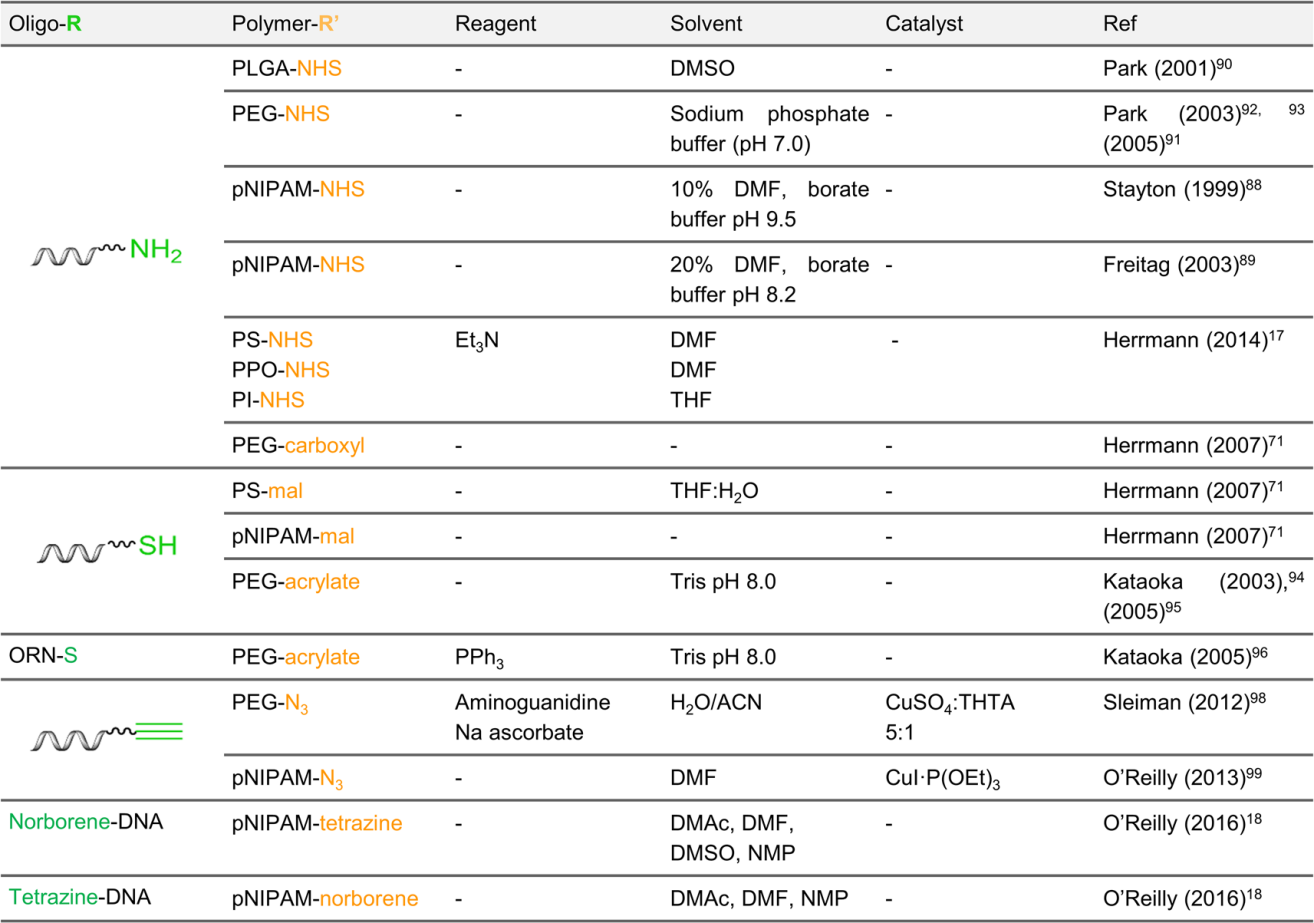
Coupling Chemistries to Covalently Bind ODNs to Polymers

With the increased interest in synthesizing biologically relevant polymers and, therefore, the incorporation of biological material, methods to reduce the chance of downstream toxicity caused by contaminants from the polymerization were desired. These contaminants typically result from common ATRP methods which require a copper-based catalyst, free radical initiators, and reducing agents. However, through photoinduced ATRP (so-called photoATRP), free radical initiators and reducing agents are no longer required and, additionally, the catalyst concentration can be reduced. Several acrylate- and methacrylate-based polymers were synthesized by photoirradiation for 30 min under these mild reaction conditions,^14^ demonstrating an important approach to synthesize hydrophobic polymers. This is a key step in polymer synthesis to broaden the scope for DNA–polymer conjugates. Notably, these polymerizations were fully automated on an adapted DNA synthesizer. The synthesizer was modified to contain a light source in addition to a program that can inject the second monomer to form a diblock copolymer once 100% conversion of the initial monomer has occurred. PhotoATRP is also possible using blue light.^84^ Here, a thorough investigation was performed to determine optimal reagent concentrations for OEOMA_500_ polymerization in aqueous environments. For example, Cu concentrations of at least 100 ppm were required to produce good conversion.^84^ An alternative photoinitiated polymerization from ODNs was demonstrated through photoRAFT using Eosin Y as the photocatalyst.^19^ This polymerization was performed in solution which removes the requirement of a DNA synthesizer. Two RAFT agents were trialed, BTPA and CPADB, to synthesize several polymers, DMA, NIPAM, oligo(ethylene glycol) methyl ether acrylate (OEGA), and oligoethylene glycol methacrylate (OEGMA), demonstrating the versatility of this approach. Additionally, the length of the polymer was controlled by the initiator to monomer ratio—at a ratio of 200:1 of monomer to RAFT agent, polymer length was 13.8 kDa in comparison to 31.2 kDa at a ratio of 500:1. One significant challenge of DNA–polymer conjugates is the incompatibility of hydrophobic monomers or polymers with the hydrophilic DNA. However, with the methodology evolved from PISA, this incompatibility was exploited to direct the formation of different DNA–polymer nanostructures.^73^ This technique was first demonstrated with ODNs by performing the *grafting from* using DMA, 4-acryloylmorpholine, 2-hydroxyethyl acrylate, and OEGA.^74^ Restrictions imposed by the DNA such as ultralow volumes and its associated problems with degassing were circumvented by using glucose oxidase to ensure an oxygen-free environment for the polymerization.^77,85^ By adopting thermal RAFT polymerization and through the inclusion of enzyme degassing, the monomer to initiator ratio can be controlled precisely and thus can allow the manipulation of architectures.^74^ In addition to thermal and photoinduced polymerization methods, ultrasonication is also a possible stimulus.^86^ Through the use of ultrasonication, room temperature and low levels of Cu catalyst can be adopted to yield polymers with low dispersity and high molecular weight.

Other polymerization methods such as those via oxidative approaches have also been investigated to graft polymers from ODNs. The copolymerization of pyrrole monomers present in solution and those conjugated to ODN was performed *in situ* to yield polypyrrole polymers grown from and attached to DNA.^87^ Here, the polymerization is driven electrochemically and in solution to enable high chemical stability. This technique was also performed in the presence of noncomplementary and complementary ODNs demonstrating its capabilities to polymerize from both ss- and ds-ODNs.^103^

Beyond conventional homo and block copolymers, the attachment of sequence defined polymers has made several interesting developments. In particular, sequence-specific polymerization of short polymers was demonstrated by employing a cyclic binding and dissociation of complementary ODNs (propagation strands) bearing the desired monomer for sequential polymer growth.^81,104^ On binding, the complementary strands bring the reactive monomers into close proximity for specific polymerization reactions. A Wittig reaction was employed for simultaneous propagation of the polymer and release of the monomer from its original ODN. To afford multiple cycling steps, the initial duplex exhibits a short noncomplementary region (the toehold domain) to enable a fully complementary displacement strand to remove the propagation strand and leave the ss-ODN bearing the polymer chain. Although the local environment from each reaction step is constant, longer lengths are not possible due to each reaction yield reducing cycled material.

#### 3.1.4 1D DNA–Polymer Synthesis

**Figure 9:**
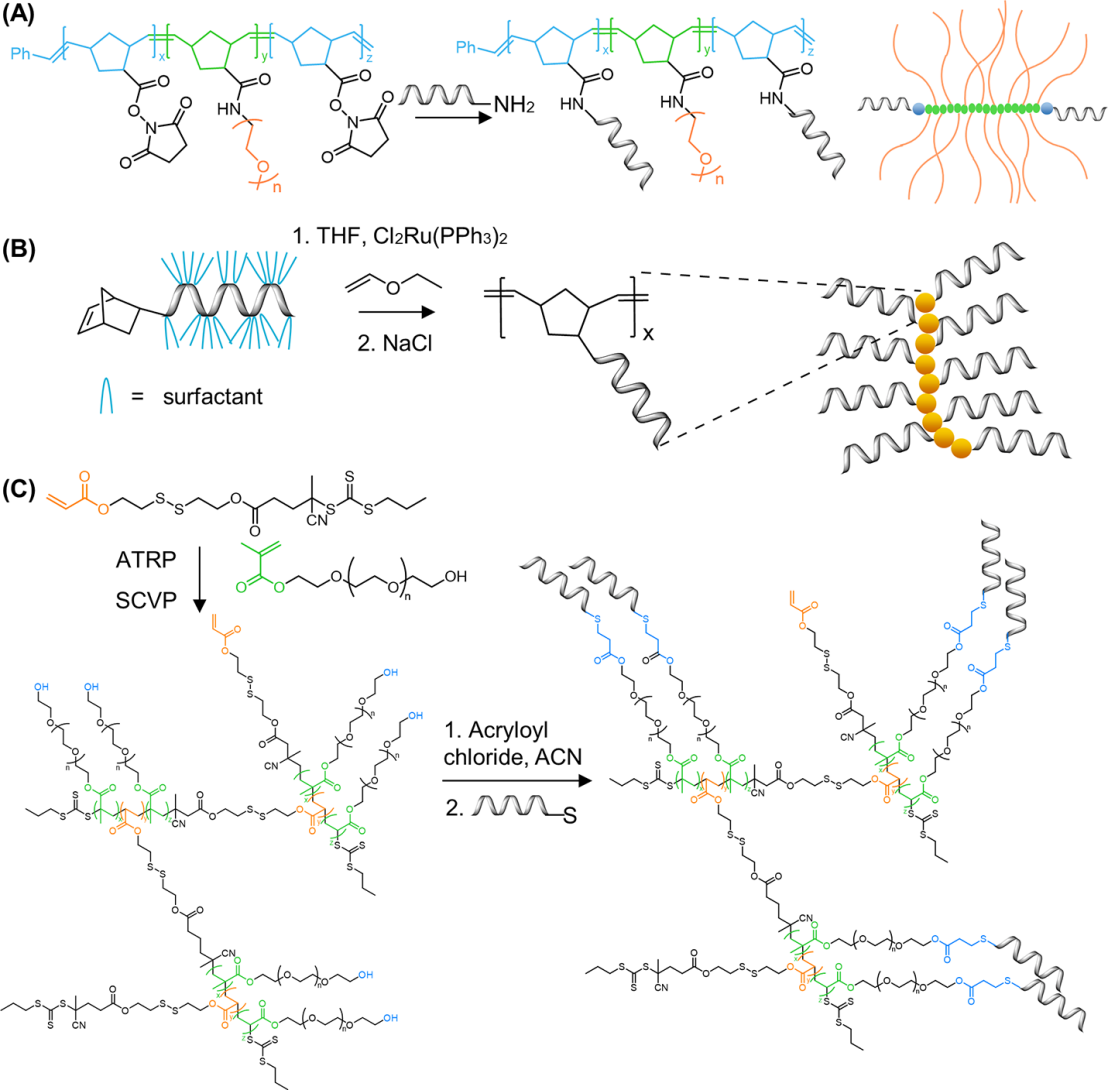
Synthesis of DNA–polymer brushes via *grafting through*. (A) Synthesis of pacDNA through the presynthesis of PEG brush copolymers bearing NHS anchors via a *grafting through* ROMP followed by amide coupling of amine-ODN. The chemical structure of the polymer brush backbone and a 2D schematic are shown as product representatives.^55,113^ Based on figures from refs (55 and 113). (B) DNA side-chain brush polymers synthesized from the norbornene-ODN monomer. Several polymer length scales were synthesized, represented here as a multimer.^17^ Based on figures from ref (17). (C) Dual polymerization employing ATRP and SCVP to produce a diblock copolymer consisting of PEG side chains.^119^ Based on figures from ref (119).

In this section, the development of 1D structures, such as DNA–polymer brushes will be outlined. The most common method described for DNA–polymer brush synthesis is the ROMP of norbornyl bound to polymer side chains and reactive handles, which can be employed for DNA attachment. Zhang and co-workers applied this approach to synthesize DNA–PEG conjugates consisting of PEG_5000_ and PEG_10000_ side chains.^105^ In their design, branched PEG structures, named pacDNA (polymer assisted compaction DNA), were synthesized via the consequential ROMP of norbornyl-NHS (N-NHS) and norbornyl-PEG (N-PEG), for diblock synthesis, and through chain extension ROMP with N-NHS for triblock copolymer synthesis (Figure 9A).^55,106−108^ The NHS anchors along the backbone were then available to couple amine-ODNs.^109^ To further exploit the potential of DNA–polymer conjugates, Zhang and co-workers polymerized norbornyl-paclitaxel (an anticancer drug), again via ROMP prior to DNA conjugation to produce spherical nucleic acids—macromolecular structures to be discussed in section 4.1.1.^110^ A similar approach was adopted to synthesize DNA–polymer conjugates where doxorubicin (DOX) was also covalently bound within the structure.^111^ In each case, a diblock copolymer was synthesized by ROMP of norbornyl-DOX and N-PEG, where the carboxyl groups at PEG terminals were activated with EDC and NHS for 5 min prior to amide-coupling with amine-ODN. These two examples demonstrate the potential of DNA–polymer conjugates as drug delivery systems and their ability for high capacity drug loading. Further details of applications will be discussed in sections 5.2 and 5.3. In an example by Mirkin and co-workers, a copolymer consisting of a polycaprolactone (PCL) and PEO block where only the PEO backbone was functionalized with an azide group was synthesized and enabled copper-free click chemistry with a DBCO-ODN for conjugation.^112^ In this instance, the copper-free click chemistry was performed in a 1:1 DMSO/DMF mixture in the absence of an aqueous buffer. The examples described above demonstrate the employment of diblock copolymer structures synthesized prior to DNA conjugation. However, through the employment of a triblock copolymer, where each block initially consists of non-DNA content, controlled positioning of the ODNs along the polymer backbone can be realized. This was accomplished through a triblock copolymer brush synthesized by the sequential ROMP of N-NHS, followed by N-PEG, and again N-NHS with Grubbs catalyst.^113^ Again, the NHS groups were then available to couple amine-ODNs at the terminal polymer blocks of the brush structure. A further development in the design of triblock copolymers enabled the synthesis of an N-NHS-N-PEG-norbornyl maleimide (N-MI) triblock and demonstrated a dual-ODN conjugation approach through the orthogonal reactions of amine-ODN to N-NHS and thiol-ODN to N-MI.^113^ This development enabled the incorporation of two distinct and specific ODNs within one nanostructure to open possibilities for dual-functionalization.

In an alternative approach to ROMP, Liu and Li employed the ROP of γ-propargyl-l-glutamate *N*-carboxyanhydride^114^ to synthesize a polypeptide capable of click chemistry between azide-functionalized ODNs and the propargyl group after the polymerization was complete.^115^ This technique enabled the synthesis of a hybrid peptide DNA brush, containing functional possibilities that have high biomedical relevance due to its biocompatibility and postfunctionalization potential. Additionally, it provides a platform for high loading of DNA to target drug delivery entities: 5–6 ssDNA molecules could be conjugated to one polypeptide.

In each case described above, the ODN has been conjugated to the polymer as a postpolymerization strategy. An alternative approach is to incorporate the ODN *in situ* through initial monomer conjugation and proceed with a *grafting through* type polymerization. This was demonstrated by the attachment of norbornyl to the ODN followed by ROMP (Figure 9B).^17^ Depending on the ODN length adopted (either a 7- or 14-mer), polymers of short lengths (a tetramer, pentamer, hexamer, and heptamer for the 7-mer and a dimer, trimer, and tetramer for the 14-mer) could be synthesized and purified by polyacrylamide gel electrophoresis (PAGE). Of note, the ROMP here produced the longest polymer products in 100% THF. As described in section 3.1.1, the employment of cationic surfactants is crucial to enable the solubility of DNA in organic solvents for solution-based reactions. Through this approach, postpolymerization functionalization is not required and therefore reduces reaction steps as well as reducing cross-reaction complications. Additionally, using the *grafting through* strategy ensures that every monomer unit contains an ODN whereas a postpolymerization reaction is subjected to a statistically dispersed functionalization of the side chains. This approach has been similarly demonstrated with a peptide nucleic acid (PNA) where the PNA unit was covalently attached to a norbornyl group and subsequently polymerized via ROMP.^116^

RAFT polymerization can also be employed to synthesize 1D DNA–polymer conjugates. Martyjaszewski, Armitage, and Das adopted the RAFT polymerization of methacrylate groups bearing macroinitiator side chains which can then be polymerized to yield a “bottlebrush” polymer.^117^ In this instance, each “bristle” contained an azide group to afford click chemistry with an ODN.^117^

A similar approach to *grafting through*, dual polymerization, can produce highly branched polymer structures. One example combined self-condensing vinyl polymerization (SCVP) and cation ROP to produce a poly(3-ethyl-3-oxetanemethanol)-PEO hyperbranched multiarm copolymer.^118^ An alternative approach replaced cation ROP with RAFT polymerization to synthesize a hyperbranched polymer structure again bearing PEG side chains but with a RAFT agent core containing a disulfide bond for redox-responsive drug delivery (Figure 9C).^119^ This method allows the synthesis of both hydrophobic and hydrophilic blocks simultaneously. In both cases described, ODNs were conjugated to the branched polymers by the Michael addition of thiol-ODN to acrylate-functionalized branched polymers through a final *grafting to* step. A hyperbranched polymer network was also demonstrated by the laboratories of Sumerlin and Tan.^120^ SCVP was employed to copolymerize *O*-nitrobenzyl acrylate, PEG-acrylate, and 2-(2-bromoisobutyryloxy) ethyl acrylate which also served as the inimer to instigate branching. Once synthesized, a two-step substitution was performed to transform the chain end hydroxy groups to azide reactive groups for click chemistry anchors. Copper free click chemistry was then performed utilizing a strained alkyne DBCO-modified DNA to afford high yielding conjugation in 100% DMSO. Through these methods, hyperbranched structures can be readily synthesized and postfunctionalized with the ODN for the required drug loading.

#### 3.1.5 2D and 3D Polymerization Platforms

**Figure 10:**
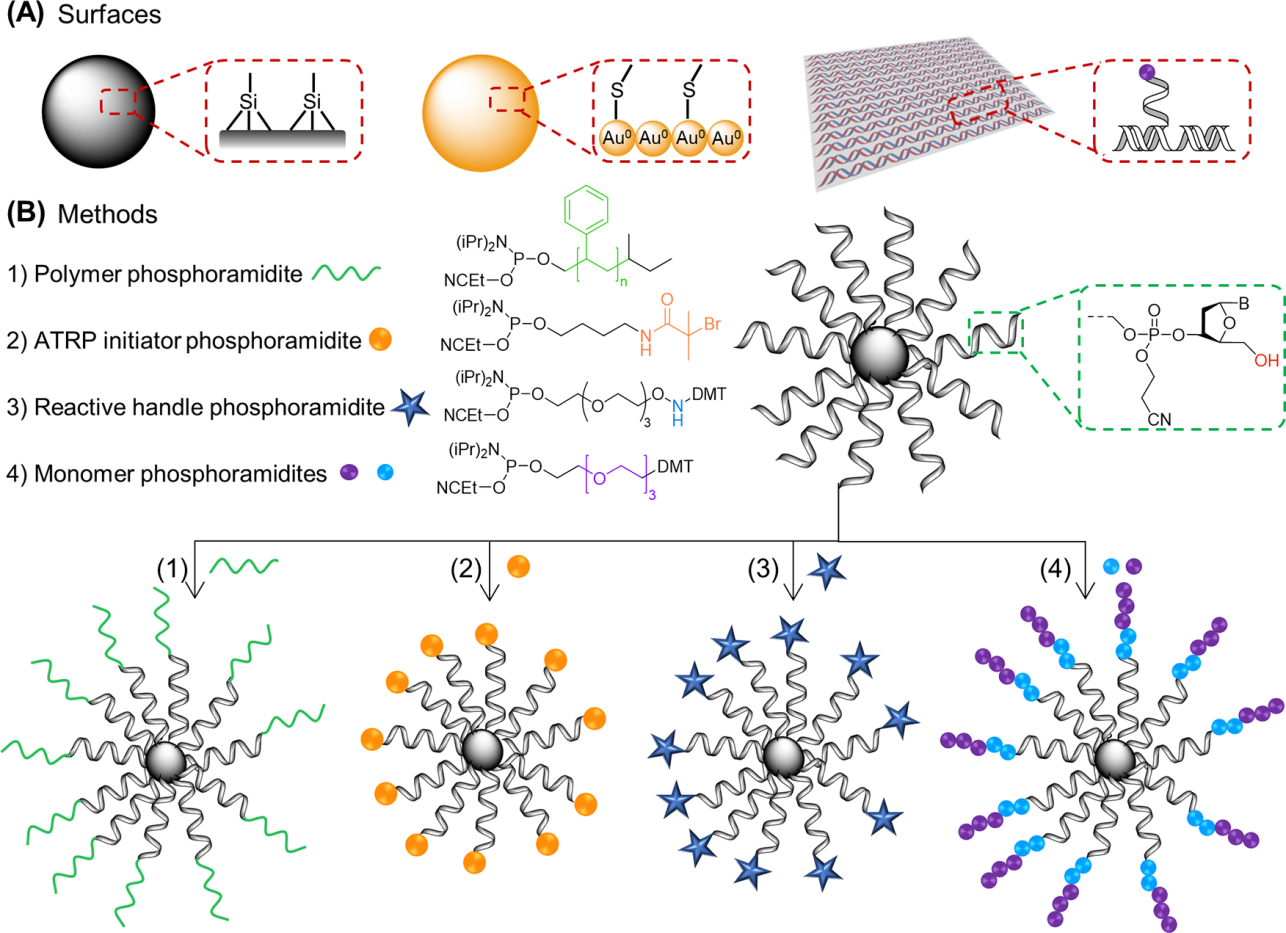
(A) Example surfaces for DNA–polymer conjugation synthesis. Surfaces include whole macro materials in addition to precisely defined nanostructures. (B) Methods for CPG bead surface DNA–polymer synthesis through either the attachment of a (1) polymer phosphoramidite,^121^ (2) ATRP initiator phorphoramidite,^13^ (3) reactive handle phosphoramidite,^9^ and (4) monomer phorphoramidite.^80^

The above-mentioned methods each perform the conjugation reaction in solution. In contrast, solid supports and surfaces can also be adopted to synthesize DNA–polymer conjugates through both *grafting to* and *grafting from* methods. Platforms include nanomaterials, such as DNA origami, nanoparticles, and beads which together encompass metal, organic, and biological surfaces consisting of varying 2D and 3D structures and properties (Figure 10A). Several first attempts to conjugate DNA and polymers were performed using solid phase synthesis. In 2004, Mirkin and colleagues synthesized a PS phosphoramidite via solid phase synthesis,^121^ demonstrating the ability to synthesize a complex phosphoramidite and carry out an efficient coupling step in the presence of the protecting groups. This technique was further explored by Hermann and co-workers to synthesize PPO conjugates employing polymer phosphoramidites.^122,123^ The polymer-phosphoramidites were synthesized by the reaction of alcohol terminated polymers with chlorophosphoramidite with yields of 41% and 32% for PPO polymers of 1000 and 6800 g/mol, respectively.^122^ These polymers can then be conjugated to the ODN through standard phosphoramidite chemistry. In addition to PS and PPO, a cholesterol-TEG phorphoramidite was also synthesized to subsequently yield a cholesterol-ODN.^124^ The employment of beads can also grant the selection of shorter polymer brush structures due to the pore size,^98^ which can sometimes be a limitation. However, conjugation employing polymer phosphoramidites on solid supports requires a DNA synthesizer which is a dedicated instrument that requires specialized skills to use. Some commercial companies can offer the delivery of ODNs still attached to the solid support; however, this may not always be possible. Although the reported yields in the above examples are lower than the optimized reactions in solution, by employing phosphoramidite chemistry, the conjugation occurs while nucleotide functional groups are protected and thus conjugation using a solid support offers a more diverse compatibility with coupling reagents and functional groups in comparison to solution-based reactions.

An alternative approach is to synthesize the ODN and polymer with complementary reactive click chemistry handles. Zhang and co-workers synthesized several copolymers using azide-polymers with alkyne-functionalized ODNs on the CPG beads. Polymers consisted of poly(*tert*-butyl acrylate) (PtBA) and PS of molecular weights 3.9, 5.5, 8.5, and 14 kDa.^125^ The yield was determined for each conjugation to three ODN lengths (6-, 19-, and 26-mer) and revealed an increase in yield with decreasing lengths of both polymer and ODN.^125^ This study highlights the limitation of steric hindrance on efficient conjugation where two flexible polymers are required to come into close contact for the reaction to occur. Conjugations with the lowest molecular weight PtBA and PS with the 26-mer produced similar product yields to example click reactions performed in solution (between 70 and 90%);^99^ thus, either method can be adopted. However, on increasing the polymer length to higher molecular weights, the yield decreased; for example, the yield was 56% for the conjugation between the 14 kDa PS and the 26-mer on the CPG bead. Therefore, there is a trade-off between polymer and ODN length and product yield.

A combination of the solid phase synthesis approach with the presynthesized polymer brush was adopted by Gianneschi and co-workers to produce a polymer brush with multiple ODNs attached to solid supports, followed by the deprotection and cleavage steps.^126^ The polymer brush was synthesized via ROMP of a benzene-norbornyl followed by norbornyl-N-acetyloxy-succinimide. After polymerization, the acetyl group was available for conjugation to the amine-ODN with DIPEA and HBTU to activate the carboxylic acid. Conjugations with ODNs capable of forming defined secondary structures (aptamers) were similarly performed.^127^ However, alterations to relative equivalents were noted, 3× higher DIPEA and 4× less HBTU in comparison to the previous solid support reaction, demonstrating that optimization is required for different reacting partners. Gianneschi and co-workers also synthesized an RNA–polymer conjugated following the same procedure for the aptamer DNA,^128^ demonstrating the robustness of this approach. Inspired from the development of conjugating polymers from the surface of CPG, presynthesized polymer nanoparticles, which bear chemical handles, became a natural expansion of the technology. A copolymer consisting of MA and azide-modified MMA units self-assembled into a nanoparticle, exhibiting the azide on the surface.^129^ Cu-free click reaction is then possible with DBCO-ODNs to yield 3D DNA–polymer nanoparticles.

Similar to the *grafting to* approach, *grafting from* has been demonstrated on solid supports. Matyjaszewski and Das explored polymerization from initiators bound to ODNs through both solid phase and solution phase ATRP by performing the polymerization either pre- or post-CPG bead cleavage.^13^ Performing the polymerization on the solid support provides easier purification from the unreacted monomers and catalyst; however, the initiator phosphoramidite must be compatible with deprotection and cleavage reactions. After the polymerization of OEOMA for 4 h, a molecular weight of 205 kDa was noted after cleavage from the CPG beads. On solid support, it is challenging to accurately quantify the concentration of initiators and thus the initiator/monomer ratios. Conversely, in solution, concentrations can be determined and, therefore, optimizations involving reagent ratios can be performed more accurately.

Solid phase synthesis *grafting from* the ODN was also performed on a gold surface through the complementary binding of a thiol-modified ODN (attached to the surface) to an ATRP initiator-modified ODN.^130^ This approach produced poly(hydroxyethyl methacrylate) (PHEMA) from a gold surface, only when the complementary initiator sequence was bound. Further exploration employed a dual-functionalized ODN bearing a thiol group at the 3′-end and an ATRP initiator at the 5′-end and demonstrated ATRP growth from a purely ssDNA sequence on the gold surface.^131^ Here, faster growth kinetics were observed when the initiator was present on the ssDNA rather than directly on the surface. This effect can be noted due to the localization of the Cu catalysts on DNA, increasing proximity to the initiator site. Several RAFT conditions were also investigated to optimize pOEGMA and PHEMA synthesis via RAFT polymerization. Temperature, time, CTA% surface density, and AIBN concentration were each explored and showed a temperature of 30 to 40 °C (although higher temperatures were not investigated) is required for polymerization to occur, a reduction in growth as AIBN concentration increases from 0.4 mM, as well as a linear growth trend on increasing time for pOEGMA.^83^ Through the employment of a conductive 2D surface, the development of chips for applications requiring sensitive surfaces can be envisaged. To further establish the *grafting from* approach on a solid support, He and co-workers adopted a gold nanoparticle employing the thiol–gold interaction.^82^ ATRP was performed through the initial coupling of an ATRP initiator, bromoisobutyryl, to the thiol-ODN, followed by Au nanoparticle (NP) attachment and incubation in polymerization reagents. Here, pOEGMA was synthesized from the ODN-modified AuNP, and through the presence of DNA, these reactions could be performed in an aqueous environment without aggregation or solvent exchange requirements. In each case described here, surface attachment is utilized; thus, characterization of the polymer is limited and requires cleavage prior to analysis.

In the examples described so far in this section, the polymer sequence is either a repetitive monomer or a copolymer of random arrangement. Nature consists of several polymers, such as DNA and proteins, which contain a complex but controlled sequence of monomer units. These biological and precise polymers inspired the group of Sleiman to develop a method for sequence defined synthetic polymers.^80^ Through the employment of phosphoramidite chemistry, the sequential addition of defined monomers via stepwise coupling and washing was realized. In this instance, two phosphoramidite oligomers consisting of either a hexaethylene glycol or hexaethylene (HE) unit were adopted as hydrophilic and hydrophobic monomers and ordered in a controlled manor (Figure 10B). In their first work, polymer lengths of up to 12 units were explored and shown to have high control over the sequence. They next explored polymer lengths up to 24 units with varying content demonstrating the ability to increase polymer length through this approach.^79^ This method highlighted the potential for DNA–polymer conjugation where both the DNA and polymer content are sequence defined, although polymer length is still limited and may require further exploration to improve the solid phase monomer conjugation scope.

**Figure 11:**
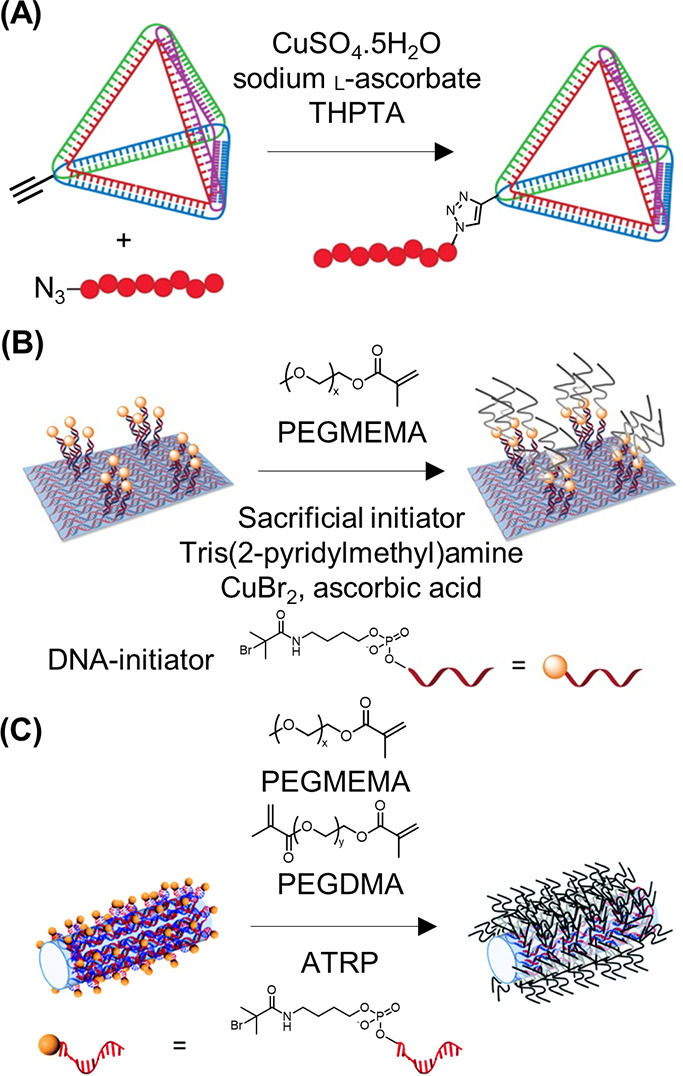
2D and 3D DNA polymer conjugates on “solid” DNA nanostructures. (A) *Grafting to* DNA tetrahedron through click chemistry of pNIPAM to the alkynyl-DNA nanostructure.^99^ Adapted with permission from ref (99). Copyright 2013 American Chemical Society. (B) *Grafting from* DNA origami tiles through Cu-catalyzed ATRP. Initiators are initially bound to the origami structure through complementary sticky sequences followed by the ATRP reaction.^57^ Adapted with permission from ref (57). Copyright 2016 John Wiley and Sons. (C) *Grafting from* a DNA origami tube through ODN bound initiators.^56^ Adapted with permission from ref (56). Copyright 2018 the Royal Society of Chemistry.

The examples so far have produced a conjugate containing DNA as a polymer block; however, they have not exploited the capabilities of DNA to guide the polymerization to precise assemblies through its sequence-specific interactions. DNA sequences can be programmed through their specific base pairing to form folded nanostructures. DNA nanostructures were first envisaged by Seeman in 1980, beginning as lattice structures up to more recent examples of sophisticated DNA origami structures as reviewed previously.^132^ DNA origami was proposed by Rothemund^133^ and has provided a powerful approach to engineer nanoscale functional structures.^134,135^ Structural DNA nanotechnology was first employed in covalent conjugation with polymers by O’Reilly’s group, demonstrating the use of a DNA tetrahedron as a structural anchor for polymer attachment.^99^ The polymer-decorated DNA nanostructure was realized through Cu-catalyzed click chemistry between the alkyne bearing DNA tetrahedron and azido-functionalized pNIPAM (Figure 11A). A 100-fold decrease in reagent concentrations was stipulated in comparison to their ODN equivalent solution-based click reactions. Additionally, CuSO_4_/tris(hydroxypropyltriazolylmethyl)amine (THPTA) was adopted rather than CuI·P(OEt)_3_. However, as with the attempts using polymer brushes, steric effects are deterministic on the efficiency and can result in low coupling yields. By *grafting from* the nanostructure, steric restraints are reduced for the monomer polymerization processes. The added advantage of employing a 3D DNA nanostructure backbone afforded polymer patterning through the site specific attachment.

In the previous example, the functionalized DNA strand was part of the folded nanostructure. However, in another instance, site specific control was employed through complementary base pairing an ODN bearing the radical initiators.^57^ The origami was designed to exhibit “sticky” ssDNA at precise locations to guide the initiator-ODN to the predesignated sites (Figure 11B). To perform the polymerizations from DNA origami, ATRP was employed to achieve reactions in aqueous conditions and at room temperature—a requirement when handling DNA origami. Due to the low concentrations of DNA origami available, sacrificial ATRP initiators were required in solution to maintain the radical equilibrium. Under these conditions, polymerization of PEGMA successfully generated a polymer brush. By adopting copolymerization with the cross-linker PEG dimethacrylate (PEGDMA), a dense polymer network can also be created. The DNA origami tile structure can also fold to form a tube shape, converting the 2D patterned surface to a 3D dual-surface containing specific internal and external contours (Figure 11C).^56^ Here, ATRP initiators were similarly placed in precise patterns to decorate the outer surface of the tube. The polymerization conditions remained unchanged between the tile and tube configuration, therefore demonstrating the versatility of ATRP on DNA origami for nanoscale precision of DNA–polymer hybrid nanostructures.

### 3.2 Noncovalent DNA–Polymer Interactions

Apart from the covalent DNA–polymer conjugates reviewed above, there is also the emerging class of supramolecular assemblies of DNA and polymers which is driven by noncovalent interactions. Here, intermolecular communication is enabled through the close proximity and attraction of both materials, which lead to systems of dynamic nature. While covalent conjugation requires chemical manipulation of the DNA strands to equip them with reactive handles, noncovalent approaches do not face these constraints and can typically be realized with nonmodified and readily available nucleic acids. The highly programmable primary structure of DNA as well as the ability to shape secondary and tertiary structures can be exploited to control the sequence of polymers that are structurally unrelated to nucleic acids. This strategy takes inspiration from one of the essential processes found in living nature, where the DNA-encoded information on life is replicated, transcribed, and translated through RNA into proteins. Noncovalent assemblies can be classified by their mode of interaction as well as by the designated purpose. DNA provides multifaceted interaction modes that arise from its unique structure in all three dimensions as discussed in section 2. ssDNA is accessible via Watson–Crick base pairing whereas the negatively charged phosphate backbone is prone to electrostatic interaction with polycations. dsDNA expands the toolkit by enabling hydrophobic groove binding and intercalation of planar molecules into the stacking bases along the DNA backbone. In comparison to covalent conjugation strategies, the herein described noncovalent interactions are *per se* not specific, yet several studies aim to circumvent these intrinsic restrictions and seek for spatially controlled attachments. Since DNA is a multifaceted platform, one can utilize its exceptional customizability to design tailor-made polymers by either templating or patterning approaches. On the one hand, ssDNA and dsDNA allow sequence transfer onto growing polymers and the templating of supramolecular 1D and 2D structures, respectively. On the other hand, DNA can be arranged in complex nanostructures which can be covered with polymers, rendering new features to the synthetic building block and yielding three-dimensional constructs. Furthermore, the extraordinary fidelity of certain DNA arrays, e.g., DNA origami, permits the patterning of polymers in distinct shapes and with a precision that outcompetes other techniques, such as lithography or conventional self-assembly. However, independent of the applied technique, the DNA template can be either removed after polymer synthesis or become part of the reaction product. In line with the focus of this review, we survey promising strategies to develop DNA–polymer conjugates via noncovalent interactions.

#### 3.2.1 Templating of Polymers by Single and Double Stranded DNA

**Figure 12:**
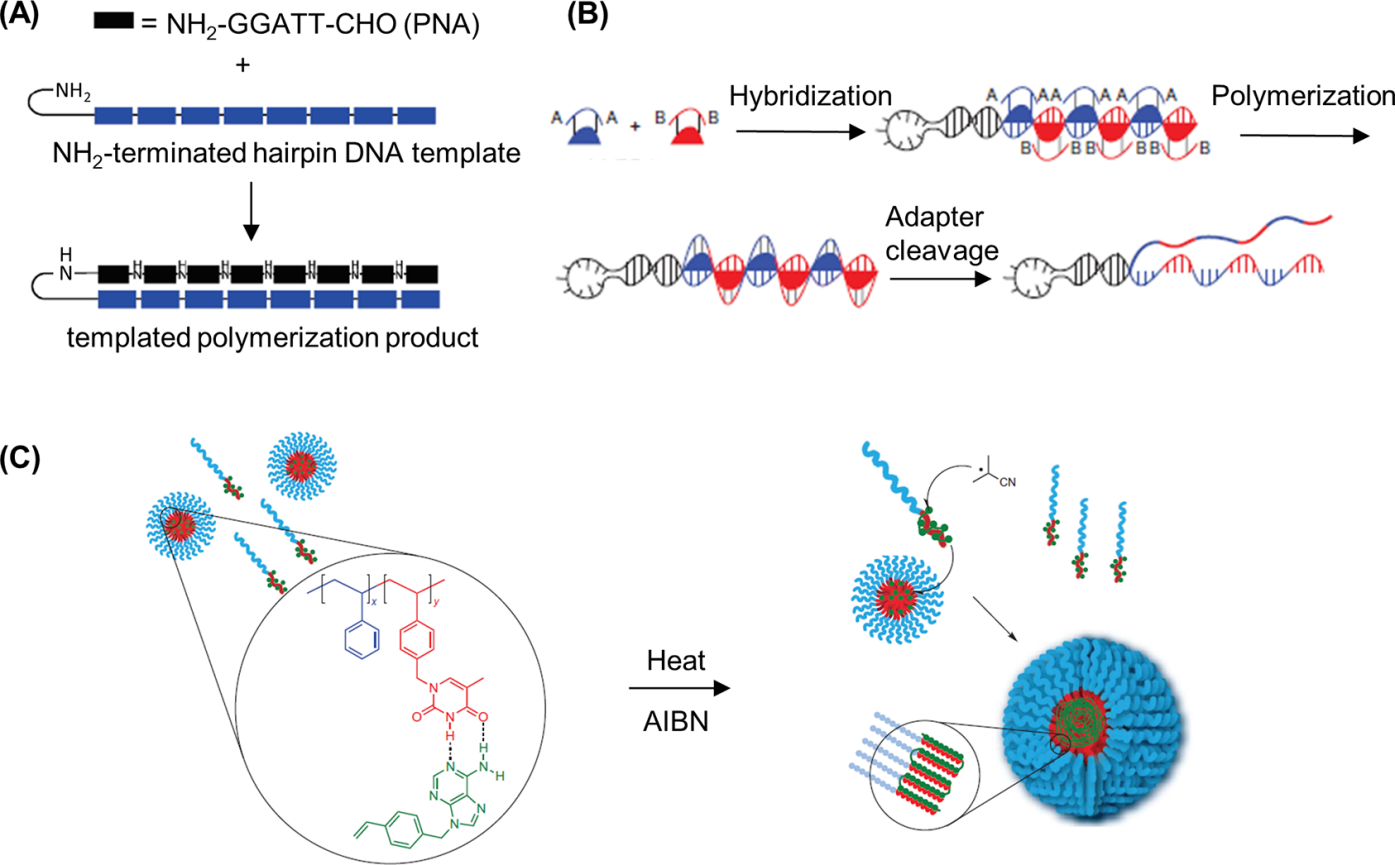
Various strategies to sequence-controlled polymer growth with ssDNA. (A) DNA-templated polymerization of PNA pentamer aldehydes on an amine-terminated hairpin DNA template.^78^ Reproduced with permission from ref (78). Copyright 2008 American Chemical Society. (B) Codon-mediated linkage of AA/BB-substrates yields polymers that can be released from the templating DNA by cleavage of the disulfide linkers.^59^ Reproduced with permission from ref (59). Copyright 2013 Springer Nature. (C) The combination of segregation and templating techniques ensures confined chain growth along a template in discrete micelle cores that affords polymers of high molecular weights.^138^ Reproduced with permission from ref (138). Copyright 2012 Springer Nature.

One of the greatest advantages of DNA over synthetic polymers is the unprecedented level of sequence-control as well as the consequential precision in molecular weight and distribution. It therefore is attractive to exploit this unique characteristic and potentially transfer the molecular information onto polymers. In this way, ODNs can function as molecular matrices that recognize and interact with guest molecules and, thus, organize them according to their sequence and guide subsequent polymerization. An early example of how the sequence of nucleic acids might be harnessed was demonstrated by Liu and co-workers^136^ wherein short PNA sequences were arranged in a sequence-specific fashion along an amine-end-modified ODN template via complementary base pairing. Aldehyde moieties on the tetrameric PNA monomers allowed for distance-dependent reductive amination coupling, ligating the monomeric units, which consequently generated polymers with molecular weights of 10 kDa. Introduction of mismatches afforded no or only truncated polymers, depending on the position at which the error was placed. Furthermore, the presence of additional building blocks with closely related sequences did not disturb the formation of the desired product. Thus, efficient and sequence-specific conjugation of nucleic acid templates and non-natural polymers steered by hydrogen bonding of base pairs could be established. In a follow-up study, the group expanded their monomer scope through a side-chain-functionalized PNA tetramer and pentamer aldehydes^78^ (Figure 12A). Thereby, they could fabricate densely functionalized polymers, involving a PNA 40-mer with more than half the nucleotides bearing side chains. Interestingly, the polymerization efficiency mainly depended on the position and stereochemistry of the side chains rather than on size, hydrophobicity, or charge. As briefly mentioned above, Nature has the capability to translate the genetic information stored in nucleic acids into amino acid-based peptides and proteins. Based on their previous work, Liu’s group aimed to mimic the last step of Nature’s protein machinery where the sequence of a nucleic acid template allows a codon-mediated conversion into an amino acid sequence and, in the end, a protein is released (Figure 12B).^59^ These codons bear a template recognition site as well as the corresponding amino acid. In order to exploit this strategy and likewise introduce sequence-specificity to non-natural polymers, the group designed a codon comprising a PNA pentamer for template recognition and a synthetic polymer building block. Through the employment of PEG as the initial polymer model and by achieving molecular weights of up to 10 kDa, they could further incorporate α-(d)- and β-peptide backbones with various side-chain functionalities to accomplish longer and structurally more diverse polymers (26 kDa). Among several investigated conjugation strategies, copper-catalyzed alkyne–azide cycloaddition of AA-/BB-substrates proved to be most efficient. Moreover, by equipping the codon with a disulfide bridge between the polymer building block and the PNA adapter as a cleavable linker, the polymeric product could be liberated afterward. Though the approach described here utilizes Watson–Crick base pairing to produce sequence-controlled polymers without the need for Nature’s enzymatic toolbox, the structural diversity of the substrates is still limited to macrocycles for entropic reasons. However, the codon design theoretically supports the incorporation of several building blocks without the need to readjust the template recognition site.

Another seminal approach to adopt from Nature’s capability to make exact copies of nucleic acid strands was investigated by the Sleiman group.^137^ Step-growth polymerization techniques typically suffer from poor control over molecular weight which inevitably leads to broad molecular weight distributions. They therefore employed nucleobase recognition to surpass these barriers and to synthesize conducting polymers of low dispersities. Instead of using pure ODNs as a template, a thymine-decorated polymer was manufactured via living ring-opening metathesis polymerization (ROMP). Alignment of adenine-containing monomers by complementary base pairing along the template strand and subsequent Sonogashira coupling afforded well-defined daughter strands of conducting polymers. Whereas nontemplated polymerization or polymerization with an incorrect template only produced low polymerization degrees and high dispersities (PDI > 2), the presence of the correct template significantly narrowed the molecular weight distribution (PDI = 1.2) and yielded similar polymerization degrees compared to the parent strands. Thus, the all-synthetic strategy proved to be capable of programming the structure, length, and dispersity of commonly poorly-defined polymers by hydrogen bonding interactions. Following this, O’Reilly and co-workers furthered nucleobase-promoted polymer templating by combining the methodology with a segregation strategy that makes use of block copolymer self-assembly (Figure 12C).^138^ Their bioinspired dual templating/segregation approach relies on the isolation of propagating radicals in discrete micelle cores, thus enabling confined chain growth along a template. Briefly, a block copolymer of styrene (St) and the thymine analogue 1-(vinylbenzyl)thymine (VBT), PSt_115_-*b*-PVBT_18_, was synthesized that forms stable micelles in chloroform. The hydrogen bond interaction of the thymine template with a vinyl derivative of adenine (VBA) ensured the solubility of the adenine monomer in the solvent. Furthermore, the addition of the complementary adenine monomer led to a dynamic exchange of adenine-loaded templates into the micelle where the ensuing polymerization was taking place. A so-called “hopping” mechanism of propagating radicals along adjacent templates in the micelle core can theoretically explain the remarkably high molecular weights of the daughter polymers of up to 400 kDa, even though the template only counts 18 thymine residues. Hence, nucleobase templating enriched the free radical polymerization to yield narrowly distributed daughter strands (PDI ≤ 1.08) by suppressing bimolecular termination in a confined environment.

The examples discussed so far mainly report on nucleobase-modified polymers or closely related structures such as peptide nucleic acids. To further develop the field, Zhou et al. studied the triplex hybridization of a polymer with a full carbon backbone alongside DNA and RNA ODNs to produce conjugates that might be suitable for DNA loading onto nanoparticles or delivery of siRNA in biomedical applications.^139^ Here, RAFT polymerization of various acrylates yielded polyacrylates with tunable side chains. As a key step, these copolymers were equipped with triaminotriazine (so-called melamine) handles by amidation of NHS moieties along the backbone. Melamine can recognize thymine and uracil hydrogen bonding patterns in various media and, therefore, ensured the hybridization with ODNs comprising two blocks of the respective amino acid that are bridged by a cytosine linker (dT_10_C_10_T_10_). Notably, the RAFT copolymers are intrinsically characterized by stereoregio backbone heterogeneity and still engage T/U rich ODNs with nanomolar affinity upon mixing in a 1:1 ratio. The supposed triplex hairpin binding model of the compounds was further affirmed by FRET studies.

**Figure 13:**
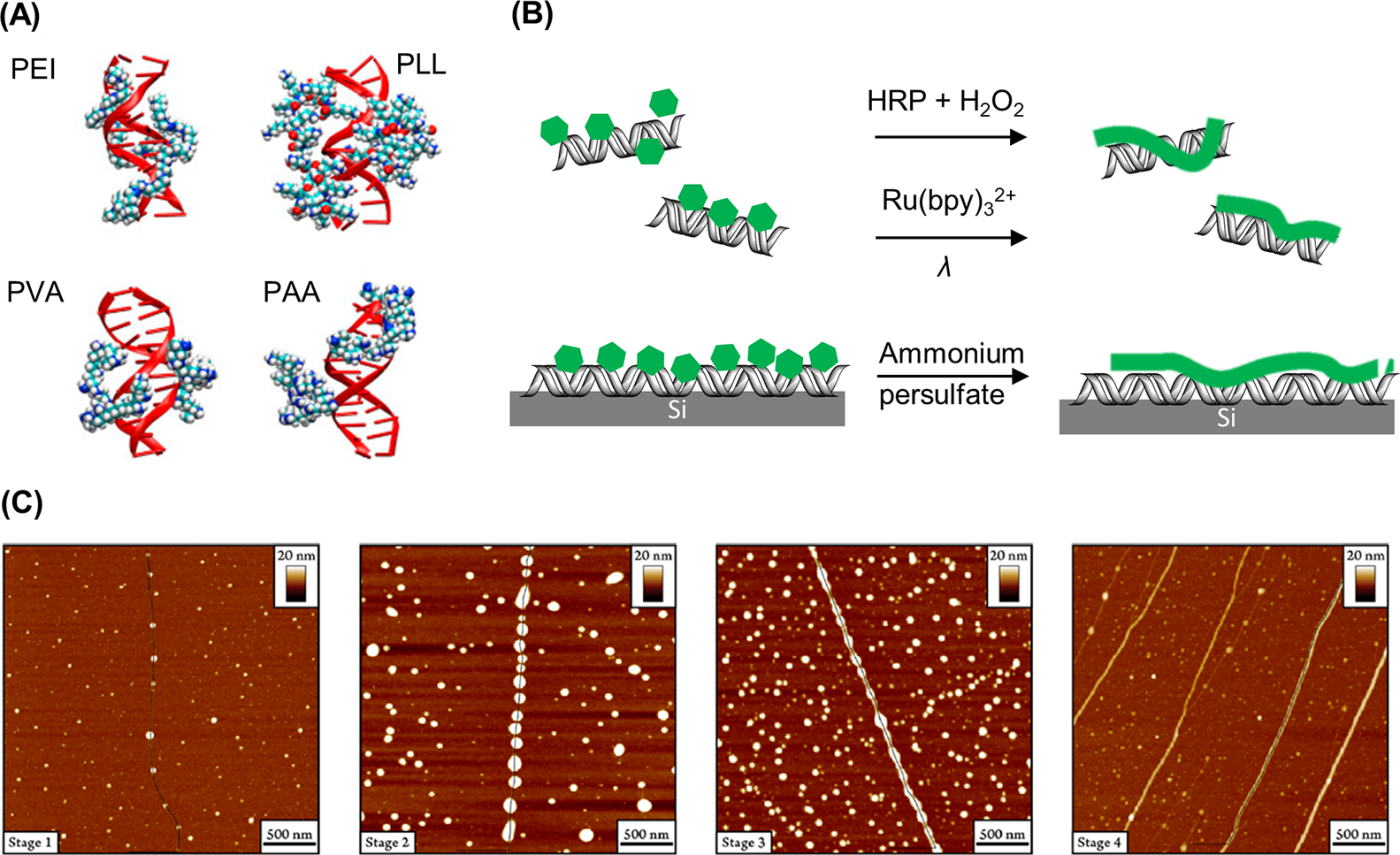
Electrostatic interactions allow the alignment of positively charged monomers and polymers along the DNA backbone. (A) Complexes of DNA with two polycation chains for DNA–PEI, DNA–PLL, DNA–PVA, and DNA–PAA systems.^141^ Reproduced with permission from ref (141). Copyright 2018 Elsevier Ltd. (B) The electrostatic alignment of aline-monomers on DNA, either in solution or on Si substrates, and subsequent oxidation leads to the formation of poly(aniline) structures along the DNA template. Oxidation can be induced enzymatically (HRP), via photo-oxidation of Ru complexes or by using oxidants (APS). (C) AFM images of the polymerization process could reveal four distinct stages in the formation and growth of poly(pyrrole).^149^ Reproduced with permission from ref (149). Copyright 2014 American Chemical Society.

The work described here represents a rare example of DNA–polymer conjugates that are solely based on hydrogen bonding between a fully synthetic polymer and complementary nucleic acids without the support of electrostatic interaction. However, substantially more studies address the negatively charged phosphate backbone of DNA with respective polyplexes due to the convenient and spontaneous mode of interaction. For instance, electrostatic complexation can be exploited to condense siRNA onto a positively charged supramolecular polymer for drug delivery purposes.^140^ The cationic polymer can enter cellular membranes via charged-mediated endocytosis and successfully deliver its cargo, thus inducing gene silencing. Supramolecular complexes of nucleic acids with cationic polymers have emerged prominently in the area of gene delivery in order to circumvent viral delivery vectors. In this respect, several positively charged polymers such as polyethylenimine (PEI), poly l-lysine (PLL), polyvinylamine (PVA), and polyallylamine (PAA) are subjects of current research (Figure 13A).^141^

Electrostatic interactions also play a key role in the studies of the Herrmann group where they fabricated light harvesting DNA complexes and described the salt-free hybridization of PEGylated ODNs in water.^142,143^ In a two-step process, a water-soluble surfactant is employed to transfer the DNA into an organic phase where it is substituted by an amine-containing molecule, for instance, amine-PEG. Hereby, ODNs can be noncovalently encapsulated with a PEG shell that allows for the formation of metal-free dsDNA with remarkably high thermostability.

Likewise, electrostatic interactions can be further utilized to template polymerization along DNA, enabling these interactions to dictate bond formation processes. There are a high number of studies demonstrating the use of DNA templates to exert control over the respective sequence and structure as an appealing strategy in the field of conducting nanowires. In particular, polyaniline, polypyrrole, and polythiophene are well investigated.^144^ Polyaniline (PANI) is commonly synthesized in a strongly acidic environment through chemically or electrochemically induced oxidation of aniline monomers. However, these harsh conditions prevent the use of biological templates as they are highly sensitive materials. Oxidative polymerization of aniline therefore necessitates the adjustment of reaction conditions toward mild pH ranges and tolerable oxidation agents (Figure 13B). In an initial attempt, Simmel and co-workers employed three different stimuli to trigger the polymerization of aniline along a λ-DNA template in solution as well as on a chip surface.^145^ Prior to polymerization, DNA and monomers were simply incubated in phosphate buffer at pH 4.3, without the need for any chemical modification. Positively charged anilinium ions act as counterions for the negatively charged phosphate backbone and are organized accordingly. Enzyme-mediated oxidation through horseradish peroxidase (HRP) and hydrogen peroxide, photo-oxidation using a ruthenium complex, and ammonium persulfate as an oxidant all proved to be capable of yielding PANI-decorated DNA conjugates. In a similar approach, the group of He aimed to fabricate conducting polyaniline nanowires along preoriented DNA templates which were aligned on a Si substrate.^146^ Oxidation of aniline was also induced enzymatically by HRP and hydrogen peroxide. Adjusting the pH value to pH 4.0 turned out to be crucial with regard to wire quality: at a pH of 5, the continuous formation of wires was interrupted by polyaniline particles; however, lowering the pH to 3.2 yielded only incomplete polymerization. Thus, the optimal pH range to ensure continuous and regular polymerization was on the one hand determined by the optimized electrostatic alignment of aniline monomers along the template and on the other hand helped to retain sufficient enzyme activity. By incorporating AuNPs into polyaniline nanowires derived from DNA templates, Wang et al. showed the novel construction of hybrid nanowires with expanded electrical properties.^147^ Therefore, a sequential assembly process was applied: positively charged AuNPs were aligned on surface-immobilized DNA templates, affording narrow AuNP chains. The gaps between neighboring particles were then bridged by Ru-mediated photopolymerization of aniline derivatives in acidic media. The alternating AuNP–polyaniline hybrid nanowire could then be visualized by atomic force microscopy (AFM).

As with many other aromatic heterocycles, pyrrole can also be polymerized in oxidative environments. Thus, double helical DNA permits the construction of 1D nanostructures through the organization of the pyrrole precursors and subsequent oxidation and polymerization. In order to generate pure polypyrrole–DNA conjugates with alkynyl side groups, Horrocks and co-workers implemented chemical modifications into a thienyl-pyrrole monomer (TP).^148^ It could be shown that monomer functionalization had no negative impact on the oxidative polymerization that was mediated through ferric chloride (FeCl_3_). Treatment of the conjugates with Tollen’s reagent led to the binding of silver cations to alkynyl residues which facilitated nucleation and growth of Ag clusters along the backbone. Compared to unmodified poly(thienyl-pyrrole), many small nanocrystals are formed closely to each other, attaining uniform distribution and enhanced conductive properties. In an ensuing study by Hannant et al., the same monomer was employed to further investigate click chemistry for postmodifications which might be of interest for sensing applications.^150^ Importantly, the pentynyl-substituted pyrrole derived nanowires retained structural integrity and remained active, i.e., conductive, after addition of azido molecules via the succeeding click reaction. To broaden the monomer scope and to demonstrate the generality of the electrostatically driven templating approach, Houlton and co-workers polymerized dithienyl pyrrole monomers (TPT) along DNA templates.^151^ Though this monomer only comprises 1/3 of the number of hydrogen donor sites compared to pyrrole and therefore reduced bonding capabilities, successful DNA recognition and interaction was still possible. The higher structural regularity of the polymer justifies the use of a less active monomer, since simply mixing thiophene and pyrrole monomers only yields randomly alternating sequences.

Investigation of the growth mechanism of pyrrole monomers along DNA templates by AFM imaging unveiled a stepwise polymerization process. First, low densities of conducting polymer bind to DNA as apparently spherical particles, followed by denser particle packing in a beads-on-a-string fashion, which then resulted in subsequent dynamic reconfiguration, finally elongating and merging the particles in highly regular nanowires with smooth morphology (Figure 13C).^149^

The controllable interplay of not only electrostatic but also hydrophobic interactions between DNA and polymers opens up a completely different possibility to define the morphology of resulting conjugates. Once more, nature was used as a role model with respect to its outstanding ability to store genomic DNA with the help of histones. Chen and co-workers tread new pathways for the noncovalent interaction of block copolymers and DNA by establishing a two-step self-assembly process.^152^ Notably, the micelle formation of amphiphiles is not only determined by their concentration (critical micelle concentration, CMC) but also significantly relies on the ratio of water phase to organic phase, which is known as the critical water content (CWC). Based on the latter phenomenon, the group designed a self-assembling system of polymers and DNA which is first guided by weak electrostatic interactions that are subsequently caught up by hydrophobic driving forces. They therefore utilized a copolymer that comprises two blocks, a hydrophilic PEG and a hydrophobic poly(4-vinylpyridine) (P4VP). Below the CWC for micellization, the positively charged P4VP interacts with DNA, forming linear complexes in which the DNA is encapsulated by the polymer. Gradual increase of the water content allows for hydrophobic aggregation of the P4VP blocks between polymer chains in solution and polymer chains on the DNA. The hydrophobic interaction then forces rearrangement of the complex and finally leads to core–shell nanofibers in which DNA wraps around the hydrophobic polymer aggregate. When employing monodisperse and relatively short DNA templates, these properties were transferred into the DNA–polymer conjugates which are monodisperse in both length and width. The necessity of the DNA template is clearly evident since the copolymer alone only accumulated in spherical micelles under identical conditions.

Besides the binding modes discussed here, the Watson–Crick base pairing and resultant double helix structure further render DNA attractive for the intercalation of planar molecules. In duplex DNA, the environment of nucleobases leads to π–π-stacking of adjacent aromatic systems, a structural motif that has a greater impact on helix stability than hydrogen bonds of complementary bases. Compounds that recognize DNA via interaction within the stacking bases are therefore potential handles for attaching or growing polymers along the DNA template. Hence, respective initiators, monomers, or the *a priori* synthesized polymer have to be equipped with suitable intercalators. Although ethidium bromide is a very strong DNA binder, utilization of weaker intercalating molecules such as acridine can add the potential for reversibility to the complex. O’Reilly and co-workers employed RAFT polymerization to synthesize a series of acridine end-terminated polymers, including pNIPAM and pDMAm and investigated the effect of polymer structure on the nature and strength of the interaction with DNA.^40^ Indeed, differences in complexation behavior were observed, which were potentially caused by the relative tendencies of the different polymers to self-assemble when brought into close proximity. For instance, a high load of pNIPAM onto calf thymus DNA and full occupancy of intercalation sites induced irreversible aggregation. The DNA-guided vicinity of polymer chains quasi-imitates the process when hydrogen bonds between the amide groups of pNIPAM are formed, normally giving rise to its temperature-responsive character. On the other hand, the compact structure of pDMAm tolerated higher densities of polymer intercalation without aggregation occurring. Thus, the combination of pDMAm and a significantly shorter and well-defined DNA sequence (63 base pairs) yielded discrete and possibly brush-like nanoparticles with sizes of 10 nm. Importantly, DNA or polymer alone as well as acridine-lacking polymer does not form comparable assemblies. In a different approach, Pike and co-workers instead used monomers with π-stacking anchoring groups to arrange the monomers within the DNA helix and conducted polymerization after intercalation.^51^ Based on the intercalation of diazido derivatives of proflavine into the double helix, the azido groups exposed themselves into the major grooves of the DNA. Here, copper-catalyzed click reaction with thienyl-pyrrole monomers was performed. Crucially, proflavine intercalation was not hampered by the ensuing click reaction of the functional groups nor was intercalation of a presynthesized unit of intercalator and monomer successful, due to hydrophobic and steric impediments. Polymerization of the spatially organized pyrrole units was initiated by residual oxygen species in the solvent, without the need for a chemical oxidant.

While this strategy relies both on intercalation within the stacking nucleobases and on chemical reactions taking place in the major groove of DNA, DNA grooves alone also provide the opportunity for noncovalent attachment of polymers. Furthermore, the impact of adjacent base pairs on the groove environment adds a certain level of sequence-specificity to the system which is less prominent among intercalators. To ensure an ideal interaction, groove binding compounds typically comprise at least two aromatic rings while still being flexible in contrast to rigid polycyclic planar molecules that are suitable for intercalation. Deiana et al. investigated the binding mode of an anthracenyl polymer with dsDNA as well as the binding strength and mechanism.^153^ The polymer was synthesized by ATRP from an anthracene macroinitiator with 4 initiator sites, and DNA interaction was induced by simple mixing of the compounds. Association constants in the 10^5^ M^–1^ range are higher than those found for intercalating molecules or electrostatic interactions, thus indicating successful groove binding. Furthermore, the association stoichiometry was ascertained to be 1 polymer-adduct for every 5 base pairs, showing that most sites of DNA participate in the association process. Although groove binding is mainly attributed to hydrophobic forces, van der Waals forces and hydrogen bonding may also be involved in the process, promoted by the hydrophilic polymeric arms.

As already emphasized in the previous section (3.1), the synthesis of covalent amphiphilic conjugates of ODNs and polymers is problematic, especially due to solvent incompatibility, low conjugation yields, and phase-separation. Host–guest interactions can potentially alleviate some of these concerns by constructing a special hydrophobic molecular environment to compensate for the difference between hydrophilic and hydrophobic components in solution. This environment exists as hydrophobic cavities within a general hydrophilic exterior thus allowing the encapsulation of molecules that would otherwise phase-separate. Varghese and co-workers exploited this interaction mode by equipping DNA with a prominent host molecule (β-cyclodextrin) which can trap adamantine-modified hydrophobic guests.^154^ Supramolecular chemistry widely explores this class of host–guests due to their efficient and highly specific molecular recognition, low price, and simplistic modification. The spontaneously formed self-assemblies from the generated DNA-amphiphiles were found to be thermally stable which is attributed to extremely strong hydrophobic interactions. However, this technique is predominantly exploited to attach small molecules or oligomers rather than polymers.^155^ At this point it is important to note that this observation applies to almost all approaches relying on noncovalent interactions. A rare example for a polymer-based strategy is reported by Thelu et al. in a follow-up study to their work described above.^156^ Herein, adamantyl-terminated 8-arm PEG polymer is encapsulated by X- or Y-shaped DNA carrying β-cyclodextrin at the end of all ODN arms. The combination of a multivalent host with a star-like guest led to nanogel formation, and the gelation was concentration dependent. Moreover, the applicability of these nanoparticles in a biomedical context was accomplished by demonstrating successful drug loading, good cell permeability, and delivery into cells. Thus, due to the universal and modular nature of the host–guest interaction, the approach holds the potential to be further developed.

#### 3.2.2 Polymer Decoration of DNA Nanostructures

While ss- and dsDNA are extensively leveraged to tailor the polymer sequence and nanostructure and to provide integrative functions, polymers themselves can also benefit DNA in several ways. Higher ordered DNA arrays such as DNA origami are exceedingly versatile building platforms, but they face intrinsic stability drawbacks. Due to their nature, DNA objects are prone to enzymatic degradation through nucleases when encountering physiological environments, thus limiting their progress in biomedicine. Even though degradation might be delayed by packing DNA structures of high density or by having multiple interstrand crossings, it cannot be completely impeded.^157,158^ In addition, DNA origami’s integrity is highly dependent on sufficient levels of divalent cations that allow close proximity of DNA strands by compensating charge repulsion of the phosphate backbones. Hence, several studies seek to diminish DNA susceptibility through polymer-based approaches. Despite the drastically reduced accessibility of 2D and 3D DNA objects in contrast to nonfolded DNA sequences, electrostatic interaction can still be exploited in order to achieve an efficient polymer coating. Divalent cations are hereby substituted by polymeric polycations, either naturally derived or of artificial origin. However, without the possibility of confining the electrostatic interactions in a designated area, coverage will occur nonspecifically which might hamper additional postmodifications.

**Figure 14:**
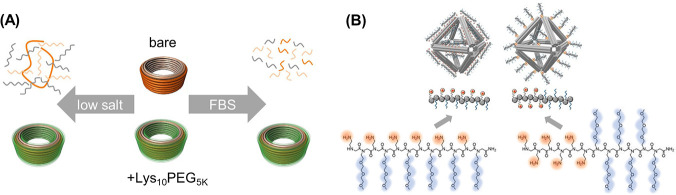
Intrinsic instability of DNA nanostructures under low salt conditions or in the presence of nucleases and fetal bovine serum (FBS) is addressed by many groups. (A) Shih and co-workers could increase the stability of DNA origami by electrostatic coating with PEG–polylysine copolymers.^61^ Reproduced with permission from ref (61). Copyright 2017 Springer Nature. (B) Block- and brush-type copolymers of PEG and peptide mimetics (so-called peptoids) were found to also reduce the susceptibility of DNA nanostructures.^161^ Reproduced with permission from ref (161). Copyright 2020 National Academy of Sciences.

Various polymers were investigated to determine their structure-binding relationship and their impact on origami stability. For instance, ATRP-generated block copolymers of PEG (to improve biocompatibility and protection efficiency) and methacrylate derivatives (to serve as cationic blocks) were attached to a 60-helix bundled DNA structure.^60^ Notably, for successful binding, the ratio of amines within the polymer to phosphate groups within the DNA backbone (N/P ratio) was found to be pivotal. The number of cationic blocks of the copolymer; however, only had a minor impact on binding affinities. Ahmadi et al. followed suit and studied the effect of polymerization degree, charge density, and N/P ratio of linear PEI and chitosan on coating efficiency.^159^ In line with other studies, the N/P ratio significantly determines the extent of interaction with DNA. However, LPEI performed better than chitosan in protecting DNA from salt-depletion and nucleases, even at lower ratios which the authors attributed to its higher charge density. Moreover, the study reveals how challenging the characterization of polymer-decorated DNA nanostructures might be. While bare origamis could be imaged by negative stain TEM, LPEI-coated origamis were only visible after removing the polymer shell. The indirect proof of polymer coverage described here is often the only way to analyze the conjugate (more examples to follow within this section). In 2017, electrostatic coating of DNA origami structures by copolymers from PEG- and polylysine-building blocks were reported by two different groups.^61,160^ The polylysine block interacts with DNA while the corresponding PEG block builds a protective layer and shields the sensitive DNA backbone. The Schmidt lab synthesized PEG_12 kDa_PLys_18_ by ring-opening polymerization induced by an amino-terminated PEG macroinitiator while the Shih group utilized commercially available PEG_5 kDa_PLys_10_ polymers (Figure 14A). Both coatings proved to enable DNA nanostructures to withstand low salt and high nuclease conditions. However, it is important to note that the coating produced with the in-house synthesized copolymer did not allow for further surface functionalization due to the shielding effect by the polymers. Schmidt and co-workers circumvented these restrictions by applying the shorter polymer as it was utilized in the Shih lab, and thus, modifications using AuNP were no longer hampered by the polyplex formation. An important consideration for an electrostatic-based polymer coating is its suitability in a physiological context where charged interactions are subjected to the influences of complex fluids. Interestingly, Shih and co-workers could not only reach a 1000-fold increased stability under cell culture conditions but also confirm the integrity of protected DNA origamis after cell uptake. Remarkably, these results were achieved with dendritic cells that are known for highly efficient DNA degradation, which therefore represent a challenging scenario for the DNA nanostructures. Recently, Gang and co-workers expanded the polymer scope for electrostatic protection by presenting peptoids as valuable candidates.^161^ (Figure 14B) Peptoids are peptide mimetics in which side chains are anchored to the nitrogen atom of the backbone instead of the α-carbon, so that secondary structures and proteolysis are suppressed. Positively charged motifs and PEG monomers were used to construct block-type and brush-type copolymers via solid phase synthesis. The latter architectures were advantageous in stabilizing DNA in biomimetic fluids due to multivalent interactions along the backbone.

**Figure 15:**
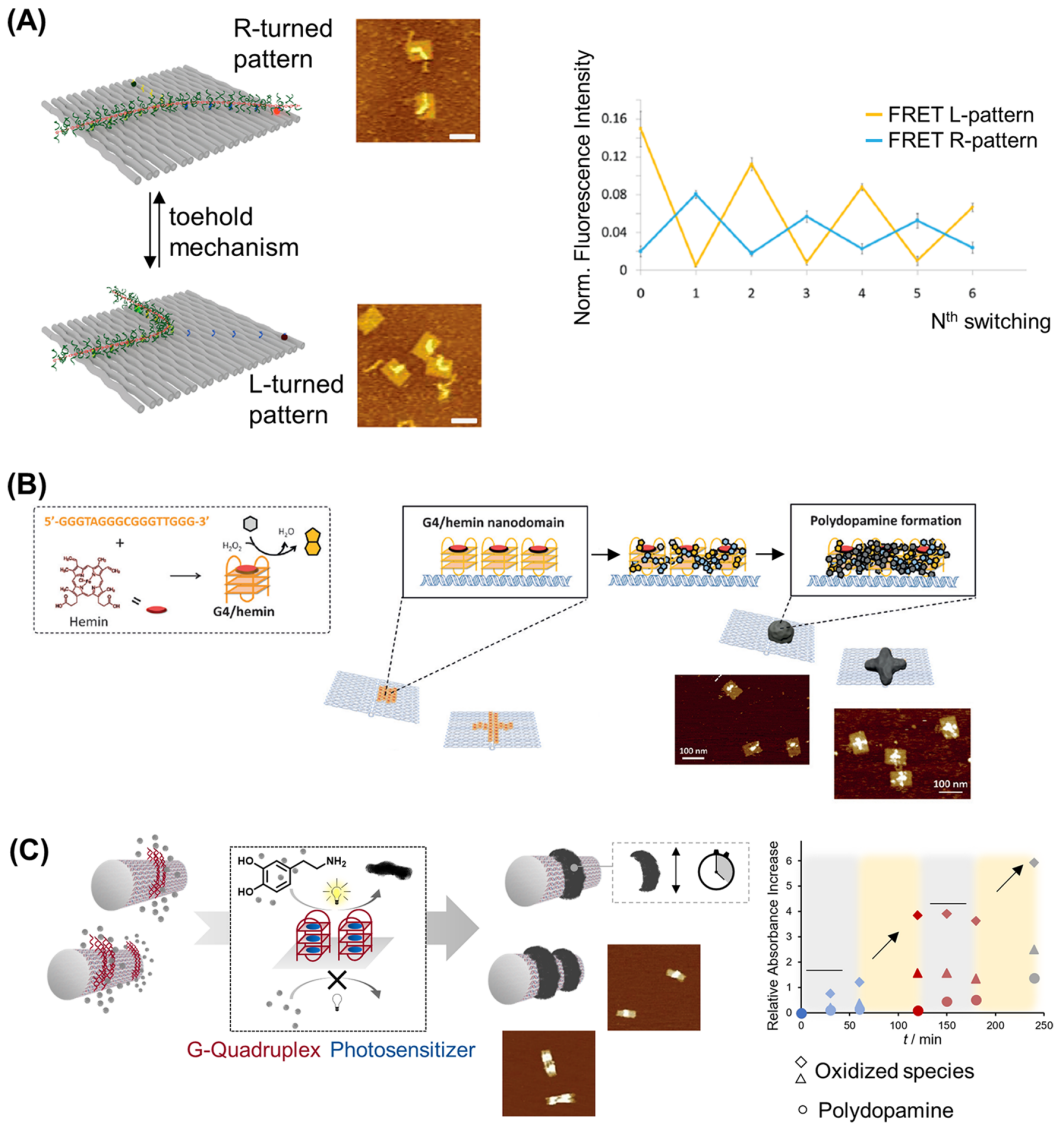
By exploiting the unique addressability of DNA origami, polymer patterning on the nanoscale can be realized. (A) The Gothelf lab developed various strategies to synthesize sophisticated polymers that can be routed on DNA origami platforms on a single-chain level. Furthermore, repetitive switching of the polymer chains was achieved. Switching of the polymer conformation could be monitored by FRET pairs.^54^ Reproduced with permission from ref (54). Copyright 2016 American Chemical Society. (B) The spatially controlled formation of polydopamine in designated patterns was demonstrated by the Weil group by installing enzyme mimicking reaction centers on DNA origami that guided the polymer growth.^62^ Reproduced with permission from ref (62). Copyright 2018 John Wiley and Sons. (C) Further temporal control over polymer formation was implemented by trapping a photosensitizer at distinct positions on 3D origami tubes.^64^ Reproduced with permission from ref (64). Copyright 2020 John Wiley and Sons.

In summary, electrostatic polymer coatings provide appealing simplicity when aiming for increased stability of DNA nanostructures. Additionally, polyamines are even more competent in their stabilizing effect than commonly utilized magnesium ions during origami synthesis.^162^ Nevertheless, the polymer–DNA conjugates derived are of rather low specificity, which is adequate in only addressing stability issues. For more sophisticated objectives, polymer deposition with spatial and temporal control is more ideal. Additionally, unspecific coating inadvertently wraps reactive handles within the polymer shell thus jeopardizing the key aspect of DNA origami structures. In order to conserve the fidelity of DNA origami, some researchers designed individual strategies to orchestrate polymer alignment. Among the spectrum of suitable noncovalent interactions, complementary base pairing can enable highly precise attachment of polymers, even on a single polymer chain level. Therefore, it is necessary to furnish both DNA origami and polymers prior to conjugation: complementary ODNs have to be mounted on the DNA origami scaffold at designated positions as well as on the polymer chains, guiding the interaction of both building blocks. Gothelf and co-workers developed a method to equip several side groups of a polymer with ODN handles allowing a polymer to interact with a DNA origami template on multiple sites, not only via, e.g., end-group modification. Thus, the alignment of single polymer chains along a predestined path on DNA origami was envisioned.^163^ In detail, solid phase DNA synthesis was employed to graft ODNs from several side chains of a poly(phenylenevinylene) polymer while additional PEG side chains ensured water solubility of the construct which is required for successful DNA conjugation later on. The sophisticated nature of the polymer led to a rather broad size distribution as characterized by GPC (340–3300 kDa) and AFM (length range of 20 to 200 nm), presumably due to partial degradation during purification. However, binding yields of polymers to various DNA origami tiles were still very high according to AFM images. It should be noted that more complex alignments, for instance, a staircase path instead of a U-shape, lower the assembly efficiency. While the visualization of these 2D origami structures was accessible by AFM, the same analysis was highly difficult for 3D objects. 3D characterization was only possible when applying the DNA PAINT technique, again highlighting the challenges in investigating polymer–DNA origami conjugates. In a follow-up study, the Gothelf lab further developed their strategy by installing a programmable switch within the polymer configuration on DNA origami (Figure 15A).^54^ Since complementary base pairing is always of a reversible nature, a so-called toehold mechanism can be applied to trap and release the ODN-modified polymers through different anchors on the DNA origami platform, thus guiding the polymer along various routes. Despite the elaborated efforts, the approach is hampered by synthetic and analytical issues, arising from the very low dimensions on the nanometer scale. Notably, only half of the origami structures exhibited well-aligned polymers, demonstrating challenges during conjugation. Furthermore, repeated switching of the polymer conformation and subsequent AFM imaging was not possible due to very strong background noise resulting from added displacement strands. Subsequently, each cycle of conformation switch was performed in solution, which enabled purification after each step. Moreover, simultaneous alignment of two different conductive polymers and the ensuing interpolymer energy transfer was not successful, indicating the limits for conjugation of intricate polymers.^164^

Despite the rather low number of reports on distinct polymer patterning on DNA origami, there are some studies that do not involve base pairing but electrostatic interactions. In contrast to the studies discussed above, here, electrostatic coating is restricted to only occur within distinct boundaries. This is enabled by pursuing a fundamentally different strategy compared to the aforementioned interactions of polycations with the DNA origami. Here, polymers are grown directly from the DNA surface while simultaneously forming stabilizing electrostatic interactions. As mentioned earlier, the aniline monomer responds to oxidative polymerization as it can be activated by oxidants, photoreactive metal complexes, or enzymes, such as horseradish peroxidase (HRP). Ding and co-workers took inspiration from the latter mechanism and established a HRP-mimicking system to polymerize aniline on DNA.^165^ Therefore, they equipped a DNA template with guanine-rich sequences that are known to build so-called G-quadruplexes, representing DNAzymes. Upon incorporation of hemin as a cofactor and the addition of hydrogen peroxide, oxidation of aniline is induced. Due to electrostatic interactions, generated aniline radicals adhere to the negatively charged phosphate backbone in close proximity, thus attaining local polyaniline formation close to the DNAzymes. The group then transferred the regioselective polymer growth to 2D DNA origami triangles.^63^ They could demonstrate that polyaniline was only fabricated around the catalytic sites whereas DNAzyme-free regions did not form any polymer. However, polymerization directly on DNA origami templates required specific adjustment to the ionic strength of the system to balance reaction kinetics and DNA stability. Weil and co-workers adapted the HRP-mimicking polymerization system for the shape-controlled formation of polydopamine (Figure 15B).^62^ Normally, dopamine tends to self-polymerize in neutral and basic pH, yielding a highly adhesive polymer comprising a multifaceted structure of covalent and noncovalent interactions that is not yet fully understood. By conducting the polymerization in acidic milieu with the help of DNAzymes, the group could implement significant control over dopamine formation. Various polymer patterns on a DNA nanosheet were accomplished, and furthermore, polydopamine acted as a “supramolecular glue”, shaping the origami conformation as the polymerization progressed. It is important to recognize that a slightly acidic pH was crucial for successfully controlling polymer formation as well as the ionic strength of the reaction. High ionic concentration disfavored the electrostatic interaction of dopamine and subsequent reaction intermediates with the DNA template, giving rise to polymerization in solution instead of the origami surface. Nevertheless, DNA stability has to be monitored closely when operating in ion-deficient environments. Recently, the Weil group further developed the method by switching from a chemically induced polymerization to a photo triggered variant allowing the control of the reaction over time (Figure 15C).^64^ G-quadruplexes were employed to trap the photosensitizer protoporphyrin IX at distinct positions on 3D DNA origami tubes, and upon irradiation with visible light, polydopamine formation was induced without the need for further reagents. Not only was the process locally confined, temporal control was dictated by simply switching off and on the light. Despite the noncovalent nature of the polydopamine–DNA hybrid structure, electrostatic interaction between polydopamine and the DNA survived the total depletion of ions in aqueous medium, confirming its high binding capabilities. Additionally, it was shown that just the presence of one or two polymer rings was sufficient to confer stabilization of the DNA origami in pure water.

In the studies reviewed here, the patterning of polymeric structures on a DNA template via noncovalent interactions remains relatively scarce due to several aforementioned challenges. Complementary base pairing appears to be the most intuitive approach to arrange polymers along several ODN anchors on a DNA template; however, it necessitates a modification of both building blocks. Electrostatic coating leads to nonspecific coverage in general, yet various groups designed DNAzyme-based systems to locally restrict polymer growth from the DNA surface. The nanopatterning achieved using this method has allowed the customization of structures in nanoscale resolutions that are yet unachievable by other technologies.

### 3.3 Chemistry of DNA–Polymer Conjugates Postcoupling

#### 3.3.1 Chemistries on the Polymer

In the synthetic approaches reviewed above, we describe the conjugation of polymers to DNA where reactions are often compromised by the combined limitations of the polymer and the DNA. However, postfunctionalization of the DNA–polymer conjugate offers further access to manipulate nanostructure behavior and function. To realize these postfunctionalization prospects, functional groups need to be embedded in the polymer backbone or at the antipodal terminus. Depending on the polymer employed, modifications can be implemented at points along the backbone; however, functional groups must be compatible with or protected from the coupling chemistry employed for DNA conjugation. In doing so, there are several avenues for secondary polymer functionalization, such as cross-linking or small molecule attachment. Postconjugation polymer cross-linking of an amphiphilic polymer was demonstrated to create a nanoparticle bearing six ODNs.^166^ Here, a DNA nanocage was synthesized bearing 8 ssDNA–amphiphile conjugates at the cube corners. The amphiphiles were found to self-assemble in the hollow cube core, demonstrating the guided self-assembly through DNA structures. The polymer consisted of HE and amino groups (Am) in the sequence 5′-Am-(HE)3-Am-(HE)3-Am-DNA-3′ and was cross-linked with sebacic acid bis(*N*-succinimidyl) ester to produce the nanoparticle. Cross-linking the polymer chains provides the opportunity to alter the physical properties as well as structure and function, which will be considered in section 4.2.1.

Small molecular attachment is also possible through secondary coupling reactions. In one example, a PEG chain was functionalized by a NHS group at one terminal and a maleimide at the other.^167^ DNA coupling was performed through amine-NHS coupling, leaving the maleimide group available and unchanged. A thiol-modified folic acid could then be coupled through a thiol Michael addition to yield a doubly conjugated polymer chain. Although there are a few examples in the literature, the development of biorothogonal reactions directly fuels the available prospects of postconjugation functionalization of DNA–polymer conjugates. In doing so, this strategy can potentially enable an increase in functional diversity as well as the capability to program complex solution behavior.

#### 3.3.2 Chemistries on the DNA

Likewise, postconjugation modification can be achieved through the DNA block, offering several additional strategies unique to the DNA such as chain extension with PCR or hybridization of a complementary ssDNA. One drawback of DNA–polymer synthesis with a user-defined DNA sequence is the length limitation of solid phase synthesis. The polymerase chain reaction (PCR) is a well-established technique employed to replicate short (50 bp) to long (30 kb) lengths of DNA using specific primers to amplify the region of interest. In the case of DNA–polymer conjugates, PCR was employed to synthesize di- and triblock copolymer conjugates of defined DNA length and content bearing polymer termini.^71^ This PCR method employs presynthesized ODN–polymer conjugates as the primers, where the conjugation was produced via *grafting to*, to amplify a specific length of DNA. The amplification occurs through the binding of each primer to the long ssDNA sequence, followed by extension from the 3′-ODN-end by the DNA polymerase. This results in a long length of DNA bearing a polymer at the 5′-end. Monodispersity of the central block is therefore achieved and yields either a single or double terminal polymer conjugate determined by the primers used. Additionally, as the primers are independent of each other, the conjugated polymer can be varied and therefore can bear two alternative polymers as part of the triblock, for example, PEG–DNA–PPO or NIPAM–DNA–PEG. In this example, amplification was employed to reshape the conjugate structure. Amplification can also pose benefits for downstream binding or signal enhancement. Rolling circle amplification is an alternative DNA replication technique where DNA sequences are copied from circular DNA. In the case of DNA–polymer conjugates, ssDNA penetrating from the conjugate is available to perform primer functions for DNA polymerase extension from the 3-end. Specifically, ssDNA was bound to polymers at the 5′-end and could bind to the circular DNA at designated positions through complementary base pairing.^168^ Once bound, the DNA polymerase can perform the extension and displacement for continued amplification resulting in long ssDNA protruding from the polymer conjugate. Through the employment of postmodification, the long DNA chain is not present during the conjugation reaction, thus avoiding steric challenges. Therefore, the use of postcoupling extension to the DNA block through PCR and RCA demonstrates a synthetic approach to long DNA–polymer conjugates. Chemistries could also be envisaged on DNA postcoupling. As with the polymer reactions, the compatibility with the DNA–polymer coupling reaction is required.

### 3.4 Characterization of DNA–Polymer Conjugates

The characterization of DNA–polymer conjugates requires a range of techniques due to the variety of approaches and products synthesized. In particular, the production scale of DNA–polymer conjugates has been a primary concern, which is often the bottleneck for analytical tools with poor limit of detection. For conventional polymer synthesis, there are two key techniques: gel permeation chromatography (GPC) and dynamic light scattering (DLS), which are the benchmark characterization techniques to determine polymer quality. For both techniques, approximately 10–50 nmoles of conjugate is required to provide an adequate signal for analysis. This often restricts the number of experimental variables one is able to explore due to the limited amount of material available. GPC and DLS analysis can be applied to all conjugates formed through the *grafting to* approach as polymers can be fully characterized prior to attachment. For the *grafting from* approach, DLS and GPC^19,74,85,86^ are still applicable to determine the polymer length of 1D DNA–polymer conjugates; however, polymer characterization can pose greater challenges when 2D or 3D nanostructure conjugates are fabricated. Hence, for 2D and 3D conjugates, AFM and high resolution transmission electron microscopy (TEM) can be used to determine the presence of the polymer conjugate on the DNA nanostructures. Spatial information in 2D/3D architectures of the polymer remains a challenge as it is often difficult to determine the orientation of the attached molecules. The overall structure, such as a rodlike or spherical micelle, however, can be determined.^74^ Alterations to the DNA-block can also be analyzed by AFM.^169,170^ In one instance, the height increase of the micelle due to dT incorporation by a terminal deoxynucleotidyl transferase (TdT) polymerase could be monitored to assess DNA chain extension.^170^ Agarose and PAGE are also useful techniques to confirm successful attachment or polymerization through band shifts. PAGE is often the preferred method of electrophoresis due to the increased resolution and can be employed to analyze covalent and noncovalent attachment. Using PAGE, *grafting from* can be monitored by the appearance of a new band at a higher molecular weight depicting successful polymerization,^19,74,85^ as can *grafting to*—the higher molecular weight band depicts efficient conjugation.^17,18,88,96,98,99^ A band shift can also be noted on noncovalent attachment due to the increase in size or also due to the change in overall charge—DNA coated in positively charged polymers is either retarded or can even migrate toward the negative electrode. In addition, the formation of more complex nanostructures can also be analyzed by PAGE, where increasing molecular weight leads to a band shift of lower migration.^171^ Both electrophoresis methods can be employed for all DNA structures and preparation techniques; however, they do not show specifics, such as orientation or precise polymer length. Matrix assisted laser desorption/ionization spectroscopy (MALDI) can also be employed to demonstrate that the correct molecular weight has been achieved after conjugate synthesis via *grafting to*;^91^ however, conjugate flight can be challenging and not always achievable. The increase in experimental analysis techniques has enabled the analysis and therefore development of highly sophisticated DNA–polymer conjugates, although further developments would enable absolute visualization or precision of the DNA–polymer conjugates to assist this field of research.

## 4 Supramolecular DNA–Polymer Complexes

Supramolecular interactions between biology and synthetic materials have attracted more attention in recent years. Historically, the development of polymer and block copolymer assemblies has been widely regarded as the first entry of synthetic macromolecular objects in the understanding of the chain dynamics involved in complex solution processes. Biohybrid systems, in particular DNA–polymer conjugates, provide an intrinsic bridge to investigate supramolecular interactions promoted by the respective synthetic and biological entities. As expanded by previous sections, the chemical and physical orthogonality of both blocks allows them to be tailored largely independently, hence the potential for nanostructural design and supramolecular behavior to be studied in greater detail.

DNA–polymer conjugates behave like block copolymers, thus assembling into nanostructures due to hydrophobic and electrostatic interactions. Moreover, the sequence-specific interactions between DNA strands allow programmed assembly of exquisite nanostructures with stimuli-responsiveness, which is unique for block copolymers with DNA components. Meanwhile, the polymer segments display diverse chemical and physical properties, thus rendering more possibilities to tune the assembling behavior. Therefore, DNA–polymer conjugates can result in large diverse self-assembled nanostructures, which is one of the most attractive features of such hybrids. In this section, we classify the DNA–polymer assembled nanostructures into static and dynamic structures. The strategies to induce self-assembly, either by hydrophobic interactions through the polymer segment or by sequence hybridization by the DNA segments, are discussed for static structures, whereas the dynamic structures section mainly focuses on the stimuli that can trigger structural or behavioral changes.

### 4.1 Static Nanostructures

**Figure 16:**
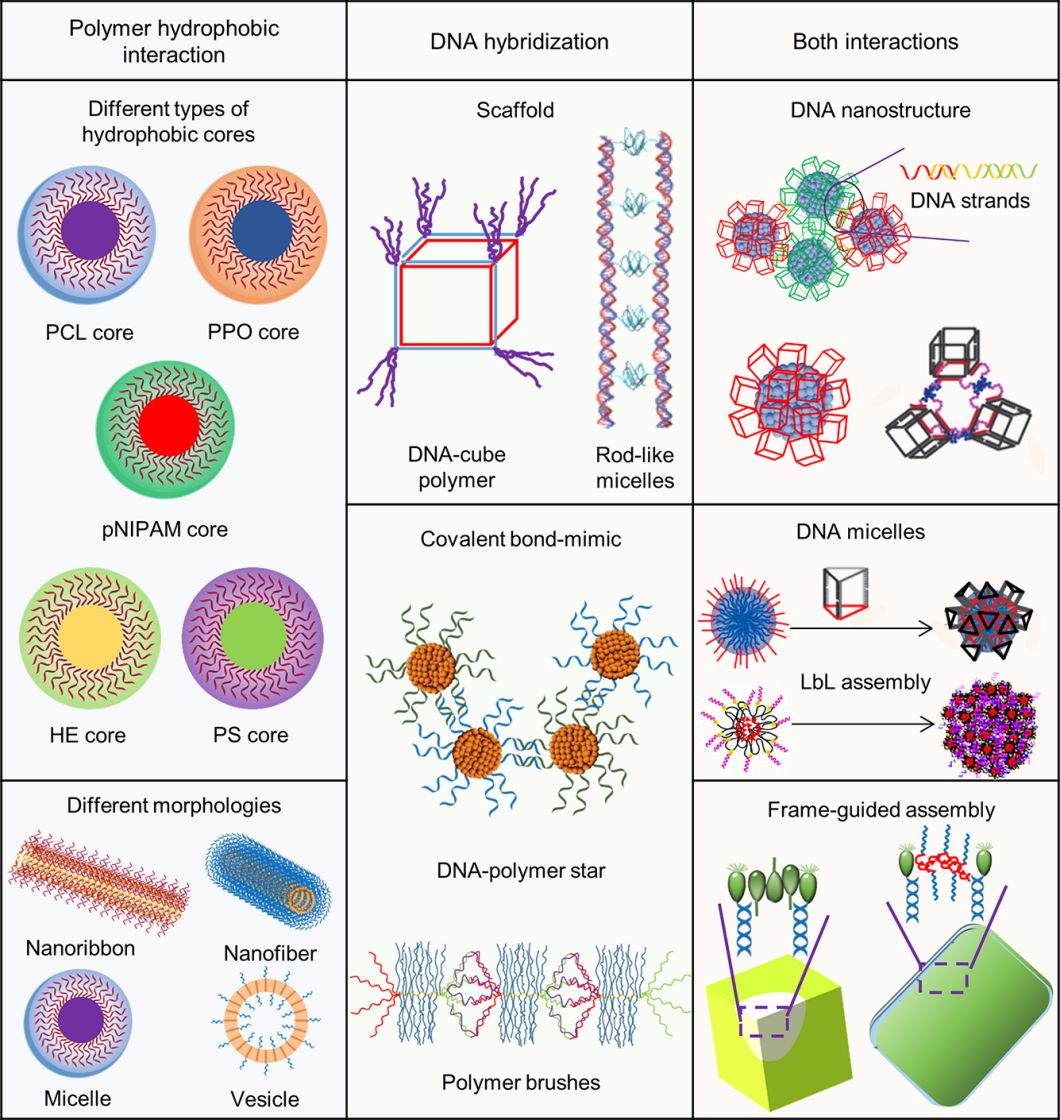
Summary of DNA–polymer static nanostructures. The assembly methods are categorized as a hydrophobic polymer interaction, DNA hybridization, and both interactions. Through polymer hydrophobic interactions amphiphilic DNA–polymer can self-assemble into DNA nanostructures with various morphologies.^172^ Reproduced with permission from ref (172). Copyright 2014 American Chemical Society. When the long DNA templates are regarded as the rigid scaffolds, rodlike micelles can be formed by DNA hybridization.^187^ Reproduced with permission from ref (187). Copyright 2007 John Wiley and Sons. The assemblies of static DNA–polymer superstructures can form through both DNA hybridization and the polymer hydrophobic interactions to guide the assembly of DNA nanostructures–polymer hybrids.^79,171^ Reproduced with permission from ref (79). Copyright 2016 American Chemical Society. Reproduced with permission from ref (171). Copyright 2014 American Chemical Society.

There has been much progress on the solution assembly of amphiphilic copolymers; however, the assembly of DNA–polymer conjugates has become a promising new field due to significant breakthroughs in DNA conjugation chemistry. When DNA is attached to the polymer to obtain a DNA–polymer conjugate, the properties of the conjugate are dictated by both the DNA and polymer component. One of the most common approaches to synthesize DNA–polymer nanostructures employs the hydrophobic interaction of polymers in combination with DNA sequence–specific recognition. Figure 16 shows the summary of DNA–polymer static nanostructures and their self-assembly principles. According to the properties of DNA and the polymer, the assembly methods of static DNA–polymer nanostructures can be classified into three types: assemblies induced by hydrophobic interactions through the polymer segment, assemblies induced by sequence hybridization via the DNA segments, and nanostructures involving DNA and polymer induced assembly.

#### 4.1.1 Assemblies Induced by Hydrophobic Interactions through the Polymer Segment

Due to the wide range of hydrophilic and hydrophobic properties that can be customized by synthetic polymers, DNA–polymer conjugates consist of two categories: hydrophilic and amphiphilic DNA–polymer conjugates. Amphiphilic DNA–polymer conjugates can self-assemble into nanoconstructions with various morphologies and are therefore attractive as unique nanomaterials. Amphiphilic DNA–polymer conjugates self-assemble into spherical micelles through the hydrophobic interactions with the aqueous solvent, exhibiting a hydrophilic DNA shell and a hydrophobic polymer core. The first DNA–polymer micelles, formed from DNA–PLGA polymers, were introduced by Park’s group.^90^ The formed micelles would continuously release ODNs by controlling the degradation of PLGA chains and exhibited enhanced cellular uptake by endocytosis thus leading to a new strategy for gene delivery. Since then, with the development of block copolymer self-assembly, DNA nanotechnology and DNA–polymer micelles with different hydrophobic polymer cores have been reported in succession.^110,112,121,122,173,174^Table 4 summarizes the construction of DNA–polymer micelles containing various hydrophobic cores with different properties.

**Table 4:**
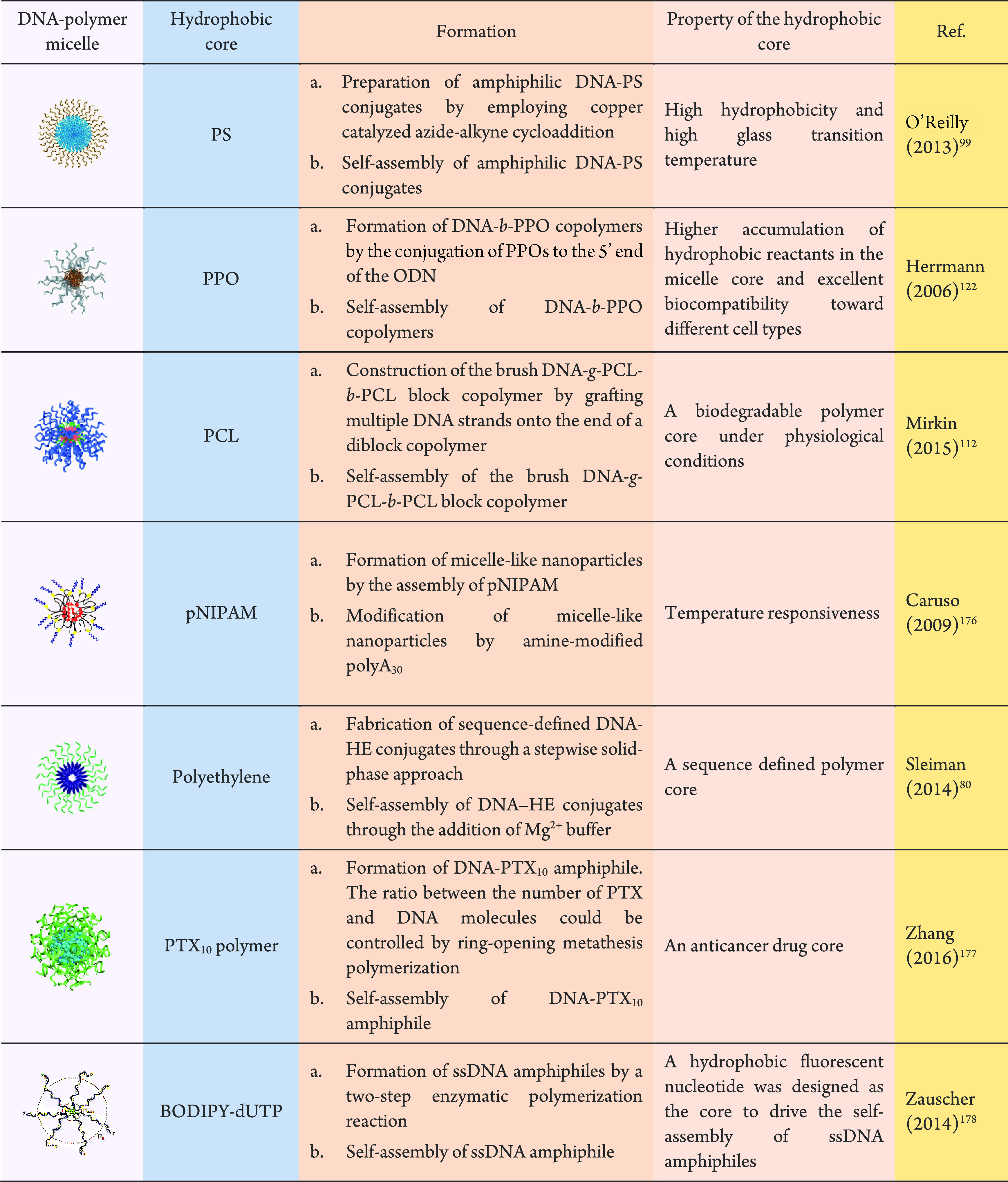
Construction of DNA–Polymer Micelles Containing Different Hydrophobic Cores Driven by the Hydrophobicity of the Polymer

Mirkin and co-workers prepared spherical DNA block copolymer micelles containing a PS core through self-assembly of PS-*b*-DNA conjugates.^121^ The PS-*b*-DNA block copolymers were fabricated through CPG solid phase synthesis. The micelles subsequently formed by hydrophobic interactions of PS-*b*-DNA copolymers, and the resultant hydrophilic DNA exterior of the micelles exhibited unique sequence-specific recognition properties. Furthermore, the average diameters of the micelle structures could be controlled by varying the DNA sequence length and PS molecular weight. Conversely, O’Reilly’s group employed copper-catalyzed azide–alkyne cycloaddition to synthesize the amphiphilic PS–DNA block copolymer.^99^ The resulting amphiphilic PS–DNA conjugate could then form micellar structures in aqueous solution. Whereas the conjugation efficiency achieved by Mirkin’s group was low, the copper-catalyzed azide–alkyne cycloaddition adopted by O’Reilly’s group resulted in PS–DNA conjugates in a 74% yield. The well-defined micelles were approximately 20 nm in diameter in O’Reilly’s work, which confirmed the amphiphilic properties of the PS–DNA block copolymer. Inspired by the synthetic strategy of Mirkin’s group, Herrmann and co-workers^122^ constructed a new class of DNA–PPO micelles. Phosphoramidite-PPO derivatives were obtained by the reaction of hydroxyl-group-terminated PPOs with phosphoramidite chloride. The corresponding derivatives were attached to the 5′-end of ODN on a solid support to obtain the DNA–PPO conjugates. The DNA–PPO conjugates self-assembled to form DNA–PPO micelles. Then, on the surface of the micelles several chemical reactions could be produced in a perfectly programmed and controlled manner. In addition to DNA amphiphiles, DNA triblock copolymers can also be developed to assemble DNA–polymer micelles. Gauffre and Mirkin both prepared DNA–polymer micelles containing PCL hydrophobic cores by using triblock copolymers.^112,174^ The PCL hydrophobic cores could then be gradually degraded under physiological conditions by cleavage of ester bonds with acid-promotion or esterase-catalysis.^175^ Gauffre et al. focused on micelle preparation and DNA-based recognition ability,^174^ whereas Mirkin’s group^112^ aimed to graft many more DNA strands onto the end of a diblock copolymer. Thereby they synthesized a micelle structure consisting of a DNA-brush block copolymer. The micelle exhibited a higher nucleic acids surface density, a higher melting temperature, and more effective cellular uptake without transfection agent. In contrast, Caruso’s group^176^ prepared pNIPAM-cored DNA micelles in an alternative two-step approach. First, at a temperature lower than its lower critical solution temperature (LCST), the pNIPAM terpolymer formed micelle-like nanoparticles. Second, an amino-modified polyA_30_ or polyT_30_ was conjugated to the micelle-like nanoparticles to obtain DNA–pNIPAM micelles. Although there have been some reports on the construction of DNA–polymer micelles using DNA triblock copolymers, with the development of DNA amphiphilic preparation technology in recent years, more work has been done to prepare DNA–polymer micelles using DNA amphiphiles. Sleiman and co-workers constructed HE–DNA micelles containing a polyethylene core by the self-assembly of either HE_6_–DNA or HE_12_–DNA conjugates.^80^ A stepwise solid phase approach was performed to sequence-specifically conjugate synthetic HE oligomers on DNA, which would form monodisperse DNA–polymer conjugates with a defined sequence. The formed DNA–polymer conjugates displayed self-assembly behavior, which could be tuned by the polymer length employed. Specifically, in a buffer with 10 mM Mg^2+^, HE–DNA conjugates containing five or fewer HE units existed as discrete molecules. In contrast, HE–DNA conjugates containing more than six monomer units could self-assemble to form DNA–polymer micelles. Furthermore, the successful preparation of the micelles was verified by encapsulating a guest molecule, Nile Red, within the hydrophobic core of the micelles. Zhang’s group also constructed a DNA–polymer micelle through the self-assembly of DNA–drug conjugates.^177^ By covalently binding nucleic acids and paclitaxel (PTX, an anticancer drug), the amphiphilic nucleic acid–drug conjugates could form micellar nanoparticles, which were structurally analogous to spherical nucleic acids (SNAs). This example treated the anticancer drug PTX as the hydrophobic core, which is discussed in more detail in section 5.3.

In addition to the series of methods reported above for preparing DNA–polymer micelles, Zauscher’s group used an enzyme-catalyzed polymerization reaction to construct starlike micelles.^178^ dNTPs were sequentially introduced to the oligonucleotide primer through the polymerase TdT. Thereby, ssDNA amphiphiles with high molecular weight and low polydispersity could be prepared by the enzyme-catalyzed polymerization reaction in solution. Through the incorporation of multiple hydrophobic unnatural BODIPY fluorophore-modified dUTP (B-dUTP) nucleotides at the terminus, ssDNA amphiphiles could self-assemble into the starlike micelles. In this work, the successful preparation of micelles was verified by agarose gel electrophoresis and AFM and well predicted by the dissipative particle dynamics simulations. The enzyme polymerization method has great potential in the field of drug carrier development as the DNA building blocks can also be adopted as the drug.

**Figure 17:**
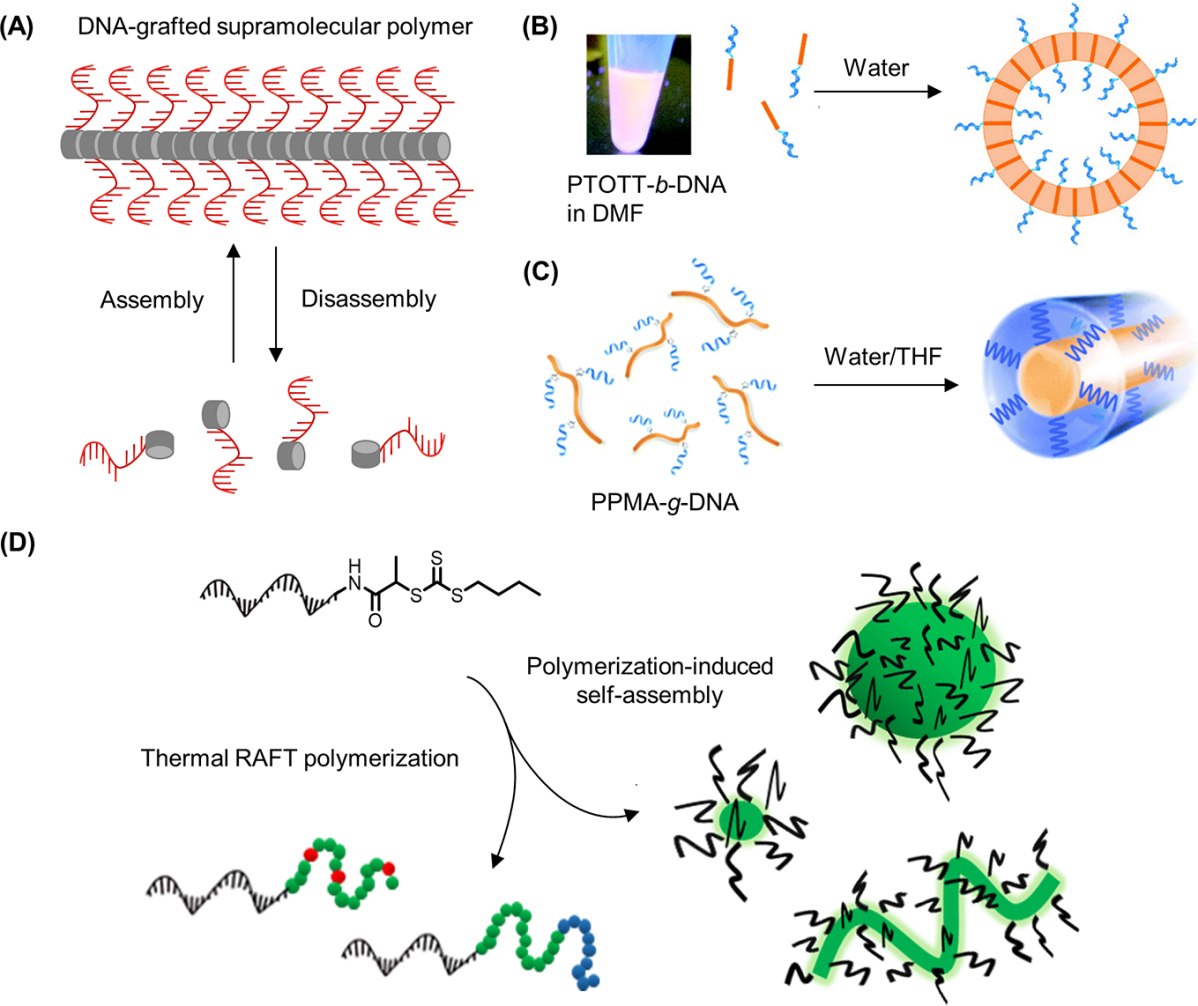
Construction of DNA–polymer static nanostructures with different morphologies assembly driven by hydrophobicity. (A) Schematic representation of 1D DNA–polymer ribbon structures via the self-assembly of chimeric pyrene–ODNs.^179^ Reproduced with permission from ref (179). Copyright 2015 John Wiley and Sons. (B) DNA-*b*-PTOTT conjugates were used to construct vesicles.^172^ Adapted with permission from ref (172). Copyright 2014 American Chemical Society. (C) Construction of PPMA-*g*-DNA nanofibers.^181^ Reproduced with permission from ref (181). Copyright 2015 the Royal Society of Chemistry. (D) Fabrication of DNA–polymer nanostructures of various shapes by targeted chain length synthesis of the polymer block.^74^ Reproduced with permission from ref (74). Copyright 2020 John Wiley and Sons.

In addition to spherical micelles, DNA–polymer conjugates have also been used to assemble into other morphologies. So far, DNA–polymer-based self-assembly has made the successful preparation of micelles, nanofibers, nanoribbons, and vesicles possible. In addition to these structures, via the self-assembly of chimeric pyrene–DNA oligomers, Haner’s group^179,180^ fabricated helical ribbon structures (Figure 17A). Here, the pyrene–ODNs were prepared through the conjugation of a pyrene segment with various lengths (0, 1, 4, or 7 units) to an ODN (10 nucleotides) at the 5′-end. Ultimately 1D ribbon-like DNA-grafted supramolecular polymers were formed due to stacking and hydrophobic interactions between pyrene chains. Unlike Haner’s approach, Park and co-workers prepared DNA-modified 1D polythiophene nanoribbons via a simultaneous assembly of DNA-*b*-poly[3-(2,5,8,11-tetraoxatridecanyl)-thiophene] (PTOTT) with PEG-*b*-PTOTT.^172^ PEG-*b*-PTOTT drove the self-assembly which offered an approach to attach the DNA’s molecular recognition properties to several self-assembling structures. In this work, not only DNA–polymer nanoribbons were prepared, but also size-controllable DNA–polymer vesicles by the assembly of DNA-*b*-PTOTT (Figure 17B). When only DNA-*b*-PTOTT was present, vesicle assembly was observed. The formation of vesicles was favorable due to the rigid polythiophene structure in combination with the high negative charge of DNA. In general, simple spherical micelles could form by the self-assembly of DNA–polymer conjugates due to the highly negatively charged DNA backbone. However, the π–π interaction of the rigid PTOTT block induced the self-assembly of DNA-*b*-PTOTT to form an unusual vesicle. In the self-assembly process of rod–coil copolymers, the trade-off between interface energy, coil stretching, and rod filling could affect the assembly structure. Vesicles are thermodynamically more advantageous than simple micelles—simple micelles formed through assembling rod–coil block copolymers possessed a high curvature and can create rod packing defects. Furthermore, the experiment verified that the increase in vesicle size was mainly dependent on increasing the polymer concentration. Subsequently poly(propargyl methacrylate) (PPMA)-*g*-DNA nanofibers were fabricated by Nardin’s group through the assembly of an amphiphilic poly(2-alkyl-2-oxazoline) (POX)-*graft*-DNA copolymer.^182^ Since POX is similar to a polypeptide in structure, it could be considered as a pseudo polypeptide. This was the first report of a DNA sequence and an assembly composed of a pseudo peptide through a nucleation polymerization mechanism. The nanofibrils were formed by inter- and intramolecular hydrogen bonding. Jiang et al. also successfully prepared a primary nanofiber structure using the PPMA-*g*-DNA brush. The nanofiber structure possessed a hydrophilic DNA shell and a hydrophobic PPMA core formed by DNA strands covalently grafted to a PPMA backbone via “click” chemistry (Figure 17C).^181^ Herein, PPMA selective solvents, such as THF, could influence the morphology of the PPMA-*g*-DNA nanofibers; for example, when 20 vol % THF was added to the aqueous system randomly interwound nanofibers were observed. Conversely, the introduction of 40 vol % THF drastically increased the tendency to form multistrand helices. The methods used in the examples described above provide DNA–polymer nanostructures with a single shape by altering a single factor. Weil’s group developed a new platform technology to construct DNA–polymer nanostructures with multiple shapes. This technology mainly leveraged PISA for polymerization from ssDNA to fabricate nanostructures.^74^ The solution-based thermal RAFT polymerization from DNA was achieved by enzymatic degassing of glucose, glucose oxidase, and sodium pyruvate under ambient conditions. Furthermore, this was the first time PISA was performed with RAFT polymerization from DNA and it provided a convenient path to construct complex DNA–polymer worm architectures. This technology was successfully used to prepare DNA–polymer conjugates with narrow molecular weight and variable lengths. The assemblies could reassemble into the thermodynamically most favored state with increasing degrees of polymerization. Subsequently, DNA–polymer nanostructures of various shapes were manufactured by targeting the chain length of the polymer block, which established a new platform technology toward functional DNA–polymer nanostructures (Figure 17D).

#### 4.1.2 Assemblies Induced by Sequence Hybridization of the DNA Segments

Amphiphilic block copolymers consist of a hydrophilic block and a hydrophobic segment that can self-assemble into various predictable morphologies fueled by the phase separating constituents.^183^ Although the chemistry of block copolymers is diverse and offers varying optimization approaches to tune stability and biocompatibility, it lacks sequence selectivity, monodispersity, programmability, and the fine structural control provided by DNA. With the advances in DNA nanotechnology, flexible structural manipulation of DNA has provided an intelligent tool to program the self-assembly of DNA block copolymer materials. In the previous section, static DNA–polymer nanostructures were discussed based on how polymer design can motivate self-assembly by hydrophobic interactions. This section reviews the static DNA–polymer nanostructure induced by sequence hybridization of the DNA segments and associated technologies.

**Figure 18:**
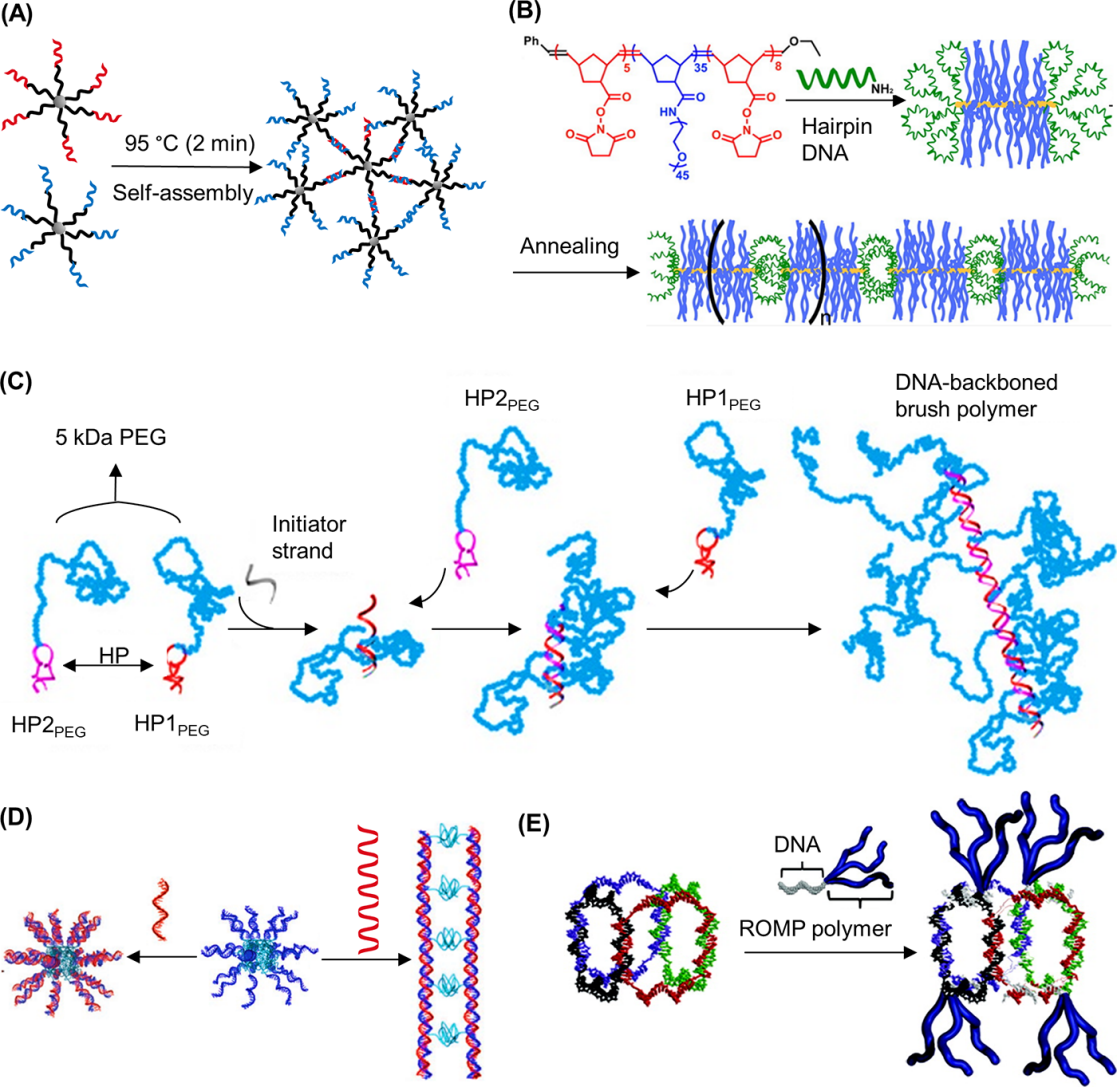
Static DNA–polymer nanostructure assemblies mediated by DNA hybridization. (A) Construction of star-polymer conjugates.^97^ Reproduced with permission from ref (97). Copyright 2011 American Chemical Society. (B) The formation of wormlike nanostructures by the self-assembly of a hairpin DNA–polymer conjugate via DNA hybridization.^113^ Adapted with permission from ref (113). Copyright 2014 American Chemical Society. (C) Schematic illustrations of forming a DNA-backboned bottlebrush polymer through DNA hybridization.^186^ Adapted with permission from ref (186). Copyright 2016 American Chemical Society. (D) Construction of ss-DNA-*b*-PPO micelles and rod by using short and long DNA templates, respectively.^187^ Adapted with permission from ref (187). Copyright 2007 John Wiley and Sons. (E) Formation of DNA cubes. DNA–polymer conjugates are organized onto a 3D DNA scaffold.^109^ Adapted with permission from ref (109). Copyright 2012 American Chemical Society.

In one assembly approach, the DNA component mediated the static DNA–polymer supramolecular complexes through complementary strand hybridization.^184^ Here, Das and co-workers prepared star-polymer–DNA conjugates via the click reaction, where subsequent higher-order nanoassemblies could be achieved by complementary DNA hybridization, where DNA was treated as a covalent bond-mimic (Figure 18A).^97^ Zhang and co-workers reported a series of examples to assemble polymer–DNA conjugates via DNA cross-linking. In one study, nucleic acids were covalently conjugated to the termini of triblock copolymer brushes to yield interactive handles which could then form head-to-tail ordered wormlike supramolecular nanostructures (Figure 18B).^113^ In this assembly process, intermolecular DNA hybridization was prevented by the triblock copolymer brushes, inducing the multivalent conjugate to mimic a divalent structure. The DNA moieties were designed to attach complementary brushes, thus forming well-defined 1D nanostructures. This elegant design was ensured by two key parameters. First, the directionality of assembly needs to be controlled by a sufficiently rigid polymer backbone so that the monomer cyclization is energy-unfriendly. Second, the number of DNA strands at one termini is critical to avoid multiple connections on one block. Their further studies also investigated the self-assembly kinetics and provided a model to accurately predict the degree of polymerization and size distribution of the assembled products.^185^ Moreover, in order to improve the biopharmaceutical properties of ODN therapeutics, in another example, they developed a DNA-backboned bottlebrush structure with PEG side chains (Figure 18C).^186^ Here, the PEGylated ODN hairpins were constructed to realize a hybridization chain reaction, which lead to a living polymerization using two hairpins as monomers. Thereby, a “bottom-up” synthetic approach was devised to obtain the uniformly PEGylated DNA nanostructures. The position and number of the PEG chains could be accurately controlled throughout the nanostructure surface.

Another assembly model used for DNA-mediated static DNA–polymer supramolecular complexes is to regard DNA as a rigid scaffold for organizing polymer molecules at specific locations with particular direction and number.^184^ Herrmann and co-workers achieved the preparation of rodlike micelles by using long DNA templates as scaffolds.^187^ This work clearly reflected how DNA hybridization altered the structural properties of DNA block copolymer micelles, transforming the ssDNA shell of the micelles into dsDNA through base complementation. When the short complementary sequences were introduced to spherical DNA block copolymer micelles through base pairing, a spherical DNA-*b*-PPO micelle with a double stranded corona could be fabricated and the overall shape of micelles was conserved. However, when hybridized with long DNA templates, the morphology of the spherical micelles was transformed from spheres to uniform rods (Figure 18D). Four and five DNA-*b*-PPO polymers were annealed on two long DNA templates, T88 and T110, respectively. Upon hybridization, the spherical ssDNA–polymer micelles were disintegrated and DNA-*b*-PPOs were arranged linearly along the long DNA strand. Eventually, nucleic acid segments participated in the formation of a double helix structure with the template, and hydrophobic blocks protruded from dsDNA, resulting in spherical micelles transformed into a rodlike structure. Using a different form of DNA nanotechnology, Sleiman’s group built a new type of biohybrid material in which the positions of polymer chains were programmed in 3D treating DNA cages as scaffolds (Figure 18E).^109^ DNA cubes composed of four 80-nucleotide strands were fabricated by a new method that could be quantitatively assembled from a minimal number of DNA strands. In this instance, the polymer component, hexanediol, aided the nanostructure formation by increasing the flexibility and reducing strain. Hence, four hexanediol insertions were introduced into these DNA cubes at the junctions where the square faces were formed. The DNA–polymer conjugates were synthesized by combining a block copolymer with a short DNA strand and were subsequently introduced into the cubes by DNA hybridization, allowing the arrangement of a precise number of polymers on a specific surface of the cube. In this study, DNA was treated as a rigid scaffold, which could be used to sequence polymers remotely, as well as to control quantity, density, and direction.

#### 4.1.3 Nanostructures Involving DNA and Polymer Induced Assembly

**Figure 19:**
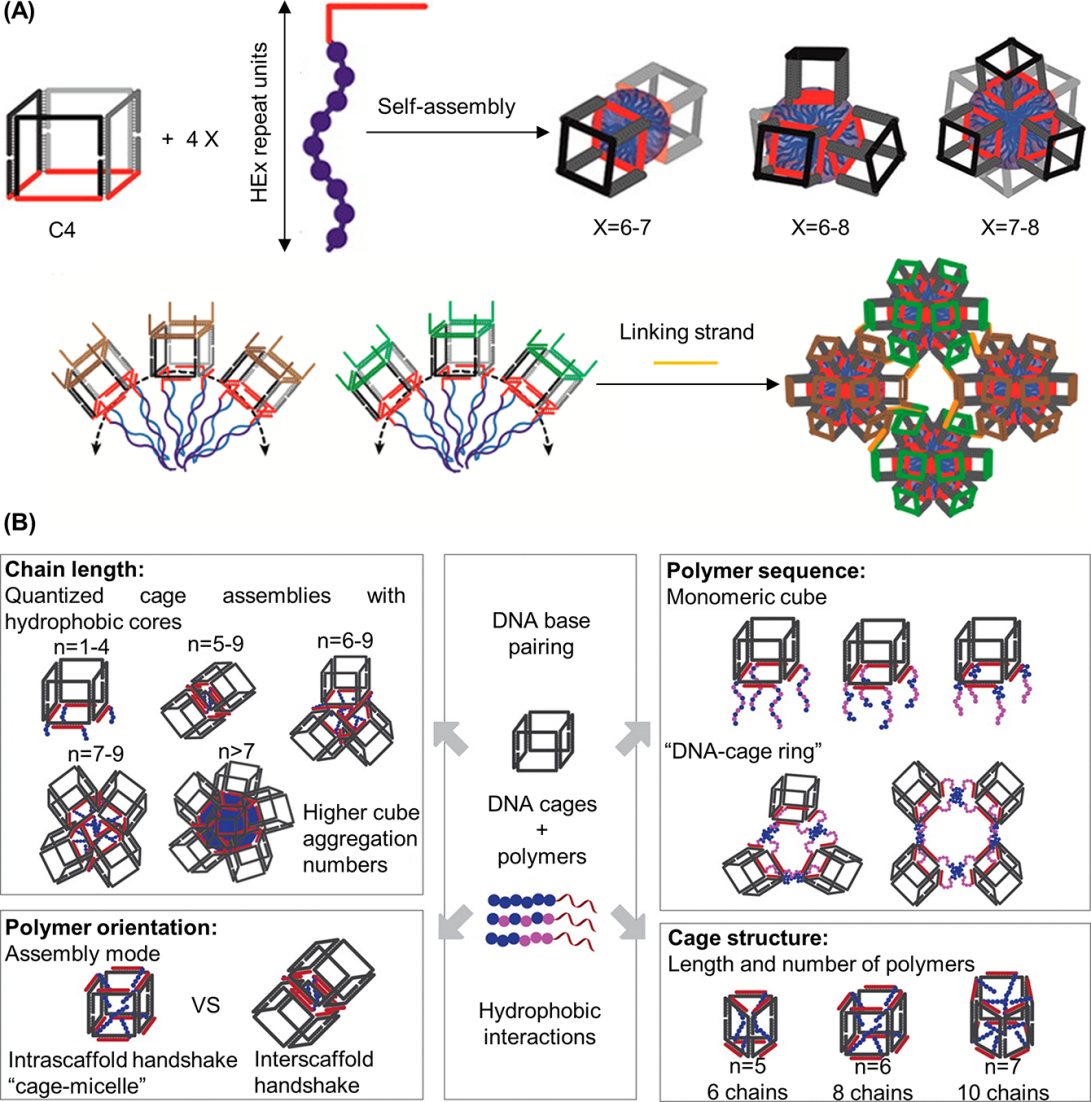
Static DNA–polymer nanostructure assemblies mediated by the hydrophobicity of the polymer and DNA hybridization. (A) Construction of micelle-of-cubes by HE-DNA strands with different polymer lengths attached to C4.^171^ Adapted with permission from ref (171). Copyright 2014 American Chemical Society. (B) Schematic illustrations of the self-assembly of sequence-defined hydrophobic polymers on DNA cages.^79^ The magenta circles represent hydrophilic monomers, and blue denotes hydrophobic monomers. “N” presents the number of hydrophobic repeats. Adapted with permission from ref (79). Copyright 2016 American Chemical Society.

In this part, we will discuss static DNA–polymer nanostructures that were assembled through both DNA hybridization and the polymer hydrophobic interactions. From the previous section, the regulation of polymer hydrophobicity can promote the assembly of ssDNA-conjugated polymers into ribbons, micelles, or other forms in water. The DNA strands of these self-assembled systems could present the thermodynamically favored radial arrangement around the core. However, polymers can also be exploited to mediate DNA superstructure formation by guiding the assembly of DNA nanostructure–polymer hybrids. These polymers are mainly attached to the surface of DNA nanostructures by DNA hybridization. To date, only a small amount of DNA superstructure formation induced by hydrophobic polymer has been published. The first polymer-mediated DNA superstructure was reported by O’Reilly’s group by the assembly of a tetrahedron–pNIPAM conjugate.^99^ Herein, DNA–pNIPAM conjugates were attached to a DNA tetrahedron by DNA hybridization and, in the presence of excess polymer, formed polymer-decorated DNA tetrahedrons, which self-assembled into tetrahedron–pNIPAM composite nanoparticles. Due to the thermal-responsiveness of pNIPAM, the formed superstructures are of dynamic nature, which is described in detail in the next section. Similarly, Sleiman and co-workers constructed a series of new nanostructures through the self-assembly of DNA cages and sequenced polymers,^79,171^ as presented in Figure 19A. In this instance higher order nanostructures could be created through orthogonal applications of DNA nanotechnology and precision polymers, which exhibited an unprecedented level of control over the number of polymers.^171^ The work first varied the multiplicity position and multiplicity of hydrophobic polymers on DNA cages and found that C4 (four binding regions on a single face), a geometric structure that promoted the aggregation of proximal polymers and thus superamphiphiles, was hence selected for self-assembly of a micelle containing the cubes. The number of aggregated DNA cages was programmed by changing the number of hydrophobic polymer repeating units on the cages. With increasing length of the polymer, the monomeric (polymer chains potentially aggregated on one face) structures gave way to a higher-order dispersed assembly of the dimer and an increase in the finite number of aggregates, followed by a monodisperse oligomer micelle. Moreover, higher-order micelle assemblies were also possible by recognition of the external DNA nanostructures to create functional macroscopic materials. A network of micelles were formed by attaching different chains to the micelles of two different populations and then adding a connecting chain (Figure 19A, bottom). This constituted the first reported example which used quantified polymer to mediate self-assembly.^171^

The same group further investigated the limits of their self-assembly method and its value for application by adding hydrophilic monomers to the hydrophobic polymer.^79^ In this system, Sleiman and co-workers focused on the investigation of systematic change in cage size and structure as well as the orientation of individual polymer chains on the DNA scaffold. Importantly, this study elucidated the assembly behavior of precision polymers attached to DNA cages by changing the length of the polymers, by adjusting the polymer sequence and direction of individual polymer chains on the DNA cage, and by varying the shapes of the DNA cages. When the hydrophobic polymer was attached to one face of the DNA cage by DNA hybridization, “quantified cage assemblies” with a hydrophobic core were obtained. The number of hydrophobic repeats directly affected the aggregation number of the cage (Figure 19B, top left). In this work, the polymers were sequence-controlled by precisely changing the number of hydrophilic and hydrophobic repeats. By introducing hydrophilic segments into the polymer, polymers could guide conjugates to form monomeric cages or donut-shaped “cage-rings” (Figure 19B, top right). Furthermore, the diameter and density of DNA cage-rings could be adjusted by controlling the length of the polymer blocks. By studying the orientation of polymer chains on the cages, a DNA cage intrascaffold “handshake” to form DNA–micelle cages was demonstrated. When both sides of the DNA cage were decorated with hydrophobic polymers, it was more stable than the cage that was unsubstituted (Figure 19B, bottom left). Additionally, the successful formation of the hydrophobic core in the fabricated DNA–micelle cages was verified by the encapsulation of the hydrophobic Nile Red. Finally, in order to probe whether the geometry of the cage can change the number of cages per aggregate, cages with different sizes and geometries were efficiently constructed (Figure 19B, bottom right). According to the experimental results, the different cage geometries resulted in an intrascaffold “handshake” within the scaffold with different capacities for small molecules and with various hydrophobic repeats. The loading capacity of hydrophobic guests could be increased when the larger cages were used to assemble the cage-micelles.

**Figure 20:**
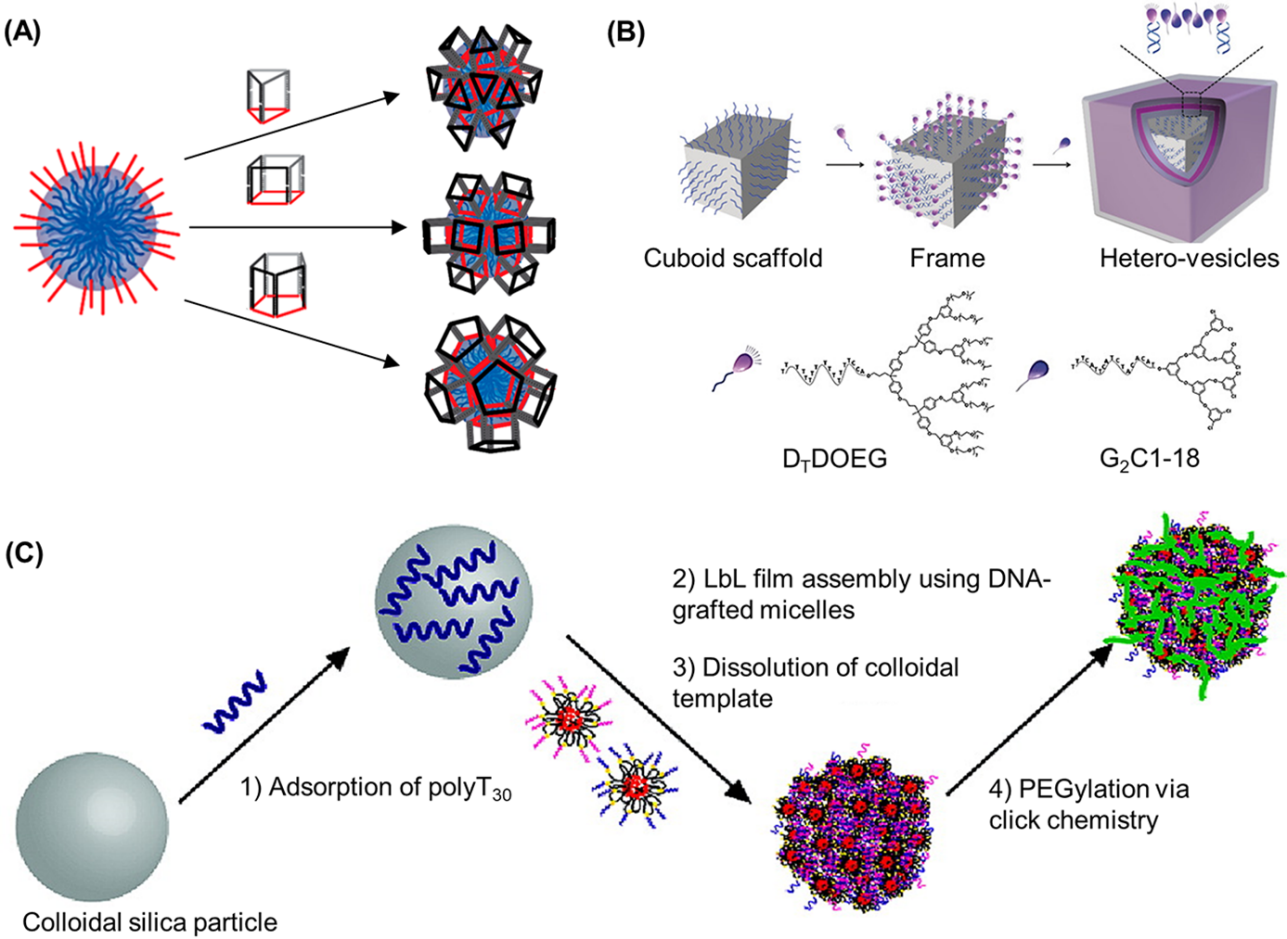
(A) Construction of micelle-of-cubes by incubating preassembled micelles with preassembled DNA nanostructures. Adapted with permission from ref (171). Copyright 2014 American Chemical Society. (B) Formation of cuboid vesicles by the FGA method on DNA origami scaffolds.^188^ Adapted with permission from ref (188). Copyright 2017 John Wiley and Sons. (C) Construction of PEGylated DNA–pNIPAM capsules through multilayer assembly of pNIPAM-T_30_ and pNIPAM-A_30_ micelles.^176^ Adapted with permission from ref (176). Copyright 2009 American Chemical Society.

Furthermore, DNA–polymer micelles could also be used to assemble static DNA–polymer supramolecular complexes. As shown in Sleiman’s work, they constructed a micelle-of-cubes by polymer hydrophobic interactions and DNA hybridization.^171^ Here, HE_12_–DNA micelles were fabricated by the assembly of polymer–DNA conjugates which has been discussed in section 4.1.1. Subsequently prisms with various geometry and size were adsorbed onto the surface of the HE_12_–DNA micelles to form micelle-of-cubes by DNA hybridization (Figure 20A). This assembly method was used to assess the integrity of DNA nanostructures attached to micelles. Conversely, Caruso and co-workers constructed DNA–polymer microcapsules employing a layer-by-layer assembly approach of DNA grafted polymer micelles.^176^ Here, amine-modified ssDNA was conjugated to pNIPAM to synthesize pNIPAM-T_30_ and pNIPAM-A_30_ micelles. Colloidal silica particles were used as templates to assemble pNIPAM-T_30_ and pNIPAM-A_30_ micelles layer-by-layer. The silica particles were then dissolved to obtain the DNA–polymer microcapsules, which possessed lower permeability than single-component DNA capsules due to the presence of pNIPAM (Figure 20B).

In general, vesicles, spherical micelles, or other symmetric forms can be assembled by amphiphilic molecules in water. However, obtaining amphiphilic assemblies with concrete sizes and shapes is still a significant challenge in this area.^189^ Liu’s group developed frame-guided assembly (FGA), an approach to prepare a series of shape-controlled DNA–polymer amphiphilic assemblies.^188,190,191^ This method could be employed to assist the assembly of polymers driven by the hydrophobic polymer outside the frame, which offered greater control over self-assembly. First, they employed DNA-functionalized AuNPs as the frame to fabricate customized heterovesicles.^190^ Prepositioned, discontinuous leading hydrophobic groups (LHGs) were introduced by DNA hybridization to the corresponding positions of the frame, outlining the edges of the designed structures. Other amphiphilic molecules could be guided to fill the gaps between LHGs and ultimately produce monodispersed vesicles. This work improved the understanding of the fundamental mechanism of self-assembly through the FGA method. Subsequently, a similar method was employed to fabricate a 2D nanosheet and 3D heterovesicle assemblies by constructing DNA origami nanostructures.^188,191^ A variety of DNA origami shapes could be prepared through the folding of a long scaffold DNA sequence by a series of carefully designed short DNA strands. The ssDNA-modified amphiphilic molecule named DDOEG was positioned reasonably on the DNA origami platform through sequential specific DNA hybridization to form a 2D hydrophobic framework domain on the DNA origami. Consequently, a homogeneous or heterogeneous 2D nanosheet could be gradually formed on the DNA origami by absorbing additional amphiphilic molecules into this hydrophobic frame domain through the hydrophobic interactions.^191^ Through this study it was finally demonstrated that the FGA strategy can overcome the obstacle of 2D amphiphile assembly in aqueous solution. Importantly, the size and shape of the 2D amphiphilic assemblies could be readily controlled by the shape of the DNA origami. Additionally, by varying the design of the DNA origami scaffolds, cuboid and dumbbell-shaped heterovesicles could be constructed.^188^ As shown in Figure 20C, once the DNA origami nanostructure was formed, 115 copies of polyA strands were positioned on the surface. Subsequently, the polyT-modified D_T_DOEG (selected as the LHG) could be introduced to the surface of the DNA origami through DNA hybridization. The added amphiphile molecules, G_2_Cl-18 (see Figure 20C for the specific structure), were assembled along the frame under the guidance of LHG, filling the gap between LHG and forming the heterovesicle. Altogether, the results in these articles demonstrated the flexibility of the FGA strategies to guide specific geometrically challenging amphiphilic assemblies by using the geometric programmability of DNA origami nanostructures.

In summary, this section systematically studied the formation of static nanostructures according to the properties of the polymer and DNA segment. As a traditional direction, the assembly behavior of DNA–polymer conjugates has been widely studied. Currently, different shapes of static nanostructures such as micelles, nanoribbons, nanorods, and microcapsules have been successfully prepared by the corresponding DNA–polymer conjugates. Among them, DNA–polymer micelles were studied the most. However, although the structures are now expansive, only a few types of polymers have been explored in conjugation with ssDNA. We believe that in the future, as the conjugation chemistry develops, more synthetic polymers with distinct structures and properties could be conjugated to DNA terminals and, therefore, the variety of DNA–polymer micelles would be further expanded.

### 4.2 Dynamic Nanostructures

**Figure 21:**
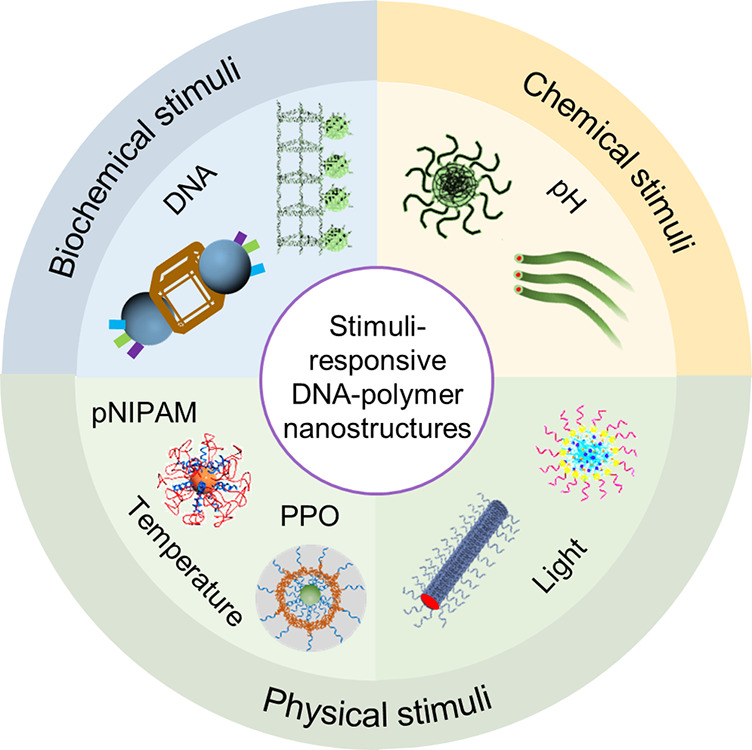
Schematic illustrations of different types of stimuli-responsive DNA–polymer dynamic nanostructures.^93,200^ Adapted with permission from ref (93). Copyright 2003 American Chemical Society. Adapted with permission from ref (200). Copyright 2013 American Chemical Society.

Unlike static DNA–polymer assembly, smart dynamic DNA–polymer nanostructures can alter their shape and size in response to external stimuli,^192^ which has been greatly investigated by several groups.^98,192,193^ As shown in Figure 21, these structures can respond to chemical stimuli (e.g., pH), biochemical stimuli (e.g., ssDNA), and physical stimuli (e.g., light and temperature). They are widely used in biomedical fields, such as intracellular delivery of genes via a pH-mediated mechanism and light-guided delivery of small molecule drugs. In this section, we discuss the dynamic nanostructures formed by DNA–polymer conjugates under each stimuli category.

#### 4.2.1 DNA Programmable Dynamic Nanostructures

Due to stimuli-responsiveness, the precise sequence-control, desirable molecular properties through base pairing, and ease of modification, DNA could also be used to mediate the programmed assemblies of dynamic DNA–polymer supramolecular nanostructures. The dynamics of these types of materials are mainly reflected by the strand displacement strategy and the reversible transformation of complementary base pairing (RTCBP).

**Figure 22:**
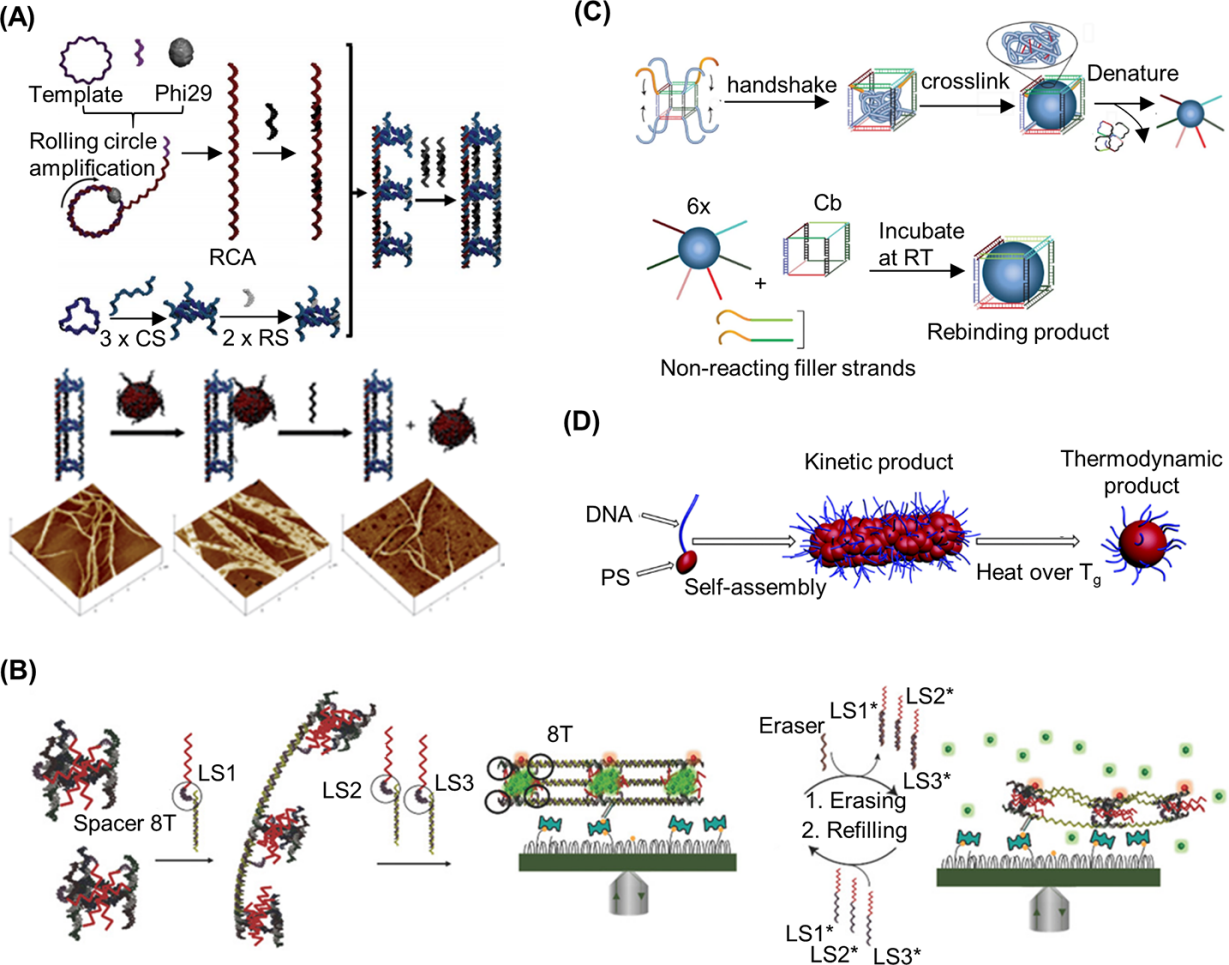
Use of DNA for reversible structural control of dynamic supramolecular DNA–polymer nanostructures. (A) Construction of DNA programmed nanotubes through a rolling circle amplification process.^98^ Reproduced with permission from ref (98). Copyright 2012 the Royal Society of Chemistry. (B) Schematic illustration of the assembly of dynamic nanotubes with hydrophobic pockets.^193^ Adapted with permission from ref (193). Copyright 2018 John Wiley and Sons. (C) The construction and denaturation of the cube with eight amphiphiles after cross-linking.^166^ Adapted with permission from ref (166). Copyright 2018 Springer Nature. (D) Schematic presentation for the kinetic micellization of DNA_6-mer_-*b*-PS_8.5kDa_ and subsequent reversible micelle morphological changes.^125^ Adapted with permission from ref (125). Copyright 2015 the Royal Society of Chemistry.

Sleiman and co-workers fabricated a series of dynamic DNA–polymer supramolecular nanostructures by the strand displacement strategy. In their first work, a long DNA strand containing a repeating sequence was created by the use of rolling circle amplification and then employed as a guide strand to construct robust nanotubes with a non-nicked backbone (Figure 22A).^98^ Subsequent block copolymer assemblies were sequence-specifically and longitudinally positioned on robust DNA nanotubes. These materials were dynamic, and the block copolymer structures could also be cleanly removed from the DNA nanotubes when a specific competitor DNA sequence was added (shown in Figure 22A). The second work employed the strand displacement strategy, providing an economic approach to build DNA nanotubes functionalized with lipid-like polymers.^193^ A spacer was used to link polymers to the nanotube, and polymers folded inside to create a hydrophobic environment within the nanotube. The spacer was vital to the morphology of the dynamic DNA–polymer nanostructures. A network of DNA bundles was formed when the polymers were directly linked to the nanostructure without spacers. However, in the presence of 8T spacers on the amphiphilic strands, the micellar microenvironments were generated along the repeating units of the nanotubes due to the DNA amphiphiles accumulating in the nanotube (Figure 22B). These micellar microenvironments were constructed mainly to encapsulate the small molecule Nile Red. Subsequently, a series of specific DNA strands were designed to interact with the 8T spacer, and the amphiphilic strands LS 1–3 were removed by strand displacement, which illustrated the dynamics of this nanostructure. Small molecules encapsulated in the nanotube would ultimately be released from the nanostructures when specific DNA strands were added. Another type of dynamic DNA–polymer nanostructure was also developed to demonstrate that not only small molecules but also nucleic acid therapeutics could be delivered by DNA–polymer dynamic nanostructures. Sleiman and co-workers designed a stimuli-responsive spherical nucleic acid, which could be used to load and release nucleic acid therapeutics.^194^

This spherical nucleic acid vehicle was assembled from only four strands, and nucleic acid therapeutics could be delivered upon recognition of specific ODN triggers via strand displacement. This work led to a new class of responsive drug delivery vehicles that were stimuli-responsive and more accessible than previous examples.

Besides strand displacement, DNA hybridization is also thermally responsive; thus, it could be reversible depending on the temperature, and therefore, it is possible to exploit to induce dynamics to the nanostructures. For instance, as shown in Figure 22C, Sleiman’s group constructed dynamic supramolecular DNA–polymer nanostructures through the RTCBP.^166^ Here, the hexavalent printed particle was formed inside the DNA cage. Under thermal denaturing conditions the hexavalent particles with different DNA strands could be obtained and hence precisely controlled in terms of the number of DNA strands and their directionality, while preserving sequence anisotropy. These hexavalent printed polymeric particles could reassemble into well-defined structures by scaffold rebinding and then again be released from the DNA scaffold via strand displacement. In this work, the dynamics of DNA nanostructures were reflected in both strand replacement and reversible DNA hybridization. In addition, Zhang and co-workers assembled dynamic DNA-PS supramolecular nanostructures through the RTCBP (Figure 22D).^125^ In this example, the DNA–polymer micelles were prepared first and then used to form higher-order assemblies. Here, the kinetic and thermodynamic self-assemblies of several DNA-*b*-PS amphiphiles were investigated via DNA hybridization. The kinetic products were obtained through a pair of complementary DNA-PS micelles, and the thermodynamic DNA–polymer micelles formed again when heated to 99.5 °C for 10 min.

#### 4.2.2 Temperature-Responsive Dynamic Nanostructures

In recent years, temperature-responsive polymers, as “intelligent” materials sensitive to temperature, have been widely studied. These polymers show mainly hydrophobicity changes in response to altered temperature and can therefore be used in the preparation of “dynamic polymer” materials. Among them, pNIPAM and PPO are the two most widely studied thermoresponsive polymers, which can be coupled to DNA to form DNA–polymer conjugates.^99,192,195,196^ When the temperature changes, the hydrophobicity of the polymers increases, leading to the formation of the DNA–polymer conjugates into temperature-responsive DNA–polymer dynamic nanostructures. In the following sections recent work in this area is discussed.

##### DNA–pNIPAM

pNIPAM has been widely studied as a thermoresponsive polymer, and as a linear form, at room temperature it is water-soluble. However, when the LCST is exceeded, pNIPAM transforms from hydrophilic to hydrophobic as the chains collapse with the entropic release of water. Therefore, pNIPAM can be used to prepare temperature-responsive materials. The first example of a DNA–pNIPAM conjugate was reported by Maeda’s group.^197^ In this work, a DNA–pNIPAM conjugate was used to capture a DNA-binding genotoxin, ethidium. Subsequently, DNA–pNIPAM conjugates could be widely used to assemble dynamic DNA–pNIPAM nanostructures due to the reversible phase-transition properties of pNIPAM. Furthermore, according to the type of DNA–pNIPAM conjugate adopted, the formed dynamic DNA–pNIPAM nanostructures could be divided into two categories: assemblies driven by ssDNA–polymer conjugates and DNA nanostructure–polymer conjugates, respectively.

**Figure 23:**
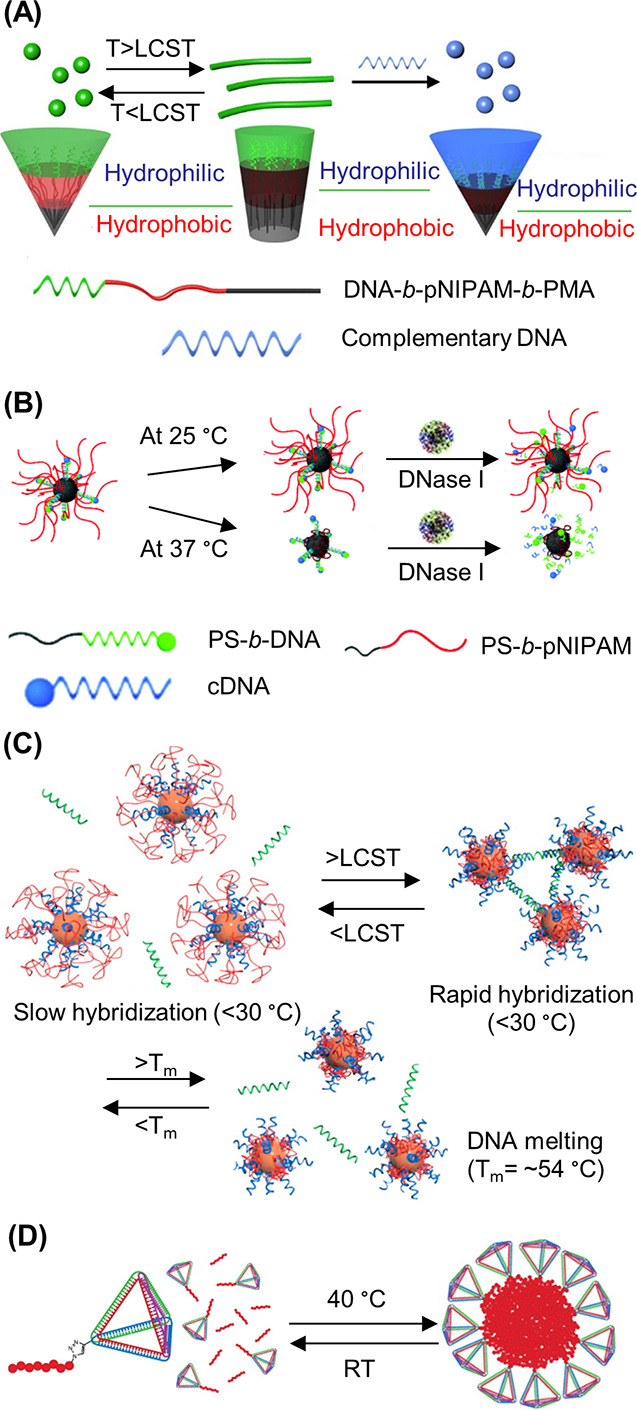
Fabrication of thermoresponsive dynamic DNA–pNIPAM nanostructures. (A) Schematic illustration of the dual-responsive DNA triblock copolymer dynamic nanostructures. The dual-responses were reflected in the temperature change and DNA’s molecular recognition properties.^192^ Adapted with permission from ref (192). Copyright 2016 American Chemical Society. (B) Nuclease-catalyzed degradation of hybrid temperature-responsive DNA micelles.^199^ Reproduced with permission from ref (199). Copyright 2019 the Royal Society of Chemistry. (C) Construction of DNA-functionalized AuNPs and verification of the recognition characteristics of DNA molecules on the surface of AuNPs.^200^ Adapted with permission from ref (200). Copyright 2013 American Chemical Society. (D) Temperature-induced formation of “surfactant”-stabilized nanoparticles by the self-assembly of the DNA tetrahedron–pNIPAM conjugate.^99^ Adapted with permission from ref (99). Copyright 2013 American Chemical Society.

The first class of dynamic ssDNA–polymer conjugates were formed by the well-established hydrophilic–hydrophobic transition of ssDNA–pNIPAM nanostructures. Maeda and co-workers connected pNIPAM-SH to 5′-maleimide-modified DNA to form the DNA–pNIPAM conjugates by using the Michael addition reaction.^198^ When the solution temperature rose above the LCST of the DNA–pNIPAM conjugates, micelles were produced via the self-assembly of the conjugates. The subsequent experimental results demonstrated that the large size polymer micelles could produce non-cross-linking aggregation and exhibited colloidal stabilization induced by terminal mismatch. The dynamics of the temperature-responsive supramolecular assemblies was also demonstrated by morphological changes. Park’s group assembled dual-responsive DNA triblock copolymer dynamic nanostructures (molecular recognition of DNA and temperature-responsiveness of PNIPAM) through constructing a thermally switchable triblock copolymer, DNA-*b*-pNIPAM-*b*-pMA (Figure 23A).^192^ Both DNA and pNIPAM were hydrophilic at temperatures below the LCST of pNIPAM, and hence, the overall hydrophilic/hydrophobic volume ratio favored the formation of spherical micelles.

However, when the solution temperature exceeded the LCST, pNIPAM became hydrophobic which induced the increased volume fraction of the hydrophobic part, reducing the micellar interfacial curvature and transforming the spheres into cylinders. This shape transformation was reversible. The cylinder could be transformed into a spherical shape when the temperature dropped below the LCST. In addition to the temperature response, the system was also designed to respond to DNA. The binding of “stimulus” DNA induced cylinder-to-sphere morphological changes due to the increase of the hydrophilic block volume (Figure 23A, right). The same group modified ssDNA with PS and then used ssDNA-PS and PS-pNIPAM to successfully prepare other dynamic DNA–polymer micelles which contained a DNA/pNIPAM corona and a PS hydrophobic core (Figure 23B).^199^ Through experimental design it was found that pNIPAM strands present a significant steric hindrance to bind to DNA immobilized on nanoparticles. However, by increasing the temperature above the LCST, the steric hindrance could be minimized as the conformation of pNIPAM would change from the extended form to the collapsed form, demonstrating the switching behavior of the resulting DNA–polymer micelles. The dynamics of the structures could also be reflected by the nuclease-catalyzed degradation. As shown in Figure 23B, when the temperature was above 37 °C, pNIPAM assumed the collapsed state and DNA sequences were degraded by DNase I. Conversely, when the temperature was lower than 25 °C, the dynamic DNA–polymer micelles were stable and DNA sequences could not be degraded by DNase I. The above work used PS as the hydrophobic core and described the response of reversible hidden or exposed DNA sequences to temperature cues. Mirkin and co-workers used a similar concept to fabricate a dynamic DNA–polymer micelle, with the hydrophobic core being replaced by AuNPs. As shown in Figure 23C, the DNA and pNIPAM were coassembled onto Au NPs.^200^ By increasing the temperature higher than LCST, pNIPAM shrank and the DNA sequences were exposed from the polymer surface, which induced the assembly of DNA–AuNPs by DNA hybridization, whereas, at a lower temperature, the formed complex assembly would be disassembled.

Another class of dynamic DNA–pNIPAM nanostructures was formed by using DNA nanostructure–polymer conjugates. pNIPAM could be conjugated to DNA nanostructures, followed by dynamic DNA nanostructures formation through the hydrophilic–hydrophobic transition of pNIPAM. Baumberg and colleagues designed a temperature-responsive DNA origami flexor by introducing a hydrophobic pNIPAM switch that reversibly regulates the DNA structure.^201^ The work paved the way for the intelligent design of preprogrammed nanomachines. O’Reilly’s group fabricated the dynamic tetrahedron–pNIPAM composite nanoparticles by adding an excess of pNIPAM homopolymer (Figure 23D).^99^ The DNA block copolymers could be used to assemble a DNA tetrahedron due to the DNA segment remaining sequence-specific during hybridization. Subsequently, due to the elevation of temperature above the polymer LCST, temperature-responsive dynamic structures with surface DNA tetrahedra would be formed when an excess of free pNIPAM was used.

##### DNA–PPO

**Figure 24:**
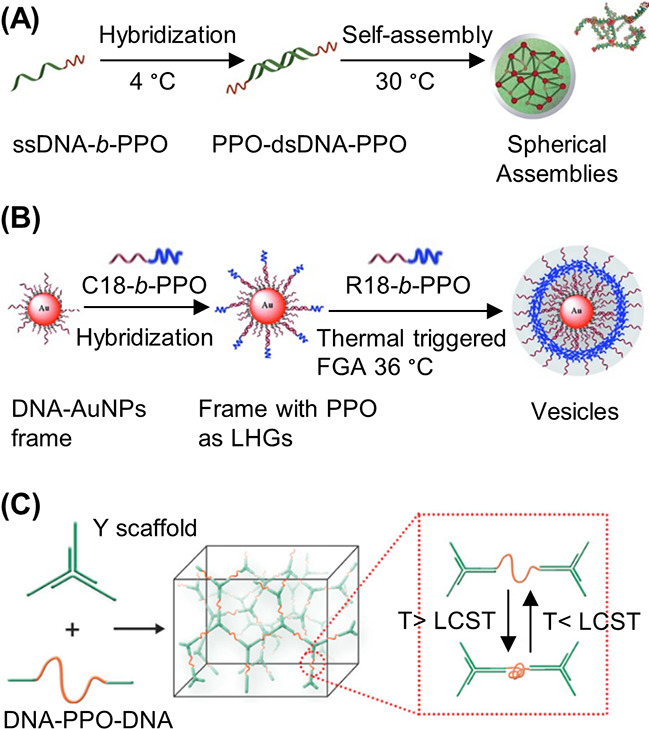
Dynamic DNA–polymer nanostructures formed by the self-assembly of DNA–PPO conjugates. (A) Construction of a supramolecular spherical DNA–PPO nanostructure.^195^ Adapted with permission from ref (195). Copyright 2015 American Chemical Society. (B) Illustration of the construction of temperature-responsive heterovesicles by the FGA strategy.^196^ Reproduced with permission from ref (196). Copyright 2014 John Wiley and Sons. (C) Construction of a 3D DNA network through the inset of temperature-responsive polymer PPO.^203^ Reproduced with permission from ref (203). Copyright 2018 John Wiley and Sons.

PPO is another temperature-responsive polymer, with many groups focusing on the assembly behavior of DNA block poly(propylene oxide) (DNA-*b*-PPO) copolymers currently.^122,173,202^ PPO displays hydrophobic characteristics at room temperature and is hydrophilic at lower temperatures (below 20 °C). As a result, dynamic spherical micelles in aqueous solution can be formed by the self-assembly of ODN-*b*-PPO. Liu’s group has made outstanding contributions to the development of the thermally responsive DNA–PPO dynamic structures,^195,196^ in which they first fabricated DNA–PPO dynamic nanostructures by the self-assembly of PPO-dsDNA-PPO triblock copolymers.^195^ The unimer of the inverse coil–rod–coil triblock copolymer PPO-dsDNA-PPO was formed via specific DNA hybridization below the LCST of PPO, which resulted in two thermally responsive PPO segments at the ends and a rigid dsDNA segment in the middle (Figure 24A). Subsequently, the spherical assemblies were self-assembled via hydrophobic interactions of PPO above the LCST by using the prepared triblock copolymer. Usually, DNA biblock copolymers self-assembled randomly without forming an ordered compact structure. However, the self-assembly process in this work was different from the DNA diblock copolymers commonly used. Subsequently, they employed the FGA strategy to fabricate heterovesicles with controlled size and shape.^196^ DNA-modified AuNPs were adopted as the frame (Figure 24B) to prepare the vesicles, and PPO was employed to make the LHGs thermally responsive. By DNA hybridization, the frame could attach DNA-*b*-PPO conjugates and the LHGs. After heating, the temperature-responsive heterovesicles were obtained due to hydrophobic transformation of the PPO segment and further induction of random DNA-*b*-PPOs around the AuNP frame. This work provided further information to understand the principles FGA and offered the chance to manipulate the FGA process by a thermal trigger. Moreover, the development of FGA enabled the construction of more complex and functional nanostructures.

To expand on the structures responsive to temperature, the triblock copolymer DNA–PPO–DNA was utilized to insert the thermally responsive polymer PPO into a 3D DNA network.^203^ This work verified the feasibility of the in situ study of the collapse of hydrophobic polymers in solution. By base pairing recognition, a DNA Y-shaped nanostructure could be connected by using DNA–PPO–DNA as the linker (Figure 24C). At a low temperature PPO was hydrophilic and could be uniformly distributed in the 3D DNA network, whereas increasing the temperature induced a hydrophobic change to PPO, which self-collapsed in the network. As the self-collapsing process was reversible, this strategy offered a good method to explore the nucleation-growing process of block copolymers. At the same time, this strategy also provided a good opportunity to study the molecular mechanism of mechanical properties of responsive materials.

#### 4.2.3 pH-Responsive Dynamic Nanostructures

Based on the unique properties of DNA and polymers, some supramolecular DNA–polymer complexes can exhibit pH-responsiveness. The DNA backbone is negatively charged and can be used to assemble pH-responsive DNA–polymer dynamic nanostructures through electrostatic interactions of DNA and cationic polymers. Numerous examples have shown that cationic polymers can form a nanocomplex by absorbing negatively charged DNA. The pH environment greatly affected the surface charge of the polymerized nanoparticles, and the loaded DNA could be intracellular released by a pH-mediated mechanism.^204−206^ Such pH-responsive systems are highly valuable for gene delivery, which has been intensively discussed in a previous review.^204^ Herein, we focus our discussion on pH-responsive dynamic nanostructures where at least one composition is a covalent DNA–polymer conjugate.

**Figure 25:**
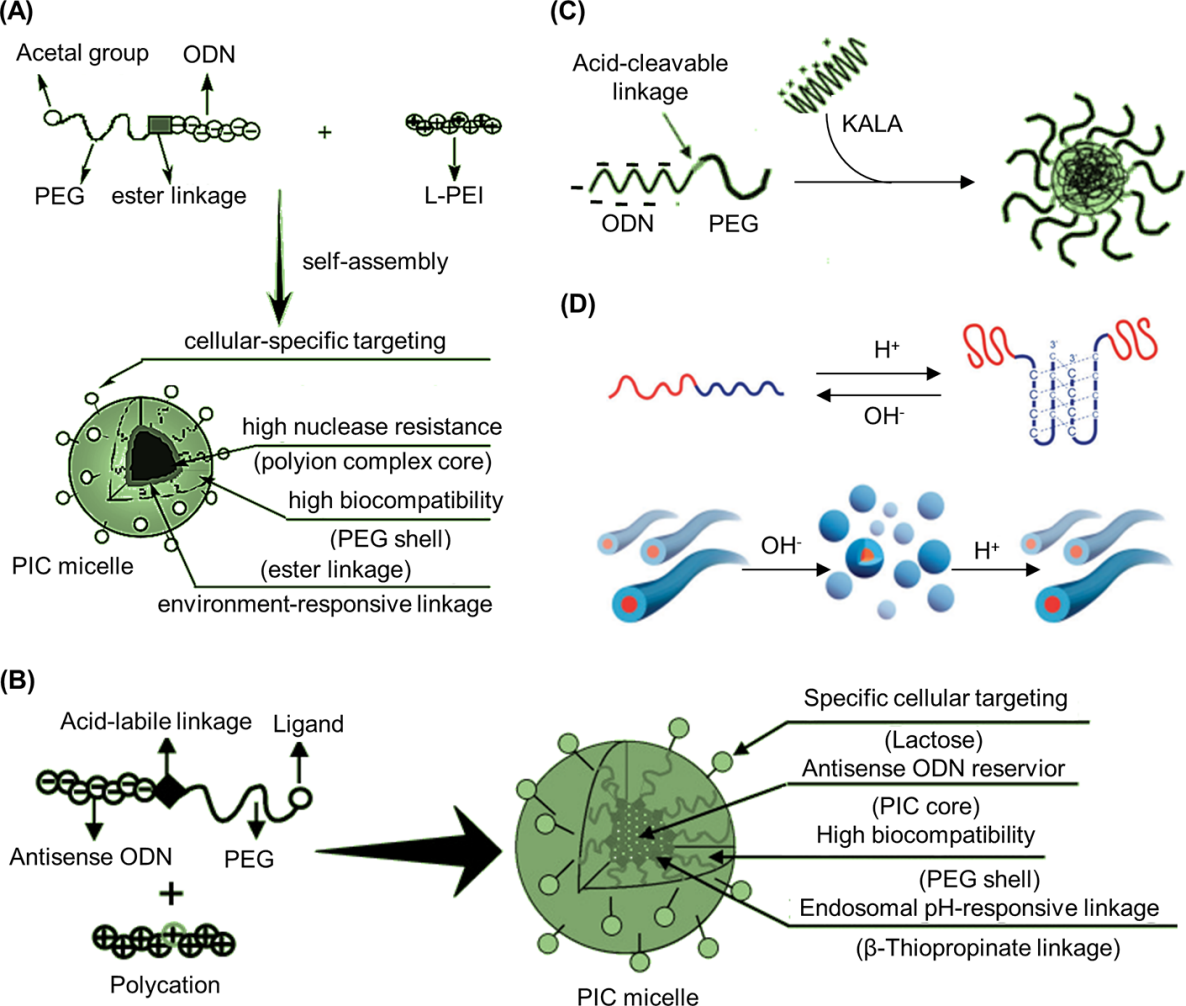
Construction of several pH-responsive DNA–polymer dynamic nanostructures. (A) Conceptual schematic illustrations of a pH-responsive PIC micelle formation.^94^ Adapted with permission from ref (94). Copyright 2003 American Chemical Society. (B) Construction of a new delivery system that is pH-sensitive and can target antisense ODN.^93^ Reproduced with permission from ref (93). Copyright 2003 American Chemical Society. (C) Construction of polyelectrolyte complex micelles by the self-assembly of ODN–PEG conjugates and peptide KALA.^207^ Reproduced with permission from ref (207). Copyright 2012 the Royal Society of Chemistry. (D) Reversible shape transformation of the pH-responsive DNA-*b*-PPO copolymer micelles.^95^ Adapted with permission from ref (95). Copyright 2005 John Wiley and Sons.

Other than the facile adsorption to DNA via electrostatic interactions to form pH-responsive DNA–polymer nanoparticles, DNA delivery could also be achieved by conjugating DNA with polymers via a pH-responsive linker. For instance, Kataoka and co-workers constructed pH-responsive polyion complex (PIC) micelles,^94^ which was the first report describing the design and synthesis of pH-responsive DNA–polymer dynamic nanostructures. The PIC micelle was formed through the association of a PEG–ODN conjugate and linear-PEI. A pH-responsive ester linkage (β-thiopropionate linkage) was introduced into the micelles to achieve the pH-responsiveness (Figure 25A). Subsequently, they developed a novel targetable antisense ODN delivery system, employing a PIC micelle. The system was intracellular environment-responsive and was composed of poly(l-lysine) (PLL) and a lactosylated PEG-antisense ODN (Lac-PEG–ODN) conjugate. An acid-labile linkage (b-propionate) was introduced into the micelle between PEG and ODN segments to realize the pH-response (Figure 25B).^95^ The experiment demonstrated that the lactosylated PIC micelles exhibited an antisense effect (65% inhibition). However, the lactosylated PIC micelles without any acid-labile linkage exhibited a decrease in antisense effect (65 → 27% inhibition). This suggested the endosomal compartment contained a possible cleavage of the acid-labile linkage which was in response to the lower pH. In a similar approach, Park’s group prepared pH-responsive polyelectrolyte complex micelles by the combination of the peptide KALA and the ODN–PEG conjugates (Figure 25C).^93^ The ODN–PEG conjugates were prepared by covalently conjugating the ODN, c-myb, to PEG, and an acid-cleavable phosphoramidate linkage was introduced into the conjugates. The phosphoramidate linkage could be cleaved completely within 5 h when the micelle was in an endosomal acidic condition (pH 4.7). This experiment mainly illustrated that the micelles were more efficiently transported into cells than the c-myb ODN and the polyelectrolyte complex micelles were a good carrier to deliver antisense ODN.

In addition to obtaining pH-responsiveness with a polycationic polymer or chemical linker, specially designed DNA sequences could also be used to impart similar responsiveness to the assembled structures. For example, Liu’s group reported a dynamic nanostructure with pH and temperature dual-responsiveness due to the combination of a thermoresponsive PPO and sequence-specific DNA strands.^207^ As shown in Figure 25D, a bimolecular “i-motif” could be formed through the folding of two DNA molecules of sequence, 5′-TTTCCCCTAACCCC-3′. Through the “i-motif” interaction two DNA-*b*-PPO conjugates were brought together and thus resulted in a triblock PPO–DNA–PPO copolymer. In addition, by adjusting the pH to slightly basic conditions the “i-motif” structure could be decomposed into random coils. Meanwhile, DNA-*b*-PPO copolymers could embody the characteristics of diblock copolymer under high pH. In this case, the assembly behavior of DNA-*b*-PPO was influenced by this stimuli-responsive nature. Hence, the morphologies of DNA-*b*-PPO dynamic nanostructures in an aqueous solution could eventually be transformed by adjusting the pH and temperature stimuli reversibly. DNA-*b*-PPO copolymers were assembled to form nanofibers at pH 5.0, and at pH 8.0 the morphologies would be transformed into spherical micelles (Figure 25D).

#### 4.2.4 Light-Responsive Dynamic Nanostructures

In addition to temperature and pH, light-responsive dynamic nanostructures were also constructed through supramolecular DNA–polymer complexes. Among the different stimuli, light is a cheap and easily manipulated clean resource, which can be precisely controlled in space and time and thus is unique in its administration. The wavelength of light is often the main design consideration that affects light-responsive materials. UV light is able to provide high energy, thus resulting in efficient light-responsive structural changes; however it is harmful for cells and tissues which is unfavorable for biomedical applications. The UV light may also lead to DNA damage, which must be considered when designing such systems. As such, visible light and even near-infrared light sources are more preferred. However, they have been rarely explored due to the lack of efficient photochemical reactions with low energy light. In general, only a few studies have investigated light-responsive DNA–polymer assemblies; therefore, there are several areas to explore to improve irradiation wavelengths and structural variety.

**Figure 26:**
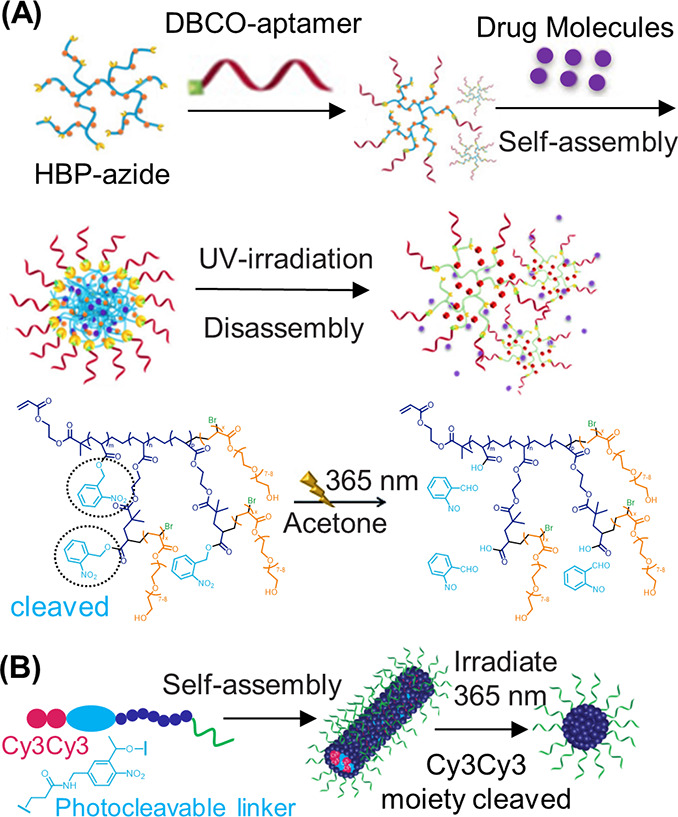
Different types of light-responsive DNA–polymer dynamic nanostructures. (A) Schematic of UV-promoted degradation of hyperbranched polymer (HBP)–OH. *O*-Nitrobenzyl moieties were introduced to the HBP side chains and would be cleaved under UV irradiation.^120^ Adapted with permission from ref (120). Copyright 2018 John Wiley and Sons. (B) Light-responsive shape-shifting of photocleavable Cy3Cy3-DNA nanofibers. The morphology of DNA nanostructures would be transformed from nanofibers to spherical nanoparticle due to the cleavage of Cy3Cy3 units from the structure under UV irradiation at 365 nm.^208^ Adapted with permission from ref (208). Copyright 2018 American Chemical Society.

Tan and Sleiman’s groups both provided seminal studies to the progress of UV-irradiation light-responsive dynamic nanostructures.^120,208^ Through the self-assembly of HBP–DNA conjugates, Tan’s group constructed a light-responsive drug delivery system (Figure 26A).^120^*O*-Nitrobenzyl derivatives were introduced to the delivery system to form the UV-responsive hydrophobic core. Under UV irradiation, the cleavage of *o*-nitrobenzyl moieties occurred, which reduced the hydrophobicity of the core and caused drug release through the disintegration of the nanoparticles. Conversely, Sleiman and co-workers fabricated 1D light-responsive DNA nanofibers by introducing a photocleavable linker to DNA–polymer conjugates (Figure 26B).^208^ As mentioned in section 4.1.1, assemblies induced by hydrophobic interactions through the polymer segment highlighted that the position of a cyanine (Cy3) dye played a crucial role in the shape-shifting of DNA nanostructures. The presence of Cy3 and its position in DNA–polymer hybridization were important for the formation of DNA nanofibers. The introduction of a photocleavable linker between the HE_12_ and Cy3Cy3 units would induce the formation of light-responsive DNA nanofibers. Furthermore, the morphology of DNA nanostructures was transformed from nanofibers to spherical nanoparticles through the cleavage of Cy3Cy3 units from the structure under UV irradiation at 365 nm.

In summary, this section mainly reviewed DNA–polymer dynamic nanostructures. From the above description, we can see that these dynamic DNA nanostructures are mainly generated by DNA regulation, temperature response, pH response, and light response. By the strand displacement strategy and the reversible transformation of complementary base pairing, DNA could be used to mediate the programmed assemblies of dynamic DNA–polymer supramolecular nanostructures. The introduction of temperature-responsive polymers such as pNIPAM and PPO and the addition of a pH-responsive linker or a photocleavable linker to polymers could also bring in stimuli-responsive dynamics to the system. Even though the examples of dynamic DNA–polymer conjugated nanostructures reported so far are still very limited, especially for the ones with clear application potentials, DNA-based dynamic nanostructures which did not incorporate polymers have been quickly developed in recent years. A series of temperature-actuated DNA nanopores, pH-actuated DNA-only devices utilizing Hoogsteen base pairing, and salt-actuated devices utilizing salt to mediate sticky end interactions between DNA pieces were fabricated.^209^ Even the electric field-actuated and magnetic field-actuated DNA nanodevices were also invented.^210,211^ Combining these dynamic DNA structures with polymers would pave the way to more smart and dynamic DNA–polymer systems with advantages of both DNA design and polymer properties.

## 5 Functionality of DNA–Polymer Conjugates

**Figure 27:**
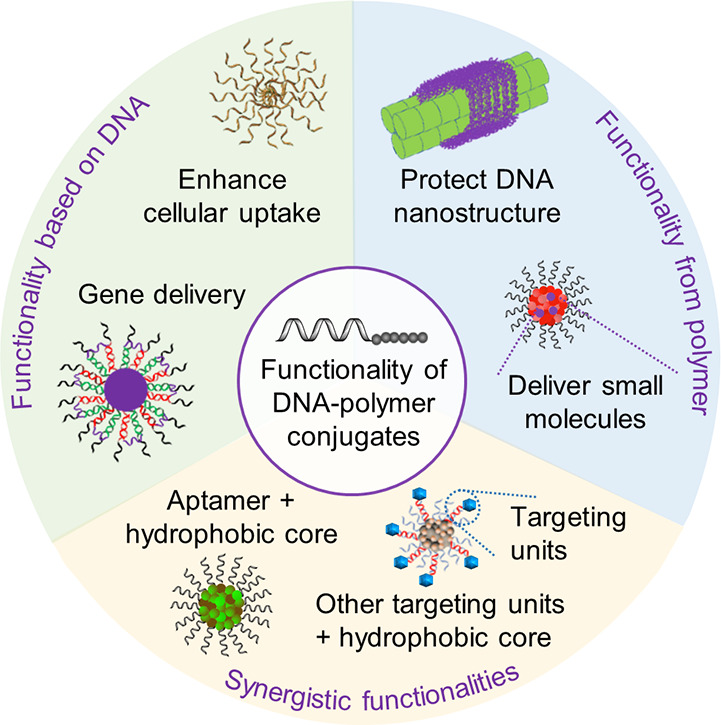
Functionalities of DNA–polymer conjugates. According to the hydrophobicity of polymers and DNA-based recognition ability, the functionalities of these DNA–polymer conjugates are mainly reflected in the protection of DNA nanostructures and used as drug carriers.

In recent years, the functions and applications of DNA–polymer conjugates have attracted more and more attention from scientists. Polymers are a class of widely studied materials, which consist of a range of types, functions, and applications. When polymers are conjugated to DNA, the functionality of DNA–polymer conjugates will be affected by polymer properties. Therefore, hydrophobic polymers attached to DNA induce the self-assembly of DNA–polymer conjugates to form DNA–polymer micelles with a hydrophobic core and a hydrophilic DNA corona. These structures have great potential applications in medicine and biology. Some types of hydrophobic polymers conjugated to DNA, due to their good biocompatibility, can be used to develop drug carriers to deliver small molecule drugs and nucleic acid therapeutics. Several hydrophilic polymers can also be attached to DNA and are often used to enhance the stability of DNA nanostructures. However, the functionality of DNA–polymer micelles is influenced not only by the polymer but also by the DNA. Several specific sequences of DNA can manifest different functions, such as targeting, catalyzing, and therapeutic action. These specific sequences of DNA can be introduced to DNA–polymer micelles, which will give new functionalities to DNA–polymer nanostructures. Therefore, as shown in Figure 27, this section can be divided into three categories according to the source of functionalities: (1) functionality from the polymer, (2) functionality based on DNA, and (3) synergistic functionalities.

### 5.1 Functionality from the Polymer

To date, many types of polymers such as PPO, pNIPAM, PS, and PCL have been conjugated to DNA to form DNA–polymer conjugates. Due to the different chemical and physical properties of the polymer, the polymer can vary the functionality of the corresponding conjugate. Based on the progress reported so far, these functionalities from the polymer are mainly reflected by the following two aspects: (1) small molecule drugs can be delivered by DNA–polymer conjugates due to the hydrophobic core of DNA–polymer micelles, and (2) polymers can be conjugated to DNA for stability enhancement of DNA–polymer conjugates.

**Figure 28:**
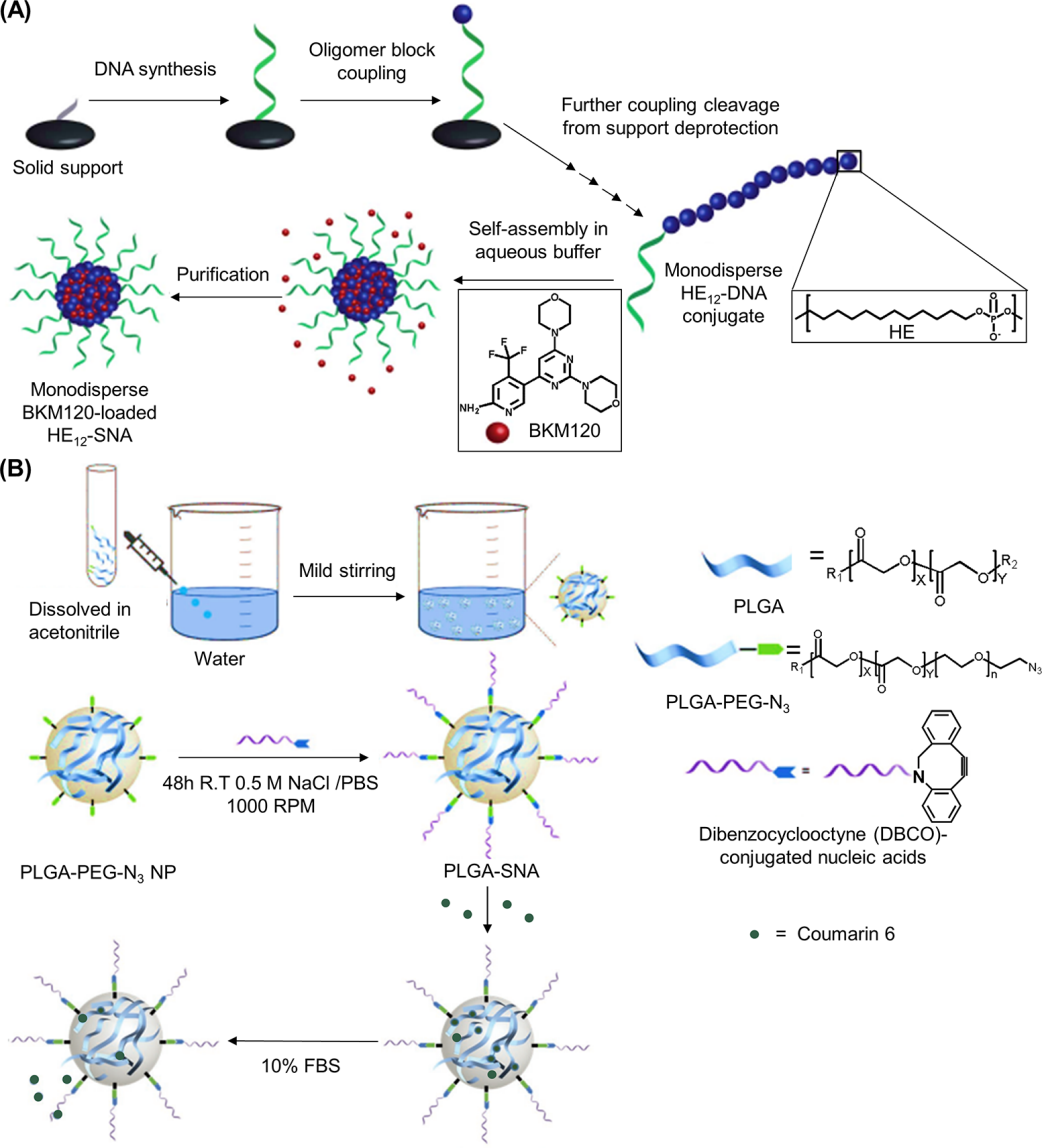
Construction of a spherical nucleic acid (SNA) system to deliver small molecule drugs. (A) Schematic representation of the self-assembly of the monodisperse BKM120-loaded HE_12_–SNAs.^212^ Adapted with permission from ref (212). Copyright 2017 the Royal Society of Chemistry. (B) Synthesis of PLGA-SNAs utilizing nanoprecipitation and coumarin encapsulation. Coumarin 6 encapsulated inside the PLGA hydrophobic core was utilized as a fluorescent model drug to evaluate drug-release kinetics.^213^ Adapted with permission from ref (213). Copyright 2018 John Wiley and Sons.

The amphiphilic block copolymers based on DNA–polymer conjugates can phase-separate into micelles containing a hydrophobic core and a hydrophilic DNA corona. Due to the hydrophobic interactions, several small molecule hydrophobic drugs can be complexed to the core of the micelle and delivered to the cells. As shown in Figure 28A, a novel delivery platform was constructed by Sleiman and co-workers to deliver small-molecule chemotherapeutics through the self-assembly of sequence-defined polymer–DNA conjugates.^212^ In this study, an SNA system was constructed to deliver the anticancer drug BKM120 (a small molecule drug with low water solubility) for the treatment of chronic lymphocytic leukemia. The DNA–polymer conjugates (HE_12_–DNA conjugates) were prepared via solid phase synthesis and subsequently used to form spherical micellar DNA nanoparticles in aqueous solution. The hydrophobic HE_12_ core of the formed DNA nanoparticles provided a favorable environment to encapsulate the hydrophobic drug BKM120. Meanwhile, this work also showed that HeLa cells had enhanced uptake of these structures, as well as their cargo internalization. Moreover, in vitro studies illustrated that BKM120-loaded HE_12_–SNAs induced cellular death and apoptosis. In addition, Mirkin’s group used an extremely facile strategy to construct another new types of polymer SNA conjugates which were comprised of PLGA NP cores that could be used to deliver small molecules (Figure 28B).^213^ PLGA–PEG–N_3_ nanoparticles were prepared under mild stirring followed by ODN conjugation to these nanoparticles to form the PLGA–SNAs. A hydrophobic model drug, coumarin 6, was encapsulated into the PLGA–SNAs and then released in a tunable manner depending on the polymer composition.

**Figure 29:**
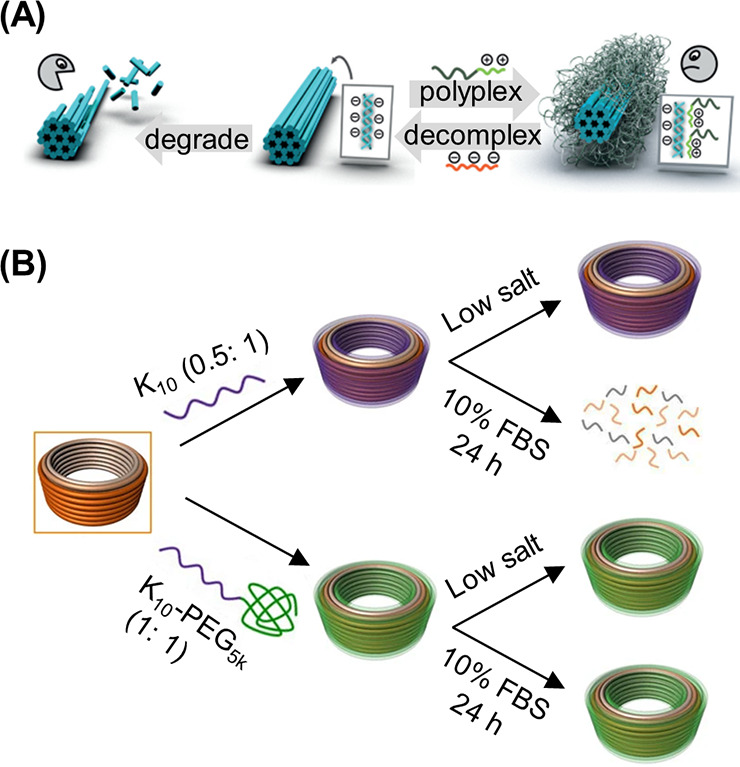
Protection of DNA nanostructures from nuclease digestion and denaturation through polymer coating strategies. (A) Cationic polymer was coated on DNA origami to form the polyplex micelles by electrostatic interaction.^160^ Native DNA nanostructures could be degraded easily by nucleases, but the formed polyplex micelles were resistant to nuclease digestion. Reproduced with permission from ref (160). Copyright 2017 John Wiley and Sons. (B) Schematic representing the differences in stability of native and coated DNA nanostructures in physiological buffers at 37 °C containing low salt and/or 10% FBS.^61^ Reproduced with permission from ref (61). Copyright 2017 Springer Nature.

The work described above highlights that DNA–polymer carriers have great potential for biomedical applications. However, as discussed in section 3.2.2, the in vivo nuclease activity could result in rapid degradation, which has strongly hindered their application. Therefore, the desire to enhance the stability of DNA–polymer carriers has attracted more attention. Zhang’s group explored the stability of ODN hairpins and demonstrated that the introduction of PEG side chains increased the resistance of DNA-backboned bottlebrush polymers against nucleolytic degradation and improved the thermal stabilities.^186^ Importantly, the PEGylation did not affect the hybridization of ODN hairpins. The stability of DNA nanostructures was also enhanced by coating polymers at the exterior. As shown by Schmidt’s group,^160^ coating with a cationic PEG–polylysine (p(lys)) block copolymer by electrostatic adsorption successfully enhanced the stability of DNA origami structures (Figure 29A).

The nuclease digestion of DNase I and FBS and denaturation under low salt concentration are the main factors affecting the stability of DNA structures. The formed robust shell, via PEG-p(lys) block copolymers coating around the DNA origami nanostructures, could protect the DNA structures from nuclease digestion and denaturation. As a direct, economical, and reliable approach, this protection strategy could be used to protect various types of DNA origami nanostructures. Moreover, it was demonstrated that the DNA origami template directly determined the shape of the prepared DNA origami polyplex micelles after coating with PEG-p(lys) block copolymers. Shih and co-workers adopted a similar approach to enhance the stability of DNA nanostructures. Through an oligolysine coating the major challenges which limited the effective use of DNA nanostructures in vivo were overcome.^61^ They found that DNA nanostructures coated with oligolysines were significantly more stable and were 10 times more resistant to DNase I in low-salt environments than in uncoated environments at 0.5:1 N:P (ratio of nitrogen in lysine to phosphorus in DNA) (Figure 29B). However, it is of important note that when N P ratios increased, DNA nanostructures coated by oligolysines aggregated. Oligolysine–PEG copolymers could also be used instead of oligolysines to coat DNA nanostructures to avoid aggregation, which resulted in up to 1,000-fold resistance to digestion by serum nucleases.

### 5.2 Functionality Based on DNA

**Figure 30:**
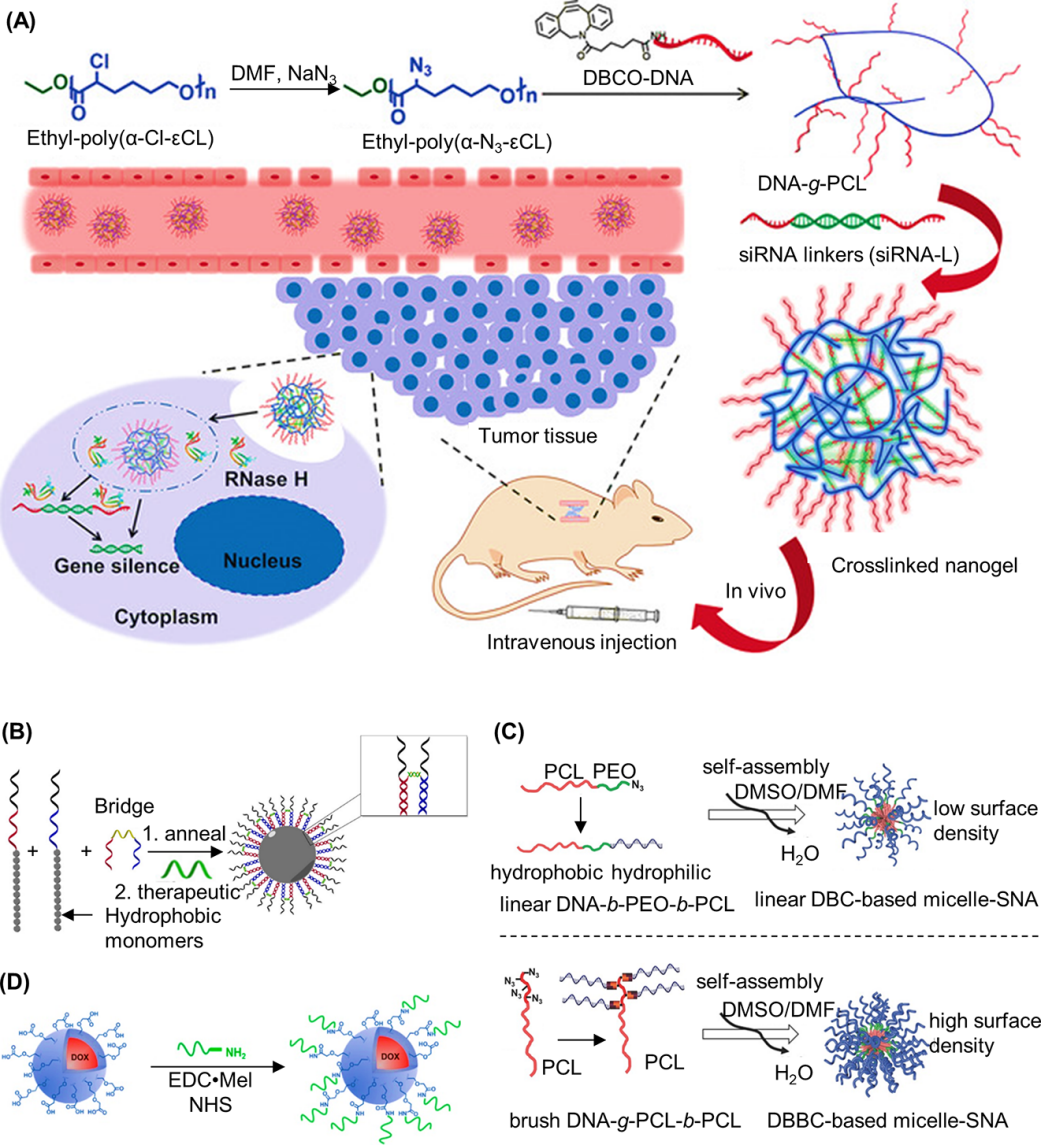
Example functionalities of DNA–polymer supramolecular nanostructures based on DNA. (A) siRNA was effectively delivered by a cross-linked nanogel through the complementation of bases between RNA and DNA.^214^ Reproduced with permission from ref (214). Copyright 2018 John Wiley and Sons. (B) The ODN therapeutic (green) was introduced to the responsive spherical nucleic acid (SNA) by DNA hybridization.^194^ Adapted with permission from ref (194). Copyright 2019 American Chemical Society. (C) Formation of micelle–SNA with different surface densities by using different DNA–polymer block copolymers, respectively.^112^ Adapted with permission from ref (112). Copyright 2015 John Wiley and Sons. (D) Amine-terminated ODNs were introduced into polymeric nanoparticles via amide-coupling chemistry to form the doxorubicin (DOX)-loaded polymeric SNAs.^111^ Adapted with permission from ref (111). Copyright 2017 American Chemical Society.

In this section, we discuss functionalities of DNA–polymer conjugates, which are based on DNA. These functionalities are mainly embodied in the delivery of genes and small-molecule cargos through the complementary pairing of DNA bases and more effective cellular uptake of DNA–polymer supramolecular nanostructures caused by nucleic acid shells. As shown by Haner’s group, functional DNA-grafted supramolecular polymers were designed and synthesized from monodisperse diblock oligophosphates.^180^ Through complementary base pairing, ssDNA chains arranged along the edges of the formed ribbons were available to load DNA-modified AuNPs, which served as a model cargo. Subsequently, Zhang and co-workers successfully realized the delivery of siRNA via nucleic acid hybridization.^214^ DNA-*g*-PCL brushes with complete water solubility were synthesized by grafting DNA onto a PCL trunk followed by the self-assembly of spherical and nanosized hydrogels by functional siRNAs cross-linking (Figure 30A). The nanogels delivered siRNA to different cells, generating effective gene silencing in vitro and in vivo to enable the development of a novel siRNA delivery system. In addition to delivering siRNA, antisense nucleic acid therapeutics could also be delivered by DNA–polymer supramolecular nanostructures as Sleiman’s group demonstrated (Figure 30B). They developed a novel responsive drug delivery vehicle to deliver nucleic acid therapeutics.^194^ This yielded a stimuli-responsive SNA which was assembled by monodisperse DNA–polymer conjugate interactions. Nucleic acid therapeutics were incorporated to the SNA via partial base complementation and could only be released through chain replacement in the presence of cytoplasmic genetic markers.

The nucleic acid shell of SNAs not only enables cargo loading and delivery of nucleic acid therapeutics but also facilitates the effective cellular uptake of SNAs. Mirkin and co-workers designed a strategy to construct micelle–SNAs with a biodegradable core and ODNs (Figure 30C)^112^ and demonstrated that the density of DNA affected its SNA-like properties. When the terminal segment of a diblock copolymer was connected with multiple DNA strands, the formed SNA would be induced with a higher DNA surface density. The DNA-brush block copolymer-based micelle–SNAs exhibited more effective cellular uptake than linear DNA block copolymer-based micelle–SNAs due to the higher surface density of nucleic acids. It was demonstrated that the cellular uptake would be enhanced due to the interaction of the ODN with class A scavenger receptors presented on the cell surface.^215,216^ In accordance with Mirkin’s theory, Nguyen’s group modified DOX-loaded polymeric nanoparticles with a dense ODN shell which greatly increased the cellular uptake of doxorubicin-loaded polymeric SNA (Figure 30D).^111^ Furthermore, the dense shell of ODNs could increase the colloidal stability of DOX-loaded SNA structures in biological media under physiological conditions. Not only does the density of DNA on the surface of a DNA nanostructure affect its cellular uptake efficiency, but the different shapes also have an effect. As shown in Figure 18D, the different lengths of DNA template introduced by DNA hybridization affect the morphology of DNA nanostructures. The introduction of long DNA templates induced the morphology of the spherical micelles transforming from spheres to uniform rods. The cell uptake experimental study illustrated that even though the components of the DNA nanostructures were the same, the cellular uptake efficiency of the rod-shaped polymer particles was 12 times more efficient than their spherical counterparts.^217^

### 5.3 Synergistic Functionalities

The reports above describe functionalities of DNA–polymer conjugates from the polymer and DNA, respectively. The following section focuses on the synergistic functionalities from the polymer and DNA which are mainly reflected in the design and development of targeted drug delivery systems. Generally, the polymer fragment can form a hydrophobic core to carry hydrophobic drug molecules and the DNA shell can enhance cellular uptake and introduce selectivity to targeted cells.

**Figure 31:**
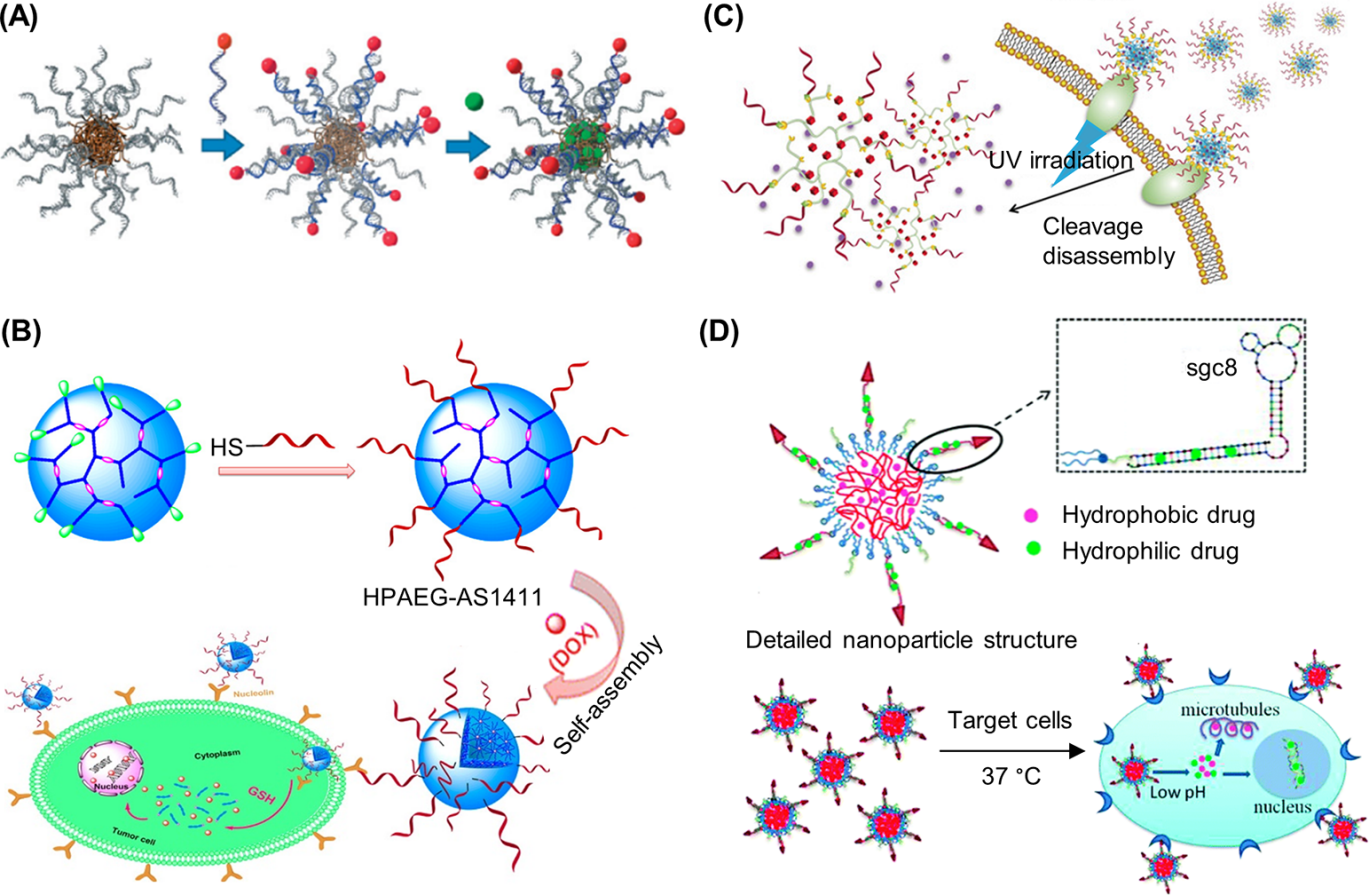
Design and development of various DNA–polymer targeted drugs based on the synergistic functionalities from polymer and DNA. (A) Schematic illustration of DNA–PPO drug delivery system.^173^ The red targeting units were introduced to the micelles by DNA hybridization, and the green anticancer drug was encapsulated into the core of the micelles. Reproduced with permission from ref (173). Copyright 2008 John Wiley and Sons. (B) DNA aptamer AS1411 was conjugated to HPAEG via the Michael addition reaction and then self-assembled to form the targeting drug delivery carrier.^119^ Adapted with permission from ref (119). Copyright 2016 American Chemical Society. (C) On-demand and controlled release of a targeted and photoresponsive drug delivery system could be achieved when UV irradiation is applied.^120^ Reproduced with permission from ref (120). Copyright 2018 John Wiley and Sons. (D) Construction of a hybrid nanoparticle-based drug delivery system. Two different drugs were codelivered into cancer cells with the targeted drug delivery system.^221^ Reproduced with permission from ref (221). Copyright 2014 the Royal Society of Chemistry.

The first targeted drug delivery system designed through DNA–polymer micelles was published by Herrmann and his colleagues in 2008.^173^ In this work, due to the proven biocompatibility toward different cell types, PPO was used as the hydrophobic component of the micelles to load the anticancer drug DOX efficiently.^218^ Subsequently folate targeting units were introduced to the micelle corona through base complementation (Figure 31A). It was demonstrated by subsequent experiments that the density of targeting units had a strong effect on cellular uptake and that the combined action of targeting units and chemotherapy drugs within the micelles resulted in cancer cells being effectively killed. Since then, there has been an influx of designs for targeted drugs based on DNA–polymer micelles.

Several special short ssDNA sequences known as aptamers have been shown to recognize cellular surface receptors and thus can be used to import the desired DNA–polymer micelles into targeted cells. Zhu and co-workers fabricated a targeted drug delivery carrier by modifying a polymer with the DNA aptamer AS1411.^119^ A new kind of hyperbranched poly(2-((2-(acryloyloxy)ethyl) disulfanyl)ethyl 4-cyano-4-(((propylthio)-carbonothioyl)-thio)-pentanoate-*co*-poly(ethylene glycol) methacrylate) (HPAEG) polymer with a backbone possessing a redox-responsive property was first successfully prepared through combining RAFT polymerization and SCVP. Then HPAEG was functionalized with AS1411 to form the drug delivery carrier (HPAEG-AS1411). Subsequently, the formed HPAEG-AS1411 nanoparticles were loaded with the anticancer drug DOX (Figure 31B). HPAEG-AS1411 nanoparticles could exhibit ascending tumor cell uptake when compared with pure HPAEG nanoparticles due to the high affinity of AS1411 to the overexpressed nucleolin on the cancer cells.^219,220^ This work confirmed that DOX-loaded HPAEG-AS1411 nanoparticles could exhibit a higher tumor cellular proliferation inhibition rate and lower cytotoxicity to normal cells, providing a new pathway for the development of targeted drug delivery for tumor therapy. Tan’s group also designed a targeted drug delivery carrier by conjugating the DNA aptamer sgc8 to the polymer.^120^ This work was the first attempt to develop a new photoresponse-based drug delivery system. The drug release system could realize controlled drug release by light mediation and be used for aptamer-targeted tumor therapy. In addition to targeting, aptamer sgc8 was used to functionalize the hyperbranched polymer (HBP) to form the assemblies and increase colloidal stability of this nanostructure. The model drug Nile Red was encapsulated into the core of the drug delivery system which could undergo cleavage under UV irradiation by employing *o*-nitrobenzyl moieties.

Once cleavage had occurred, the hydrophobicity of the drug delivery system core was rapidly reduced, causing the breakdown of the system and the resulting release of drugs (Figure 31C).

**Figure 32:**
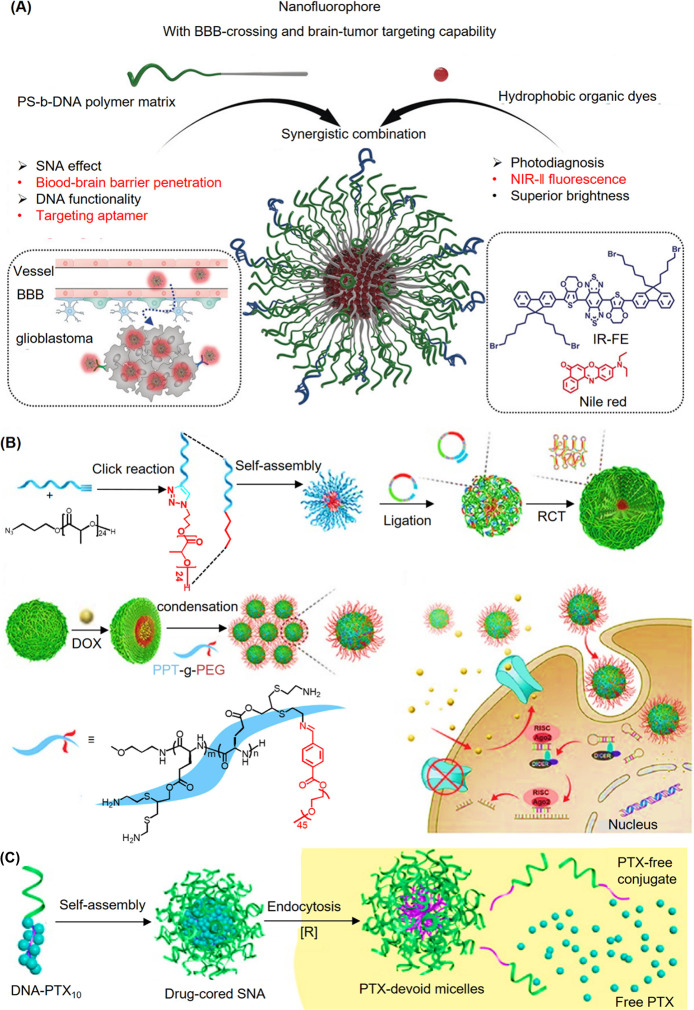
(A) Schematic illustration of spherical nucleic acids consisting of PS-*b*-DNA and NIR-II dyes.^222^ Reproduced with permission from ref (222). Copyright 2020 John Wiley and Sons. (B) Construction of drug-loaded DNA–PLA micelles and the corresponding synergistic treatment of drug resistant BC cells.^225^ Reproduced with permission from ref (225). Copyright 2018 John Wiley and Sons. (C) Schematic illustration of DNA–PTX_10_ micelles and the corresponding application.^177^ Adapted with permission from ref (177). Copyright 2016 American Chemical Society.

The two examples above mainly focused on directly modifying the polymer with aptamers to achieve the targeting effect. An alternative approach could introduce the aptamers to the surface of DNA–polymer micelles by DNA hybridization. As demonstrated by Tan’s group in 2013, the sgc8 aptamers could be introduced to the drug delivery system through hybridization of a diacyllipid-modified DNA strand.^221^ dsDNA on the hydrophilic shell formed through the hybridization of the sgc8 aptamers were used to load DOX via intercalation, while the hydrophobic PLGA core was designed to encapsulate the PTX through hydrophobic interactions (Figure 31D). The drug delivery system was successfully constructed by the assembly of PLGA and the lipid-functionalized DNA aptamer. DOX and PTX were codelivered by the constructed carriers to cancer cells in antitumor therapy. By crossing the blood–brain barrier (BBB) to deliver a second near-infrared (NIR-II, 1000–1700 nm) dye to the brain with a tumor-targeted aptamer, Tian and colleagues achieved brain-tumor imaging using DNA nanotechnology.^222^ For many years, the obstacle of the BBB limited the exploration of NIR-II nanofluorophore in the brain tumor’s imaging and diagnosis.^223,224^ In this work, the brain-tumor targeting aptamer was attached to the surface of SNA by hybridization with the DNA shell to cross the BBB (Figure 32A).^222^ NIR-II dyes could be encapsulated into the hydrophobic core of the SNA structure to be used for brain-tumor imaging. In addition, the brain-tumor targeting aptamer attached to the surface of the SNA structure could be used to increase the accumulation of the NIR-II dye in brain tumors to realize better brain-tumor imaging.

In addition to DNA aptamers, short hairpin RNA (shRNA) was used to design a targeted drug delivery carrier. Chen and co-workers constructed a new kind of nucleic acid–polymer nanodrug formulation which could be used to codeliver nucleic acid therapeutics (shRNA) and DOX (Figure 32B).^225^ ShRNA on amphiphilic DNA–polylactide (PLA) micelles was synthesized through in situ rolling circle transcription (RCT), which promoted the generation of PLA poly shRNA microflowers. This was the first time to employ in situ RCT to produce a layer of multidrug resistance protein 1 (MDR1)-silencing poly shRNA concatemers on the DNA–polymer micelle. Hydrophobic DOX was concurrently loaded into the PLA cores, and then biocompatible and multifunctional PEG-grafted polypeptides (PPT-*g*-PEG) were designed to induce microflowers electrostatically condensing into PLA-poly shRNA-PPT-*g*-PEG nanoparticles. The in vivo and in vitro experiments finally revealed the great potential of this vector in the combination of nucleic acid therapeutics and chemotherapeutics in tumor therapy.

The above work summarized that the DNA corona of DNA–polymer micelles could be functional aptamers to increase the efficiency of cell uptake. In addition, the DNA on the surface of the micelles can also be used for gene therapy. As shown in Figure 32C, Zhang’s group developed an SNA-like drug delivery system with the small-molecule drug PTX treated as the hydrophobic core of the micelle.^177^ In this work, the DNA corona of the SNA performed two functions: first as a gene therapeutic and second as a delivery vehicle for small-molecule drugs. A self-immolative disulfide linker could be introduced to this system to control the release of free drug. Multiple PTX molecules were combined to ssDNA to provide the sufficient driving force for DNA–PTX micelle formation through screening of the repulsive interactions between DNA strands.^226^ These self-assembled DNA–PTX nanostructures bypassed the need for a complex carrier system and allowed one to access a gene target and a drug target using only the payloads themselves. Additionally, as these nanostructures enable gene therapy and chemotherapy using the payloads themselves, cytotoxicity and immunogenicity challenges associated with complex vector systems are potentially avoided.

Although substantial studies have been reported on DNA–polymer conjugates as drug carriers, their application is still limited by many challenges. For instance, the stability of DNA nanostructures needs to be further improved to adapt to the complicated bioenvironment. To solve this challenge, scientists have attempted to use cationic polymers to coat DNA nanostructures to improve their stability, as described in section 5.1. However, there has been little research to confirm whether the function of DNA–polymer conjugates would be affected by polymer coating. Moreover, the poor understanding of the cytotoxicity, immunogenicity, and pharmacokinetics of DNA–polymer conjugates has also limited the development of their applications. Additionally, beyond the application as drug delivery carriers, DNA nanostructures have shown broad application prospects in fields such as in sensing, nanorobotics, and diagnostics. Hence, we can reasonably envision that DNA–polymer conjugates will also present promising future applications in these fields.

## 6 Conclusions and Outlook

The development of DNA technology, from chemical functionalization to nanoscale strategies, has seen significant refinement and breakthrough in recent years. Given the ease of access toward these methodologies, the possibilities for which DNA can be exploited have expanded far beyond conventional biology related disciplines. In particular, this review has summarized the impact of DNA on the construction of precision macromolecular conjugates and on programming supramolecular assemblies.

On the molecular scale, DNA offers a high level of customization provided by both solid phase synthesis and state-of-the-art biorthogonal chemistry thereby granting general accessibility to the community. These chemical approaches subsequently inspired the development of other reactions that can be conducted on the DNA such as polymerizations and assembly driven chemistry. Nonetheless, the technical challenge of DNA stability, its polyelectrolytic nature, and nucleophilic functional groups are still prevalent issues. These considerations become more complex at the macromolecular level as conjugation toward hydrophobic polymers/materials relies heavily on the exposure of reactive groups that are often masked by chain dynamics in solution. Nonetheless, the greater accessibility of DNA materials will ensure increasing efforts in method development.

At the nanostructure level, supramolecular interactions dictated by both the polymer and the DNA component take the central role in determining their eventual morphology. As such, polymer physics of DNA–polymer materials has overwhelming room for future innovation. In this respect, DNA offers new insights in phase transitional behavior, packing of polymer chains, and crystallinity by using precise chain conformational switches such as i-motifs and hairpins. Additionally, the balance between the ordered and monodisperse sequences of DNA against the intrinsically disordered polymer chain is a unique relationship that can foster novel nanoscience frontiers. Customization at this size regime involves polymer design and the effect of different monomers (i.e., hydrophobicity, charge interactions) in directing structure formation. Hence, most technical challenges involve irreversible aggregation of the conjugates due to incompatibility between the two blocks and the solvent system.

Structures of higher complexity rely on DNA playing a larger role in directing structure formation and hence are limited to aqueous systems. This includes highly polygonal 3D objects created by the DNA origami technology where polymer chains can be attached at any position by both *grafting to* and *grafting from* methods. Here, transference of the shape profile and information from the precise DNA scaffold to control polymerization become a powerful technique to guide polymer synthesis and orientation. However, additional restrictions with regard to stabilizing ions are imposed as polygonal DNA structures are much more fragile. Increasing efforts by exploring DNA-crossover techniques have shown optimiztic outcomes, and its broader application can be envisioned. While this review covered the aspect of polymer stabilization of these polygonal objects, it still lacks breakthrough strategies that allow broad implementations.

At each length scale, it is unambiguous how the synergy between DNA and macromolecular chemistry can bring about new horizons in multiple disciplines. However, at the same time, multiple challenges in each facet need to be overcome by the scientific community to access this knowledge. As such, every success will bring forth new technologies and features that will stimulate the collective understanding of precise nanoscopic 3D architectures in materials science and nanomedicine.

## Abbreviations

A: adenine
ACN: acetonitrile
AFM: atomic force microscopy
AGET: activators generated by electron transfer
Am: amino groups
ATRP: atom transfer radical polymerization
BBB: blood–brain barrier
B-dUTP: BODIPY-dUTP
BTPA: (((butylthio)carbonothioyl)thio) propanoic acid
C: cytosine
CMC: critical micelle concentration
CPADB: 4-cyano-4-(phenylcarbonothioylthio) pentanoic acid
CPG: controlled pore glass
CROP: cationic ring-opening polymerizations
CTA: chain transfer agent
CWC: critical water content
DBCO: dibenzo-cyclooctyne
DLS: dynamic light scattering
DMA: dimethacrylate
DMAc: dimethylacetamide
DMAm: *N*,*N*-dimethylacrylamide
DMF: dimethyformamide
DMSO: dimethyl sulfoxide
DMT: dimethoxytrityl
DNA: deoxyribonucleic acid
dNTP: deoxynucleotide triphosphate
DOX: doxorubicin
dsDNA: double stranded DNA
FBS: fetal bovine serum
FGA: frame-guided assembly
FRET: Förster resonance energy transfer
HATU: hexafluorophosphate azabenzotriazole tetramethyl uronium
HBP: hyperbranched polymer
HBTU: hexafluorophosphate benzotriazole tetramethyl uronium
HE: hexa-ethylene
HPAEG: hyperbranched poly(2-(2-(acryloyloxy)ethyl) disulfanyl)ethyl 4-cyano-4-(((propylthio)-carbonothioyl)-thio)-pentanoate-*co*-poly(ethylene glycol) methacrylate)
HPLC: high performance liquid chromatography
HRP: horseradish peroxidase
LCST: lower critical solution temperature
LHGs: leading hydrophobic groups
MALDI: matrix assisted laser desorption ionization
MDR1: multidrug resistance protein 1
NHS: *N*-hydroxysuccinimide
NIPAM: *N*-isopropylacrylamide
N-NHS: norborenyl-*N*-hydroxysuccinimide
N-MI: norborenyl-maleimide
N-PEG: norborenyl-PEG
NP: nanoparticles
ODN: oligodeoxyribonucleotide
ORN: oligoribonucleotide
P4VP: poly(4-vinylpyridine)
PAGE: polyacrylamide gel electrophoresis
PANI: polyaniline
PBS: phosphate buffered saline
PCL: polycaprolactone
PCR: polymerase chain reaction
pDAAm: poly(diacetone acrylamide)
PDI: polydispersity index
PEG: poly(ethylene glycol)
PEGDMA: PEG dimethacrylate
PEI: polyethylenimine
PIC: polyion complex
PFP: pentafluorophenol
PHEMA: poly(hydroxyethyl methacrylate)
PISA: polymerization induced self-assembly
PLA: polylactide
PLGA: poly(d,l-lactic-*co*-glycolic acid)
p(lys): polylysine
pMA: poly(methyl acrylate)
pMMA: poly(methyl methacrylate)
PNA: peptide nucleic acid
pNIPAM: poly(*N*-isopropylacrylamide)
pOEGMA: poly(oligoethylene glycol methacrylate)
pOEOMA: poly(oligoethylene oxide methacrylate)
PPMA: poly(propargyl methacrylate)
PPO: poly(propylene oxide)
PPT: polypeptides
PS: polystyrene
PtBA: poly(*tert*-butyl acrylate)
PTOTT: poly[3-(2,5,8,11-tetraoxatridecanyl)-thiophene]
PTX: paclitaxel
RAFT: reversible addition–fragmentation chain transfer polymerization
RCT: rolling circle transcription
RNA: ribonucleic acid
ROP: ring-opening polymerization
RTCBP: reversible transformation of complementary base pairing
SCVP: self-condensing vinyl polymerization
shRNA: short hairpin RNA
SNAs: spherical nucleic acids
ssDNA: single stranded DNA
St: styrene
T: thymine
TdT: terminal deoxynucleotidyl transferase
TEM: transmission electron microscopy
THF: tetrahydrofuran
TPMA: tris(2-pyridylmethyl) amine
U: uracil
UV: ultraviolet
